# General method to calculate the elastic deformation and X-ray diffraction properties of bent crystal wafers

**DOI:** 10.1107/S2052252520014165

**Published:** 2021-01-01

**Authors:** Ari-Pekka Honkanen, Simo Huotari

**Affiliations:** a Department of Physics, University of Helsinki, PO Box 64, FI-00014 Helsinki, Finland

**Keywords:** toroidally bent single crystals, spherically bent single crystals, free-electron lasers, hard X-ray spectrometry

## Abstract

Toroidally and spherically bent single crystals are widely employed as optical elements in hard X-ray spectrometry at synchrotron and free-electron laser light sources, and in laboratory-scale instruments. To achieve optimal spectrometer performance, a solid theoretical understanding of the diffraction properties of such crystals is essential. In this work, a general method to calculate the internal stress and strain fields of toroidally bent crystals and how to apply it to predict their diffraction properties is presented. Solutions are derived and discussed for circular and rectangular spherically bent wafers due to their prevalence in contemporary instrumentation.

## Abstract 2

A general method to calculate the elastic deformation and X-ray diffraction curves of arbitrarily shaped, toroidally bent crystal wafers based on the free-energy minimization is presented.

## 1. Introduction   

Crystal analysers are the heart of most contemporary mid-to-high energy resolution X-ray spectrometers in the hard X-ray regime (Suortti & Schulze, 1995 ▸; Yamaoka *et al.*, 1998 ▸). The same basic principle, the diffraction of X-rays from the periodical crystal structure, has been used in a plethora of spectrometric designs in which curved crystal analysers are employed to increase the collected photon flux and to ensure their proper focusing on a detector (DuMond & Kirkpatrick, 1930 ▸; Johann, 1931 ▸; Johansson, 1932 ▸; Cauchois, 1932 ▸; von Hámos, 1932 ▸). Especially with spherically bent crystal analysers (SBCA) one can efficiently cover and analyse photons collected over a large solid angle. SBCAs also exhibit (approximate) point-to-point focusing allowing integration of imaging and tomography capabilities in spectroscopic instruments (Huotari *et al.*, 2011 ▸). It is no wonder that many inelastic X-ray scattering (IXS) and X-ray emission spectroscopy (XES) endstations at synchrotron and free-electron laser light sources worldwide, such as SOLEIL (Ablett *et al.*, 2019 ▸), ESRF (Kvashnina & Scheinost, 2016 ▸; Huotari *et al.*, 2017 ▸; Moretti Sala *et al.*, 2018 ▸), APS (Fister *et al.*, 2006 ▸), Spring-8 (Cai, 2004 ▸; Ishii *et al.*, 2013 ▸), SSRF (Duan *et al.*, 2016 ▸), SLS (Kleymenov *et al.*, 2011 ▸), SSRL (Sokaras *et al.*, 2012 ▸) and DESY (Welter *et al.*, 2005 ▸), utilize SBCAs in their instrument designs. In addition to studying the structure and internal dynamics of matter *via* externally produced radition, SBCAs are also used to analyse X-rays in plasma research (Faenov *et al.*, 1994 ▸; Aglitskiy *et al.*, 1998 ▸; Sinars *et al.*, 2003 ▸; Knapp *et al.*, 2011 ▸).

Due to high demand and limitations of synchrotron/free-electron access, a renewed interest towards laboratory-scale X-ray instrumentation based on conventional X-ray tubes has grown in recent years (Seidler *et al.*, 2014 ▸; Anklamm *et al.*, 2014 ▸; Németh *et al.*, 2016 ▸; Holden *et al.*, 2017 ▸; Honkanen *et al.*, 2019 ▸; Jahrman *et al.*, 2019*b* ▸). Especially relevant to this work are the instrument designs based on SBCAs which, in conjunction with recent advances in the crystal technology (Verbeni *et al.*, 2005 ▸; Rovezzi *et al.*, 2017 ▸), have largely overcome the problem of low photon output per generating power limiting the previous generation of laboratory instruments (that were often based on cylindrically bent crystals, *i.e.* CBCAs). For example, Seidler *et al.* (2014 ▸) reported 4 photons W^−1^ s^−1^ for two high-resolution CBCA instruments with 2–3 eV energy resolution and 600–2000 photons W^−1^ s^−1^ for their 1 eV SBCA instrument depending on the crystal reflection used.^1^

Indeed, the portfolio of scientific cases, in which the laboratory instruments using SBCAs have proven to be a viable alternative to large-scale facilities, is expanding rapidly and spans already a vast cavalcade of interests in natural sciences such as fundamental materials research (Mortensen *et al.*, 2017 ▸), electrochemistry (Wang *et al.*, 2017 ▸; Kuai *et al.*, 2018 ▸; Sun *et al.*, 2019 ▸; Lutz & Fittschen, 2020 ▸), nanoparticle characterization (Davodi *et al.*, 2019 ▸), *in operando* battery studies (Jahrman *et al.*, 2018 ▸, 2019*c* ▸), actinide research (Bès *et al.*, 2018 ▸; Jahrman *et al.*, 2019*b* ▸; Mottram *et al.*, 2020*b* ▸), *in situ* catalysis studies (Moya-Cancino *et al.*, 2019*a* ▸,*b* ▸), geochemistry (Mottram *et al.*, 2020*a* ▸), and microbiology and enviromental research (Lusa *et al.*, 2019 ▸).

However, as a significant disadvantage SBCAs suffer from spatial separation of meridional and sagittal foci (focal astigmatism) when taken out of the backscattering condition (angle of incident X-rays <90°) which can cause aberrations in imaging and issues with detectors with small active areas. The problem may be partly averted with toroidally bent crystal analysers (TBCA) which have different sagittal and meridional bending radii. Notwithstanding, TBCAs are encountered rarely as they are more difficult to manufacture than SBCAs and need to be tuned for a specific Bragg angle which incurs increased expenses, especially if the spectrometer setup is meant to be used for a wide range of photon energies. However, at least some of these problems can be avoided by using vacuum-forming optics (Jahrman *et al.*, 2019*a* ▸) to apply the toroidal bending to a flat wafer temporarily and, perhaps with further development, dynamically in the course of an experiment.

In general, the bending process degrades the energy resolution of a TBCA/SBCA by introducing internal stress to the crystal wafer. The effect can be mitigated, for example, by dicing or cutting the wafer (Verbeni *et al.*, 2005 ▸, 2009 ▸; Shvyd’ko *et al.*, 2013 ▸). However, without a guiding theoretical understanding, such mechanical alterations might lead to unexpected adverse effects, such as loss of integrated reflectivity, optical aberrations and increased manufacturing costs. From the standpoint of instrument optimization it is thus of utmost importance to understand how the diffractive properties and the mechanical deformation of toroidally/spherically bent crystal wafer are intertwined.

The equations describing the propagation of radiation in deformed periodic medium were laid out independently by Takagi and Taupin in the 1960s (Takagi, 1962 ▸, 1969 ▸; Taupin, 1964 ▸) which alongside lamellar models (White, 1950 ▸; Erola *et al.*, 1990 ▸; Sánchez del Rio *et al.*, 2004 ▸) are routinely used to calculate the diffraction properties of bent crystals (Gronkowski, 1991 ▸; Sánchez del Río & Dejus, 2011 ▸). However, the diffraction properties of spherically bent wafers were poorly understood until the inclusion of in-plane stresses and strains to diffraction calculations in the mid-2010s. As shown in our previous work (Honkanen *et al.*, 2014*a* ▸,*b* ▸, 2016 ▸), the in-plane deformation of a thin, elastically anisotropic plate solved *via* geometrical considerations can accurately explain the experimentally measured reflectivity curves of SBCAs with circularly shaped wafers cut along arbitrary crystal directions. Nevertheless, the original derivation relies on many geometrical features and symmetries which can not be easily generalized to toroidal bending or other types of crystal shapes, such as rectangular ones used, for example, in recently introduced strip-bent analysers designed to minimized the influence of the in-plane stress (Rovezzi *et al.*, 2017 ▸).

In this work, we present a general framework to calculate internal stress and strain fields and diffracted X-ray intensities of an arbitrarily shaped, toroidally bent crystal wafer. The procedure is utilized to derive stress and strain expressions for isotropic and anisotropic circular and rectangular toroidally bent crystals due to their prevalence in the contemporary instrumentation scene. The models and their properties are discussed in detail and the accuracy of the predicted diffracted X-ray intensities is validated by comparison with experimental data. The Python implementation of the models is briefly introduced.

## 2. General theory   

The propagation of electromagnetic radiation in a crystalline medium strained by external forces is mathematically described by a group of partial differential equations known as the Takagi–Taupin equations (Takagi, 1962 ▸, 1969 ▸; Taupin, 1964 ▸). To accurately compute the intensity of X-rays diffracted by the crystal as a function of incident wavelength or incidence angle (*i.e.* the diffraction curve of a bent crystal due to a particular set of crystalline planes), the partial derivatives of the displacement vector field need to be known over the diffraction domain.

**Figure 1:**
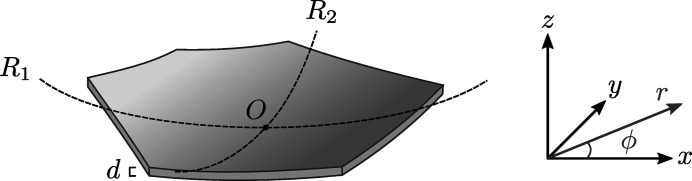
Nomenclature for a toroidally bent wafer. The thickness of the wafer is *d*. The origin *O* of the Cartesian coordinate system (*x*, *y*, *z*) and the polar coordinates (*r*, ϕ) are located at the midplane of the crystal in the *z* direction.

Consider an arbitrarily shaped, toroidally bent crystal wafer with the thickness *d*. We choose a Cartesian coordinate system (*x*, *y*, *z*) so that *z* = 0 is located at the midplane of the wafer and the radii of curvature *R*_1_ and *R*_2_ are aligned with the *x* and *y* axes as presented in Fig. 1 ▸. Since the transverse (*x, y*) dimensions of the crystal are typically small compared with *R*_1_ and *R*_2_, we may approximate the vertical (*z*) deflection ζ of the wafer with a paraboloidal surface:
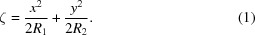
When ζ is small enough compared with the transverse dimensions of the wafer, the strain field inside the wafer can be accurately described by the pure bending model (Chukhovskii *et al.*, 1994 ▸). Let *S_ij_* be the Cartesian components of the compliance matrix of an elastically anisotropic crystal in the Voigt notation.^2^ We assume that *S_ij_* are rotated to match the orientation of the crystal directions of the wafer. As detailed in Section S1 of the supporting information, the partial derivatives of the displacement vector 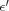 in the scope of the pure bending model for ζ given by equation (1) for an arbitrary elastically anisotropic crystal are
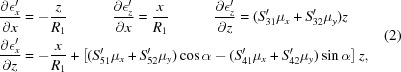
where
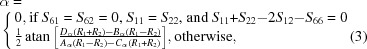
with
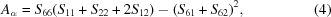


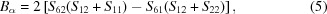


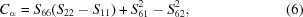


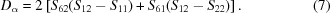
The scaled torques are given by
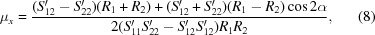


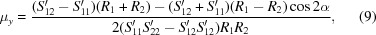
and the primed 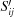 are constructed according to the Voigt notation from the rotated compliance tensor
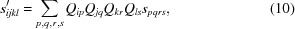
where
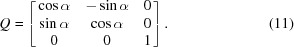
However, the pure bending solution [equation (2)] alone is inadequate to explain the diffraction curves of TBCAs as shown experimentally for SBCAs with large surface areas (Verbeni *et al.*, 2009 ▸; Honkanen *et al.*, 2014*b* ▸; Rovezzi *et al.*, 2017 ▸). This is because, in addition to pure bending strain, a flat crystal wafer is also stretched and compressed in the transverse (in-plane) directions in order to fit on a toroidal surface. These deformations affect the *d* spacing of the Bragg planes due to the non-zero Poisson ratio and thus the diffraction curve of the TBCA. In the scope of linear elasticity, the total strain tensor is 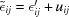 where, in addition to the pure bending strain 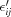, we include the ‘stretching component’ *u_ij_*.

The deformation of the wafer can be found by minimizing the mechanical strain energy under the toroidal bending constraint. If we assume that the wafer is thin (*i.e.* the transverse dimensions of the wafer are considerably larger than its thickness *d*), the total energy can be written as the sum of the pure bending energy and the stretching energy. Since the pure bending solution is already known, it is sufficient to concentrate on the minimization of the stretching energy given by
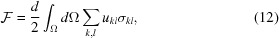
where the integration goes over the wafer surface Ω and the sum includes only the transverse indices *x* and *y*. Using the Hooke’s law 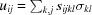 and the Voigt notation, we can rewrite equation (12) as
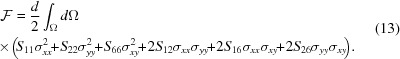
The strain tensor must fulfil the equilibrium condition 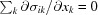 which is ascertained if we express σ_*ij*_ as a function of χ = χ(*x*, *y*), also known as the Airy stress function, so that
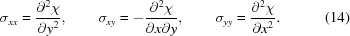
For a thin deflected wafer, *u_ij_* can be assumed to be constant with respect to the *z* coordinate given by equation 14.1 (p. 51) in the work by Landau *et al.* (1986 ▸):
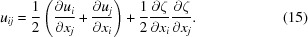
Combining equation (15) with equation  (14) and Hooke’s law allows us to write the constraint for the toroidal bending:
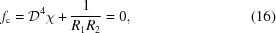
where
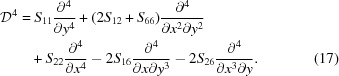
In addition, since contact force *P* (per unit area) between the wafer and substrate is the only external force acting on the wafer, we require that its integral over the surface has to vanish in order the wafer to stay stationary, *i.e.*

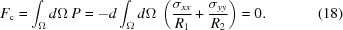
The detailed derivation is presented in Section S1 of the supporting information.

To find χ(*x*, *y*) which minimizes equation (13) under the constraints given by equations (16) and (18), we utilize the fact that the dimensions of the wafer are small compared with the bending radii *R*_1,2_. Therefore we may write the ansatz in powers of *x*/*R*_1_ and *y*/*R*_2_ and truncate the series after a few lowest-order terms reducing the problem to finding a finite set of expansion coefficients *C_k_*. The solution is found by defining a new functional 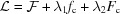 and finding a set 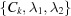 which solves the linear system
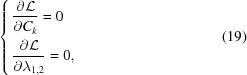
when the expansion of χ is limited up to the fourth order.^3^ The σ_*ij*_ from equation (14) are then substituted into Hooke’s law to calculate *u_xx_, u_xz_* and *u_zz_* needed for the diffraction calculations.

**Figure 2:**
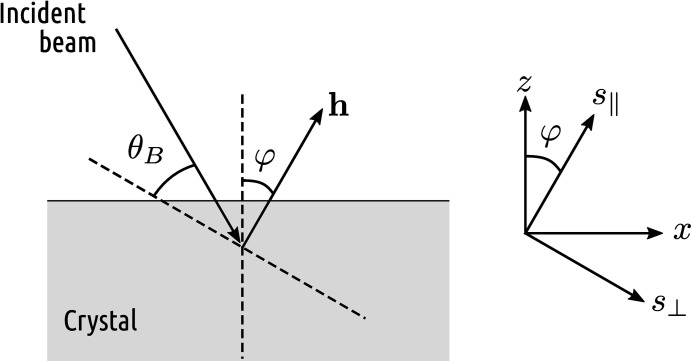
Illustration of diffraction related quantities. **h** is the reciprocal vector of the diffraction, θ_B_ is the Bragg angle, φ is the asymmetry angle, and *s*_parallel_ and *s*_perp_ are coordinate axes parallel and perpendicular to **h**. *x* and *z* belong to the Cartesian coordinate system presented in Fig. 1 ▸.

The total strain field of the pure bending and stretching components could be directly used as a deformation term in the Takagi–Taupin equations but it is computationally unfeasible for a three-dimensional macroscopic crystal. However, as shown in our previous work (Honkanen *et al.*, 2016 ▸), the problem can be substantially reduced by solving the diffraction curve from the Takagi–Taupin equations using the depth-dependent bending strain 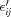 and convolving the resulting curve with the contribution due to the stretching strain *u_ij_* which is assumed to be constant in the diffraction domain of any single ray. Assuming that the incidence angle of the X-rays are fixed and the wavelength is varied, the mean wavelength λ of the pure bending diffraction curve is changed due to *u_ij_* by an amount Δλ according to equation (11) in our previous work (Honkanen *et al.*, 2016 ▸):
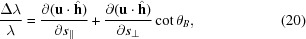
where **u** is the displacement vector corresponding to *u_ij_*, 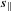 and 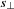 are directions parallel and perpendicular to the reciprocal lattice vector **h** (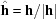), and θ_B_ is the Bragg angle, as presented in Fig. 2 ▸. Assuming that the diffraction takes place in the *xz* plane, equation (20) can be written in terms of photon energy 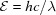 and strain tensor components:
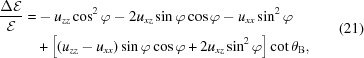
where φ is the asymmetry angle. The details are presented in Section S1 of the supporting information. In the symmetric Bragg case, equation (21) simplifies to
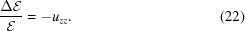
The diffraction (or resolution) curve of the whole crystal wafer is then obtained by calculating the distribution 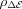 of energy shifts 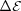 over the surface and convolving the resulting distribution with the 1D Takagi–Taupin curve solved for the pure bending solution in equation (2). Formally 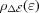 for a particular energy shift ε is obtained by summing all the surface elements *d*Ω whose energy shift 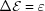, *i.e.*

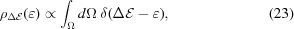
where δ is the Dirac delta function and 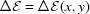 is understood to be a function of position. Similarly, for rocking curve measurements with a monochromatic beam, the shifts in the diffraction angle are
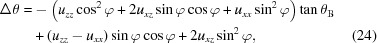
which in the symmetric Bragg case simplifies to
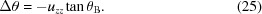
Note that equation (25) ceases to be valid near θ_B_ = π/2 since it is based on the first-order Taylor expansion.

Usually changes in both 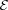 and tanθ_B_ are minute during scans which means that they can be considered constants. Thus Δ*E*, Δθ and their corresponding distributions differ only by a multiplicative factor. Therefore only the Δ*E* distributions are presented in the following section.

## 3. Important special cases   

In this section we apply the general framework to derive the transverse stretching strain and stress fields caused by toroidal bending for circular and rectangular wafers which are of particular relevance considering the current trends in the contemporary instrument design. For the sake of brevity, only the initial assumptions and the results are discussed. The detailed derivations are presented in Section S2 of the supporting information.

### 3.1. Isotropic circular wafer   

Consider a toroidally bent, isotropic circular crystal wafer with the thickness *d*, diameter *L* and bending radii *R*_1_ and *R*_2_. It turns out that the problem is mathematically equivalent to spherical bending with a bending radius of *R* = (*R*_1_ *R*_2_)^1/2^ which implies radial symmetry of χ. This allows us utilize the general solution to ∇^4^χ = 0 known as the Mitchell solution (Michell, 1899 ▸) to find the exact solution instead of a mere polynomial approximation. By solving the linear system in equation (19), the components of stretching stress tensor are found to be
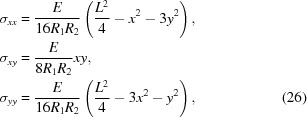
where *E* is Young’s modulus. The contact force per unit area between the wafer and the substrate is thus
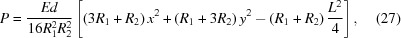
and the components of *u_ij_* are
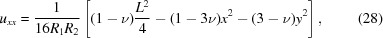


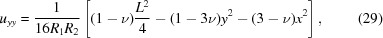


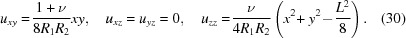
Using equation (22), we find that the energy shift 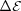 as a function of surface position in the polar coordinates (see. Fig. 1 ▸) is
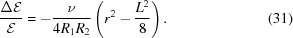
The isocurves of the energy shift are circular as one would expect on the basis of radial symmetry. Substituting 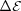 obtained into equation (15) and carrying out the integration, we find the energy shift distribution
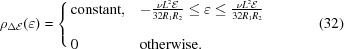
The found uniform distribution can be used to convolve the 1D Takagi–Taupin solution to predict the diffraction curve of a TBCA.

To quickly estimate the effect of stretching strain on the energy resolution, we note that the variance of a uniform distribution with a width of *w* is *w* ^2^/12 and thus the standard deviation of the energy shift distribution [equation (32)] is
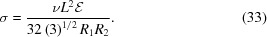
The standard deviation due to stretching strain can then be combined with the standard deviations of other contributions (1D Takagi–Taupin, incident bandwidth, *etc*.) by quadratic summing in accordance with the central limit theorem. Usually the full width at half-maximum (FWHM) is used instead of the standard deviation, in the case whereby σ is to be multiplied by 2(2ln2)^1/2^. This underestimates the true FWHM of equation (32) approximately by a factor of 0.68 but, regarding the central limit theorem, gives a more accurate contribution to the total FWHM.

### 3.2. Anisotropic circular wafer   

For the anisotropic case, we ought to not assume *a priori* that χ is radially symmetric as it is in the isotropic case. However, it turns out that applying the minimization procedure to a general fourth-order polynomial ansatz yields a solution for the stretching stress tensor that is effectively equivalent to that of the isotropic case:
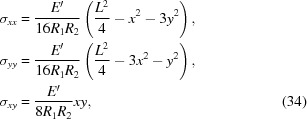
where
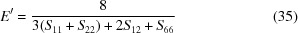
can be interpreted as the effective Young’s modulus. Note that, for an isotropic crystal, *E*′ = *E*, but in general *E*′ ≢ 1/*S*_11_. The contact force is thus equivalent to equation (27) when *E* is replaced with *E*′. The components of *u_ij_* relevant to the diffraction calculations are obtained by substituting the stresses from equation (34) into Hooke’s law:
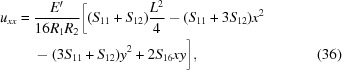


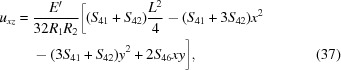


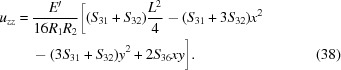
The stress tensor in equation (34) expressed in the polar coordinates is
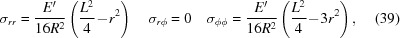
which clearly exhibits radial symmetry. This is expected since anisotropy along ϕ would even itself out, as argued in our previous work (Honkanen *et al.*, 2014*b* ▸). However, this symmetry is broken in the strain tensor due to the anisotropic elastic properties of the crystal. In the symmetric Bragg case, the energy shifts in terms of the polar coordinates are
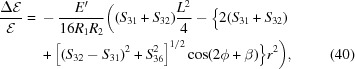
where 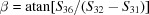. Generally the isocurves of 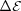 are elliptical whereas for the isotropic case they are circular. The derived expression for 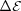 is otherwise similar to our previous result (Honkanen *et al.*, 2014*b* ▸) except for the constant term proportional to *L* ^2^. The discrepancy arises from the fact that our previous approach was based solely on the geometrical considerations of the spherical bending neglecting the elastic energy of the wafer. The difference between the diffraction curves of the old and new methods is not large but the new approach does remove some non-physical features of the old one (*e.g.* the non-zero integrated contact force at the wafer/substrate-interface). The derivation presented in this work is theoretically more sound and thus expected to be physically more accurate. However, it should be noted that the original approach leads to the same solution if the integrated contact force is required to vanish.

**Figure 3:**
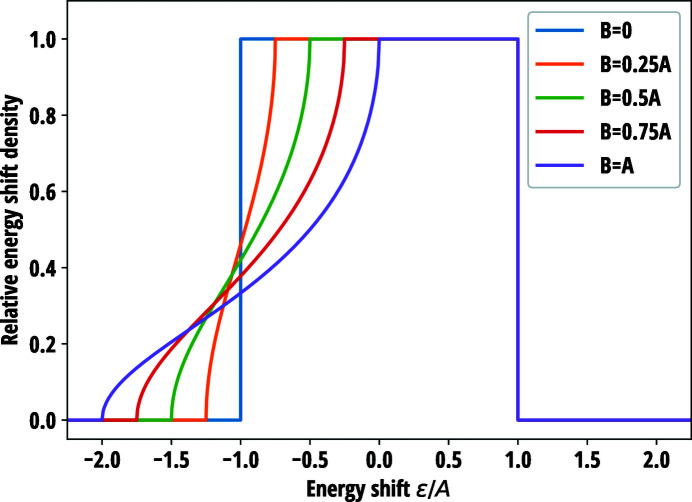
Distribution of energy shifts 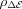 as a function of scaled photon energy 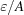 for an anisotropic circular wafer for various values of *B*. *A* and *B* are parameters related to the width of the distribution and the in-plane anisotropy of Poisson’s ratio, respectively, as defined by equation (42).

**Figure 4:**
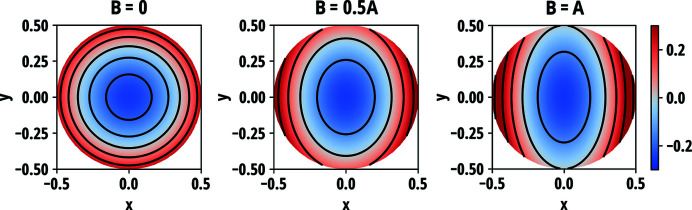
Distribution and isocurves of the energy shifts over the anisotropic circular wafer for three different *B*/*A* ratios which is a quantity directly related to the in-plane anisotropy of Poisson’s ratio and thus to the ellipticity of the isocurves. The gradient of the energy shifts is steepest along the *x* axis.

Substituting equation (40) into equation (23) gives the following energy shift distribution,
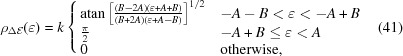
where *k* > 0 is a proportionality constant and
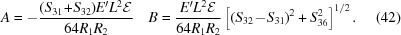
Plots of equation (41) with selected values of *B*/*A* are presented in Fig. 3 ▸. When *B* = 0, the isocurves of Δ*E* are circular as in the isotropic circular case. For non-zero values of *B*, the isocurves become elliptical and are intercepted by the circular edge as illustrated in Fig. 4 ▸. The discontinuous isocurves introduce a tail on the low-energy side of the Δ*E* distribution, the prominence of which is proportional to the *B*/*A* ratio. The standard deviation of equation (41) for energy resolution estimation is
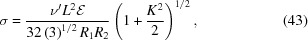
where we have introduced the effective Poisson’s ratio
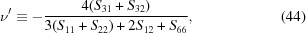
and the eccentricity factor
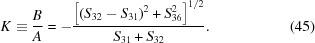
Values of ν′ and *K* for selected crystal directions of Si and Ge are tabulated in Table S1 of the supporting information. In the isotropic case, ν′ = ν and *K* = 0, thus reducing equation (43) to equation (33) as expected.

An important practical implication of elliptical isocurves is that there is a specific direction along the surface in which the energy shift varies fastest. Since *S*_31_ and *S*_32_ are negative, the gradient of Δ*E* as per to equation (40) is steepest in the radial direction when 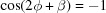 (*i.e.* 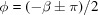). This has relevance in regards to the resolution function in cases where the surface area of a TBCA needs to be limited transversally in one direction, for example, to minimize the Johann error by masking the surface, or to reduce the space occupied by the analyser by cutting its edges off. To optimize the intrinsic resolution of the analyser, the surface area should be reduced where the gradient is steepest.^4^ For example, masking the edges of a spherical Si(660) analyser with 100 mm diameter and 1 m bending radius using a 80 mm-wide slit can improve the energy resolution (measured from the standard deviation) by 13% in near-backscattering conditions if the mask is aligned over the direction of the steepest gradient, which is [110]. However, in the worst-case scenario when the mask is oriented perpendicular to the optimal case, the resolution ‘degrades’ by 3% in comparison with the unmasked crystal. In the worst case, the resolution of the SBCA in question can thus be 18% worse than with optimal masking/cutting which is not a negligible detriment. The directions of steepest gradient for selected crystal planes in cubic systems are listed in Table S1.

**Figure 5:**
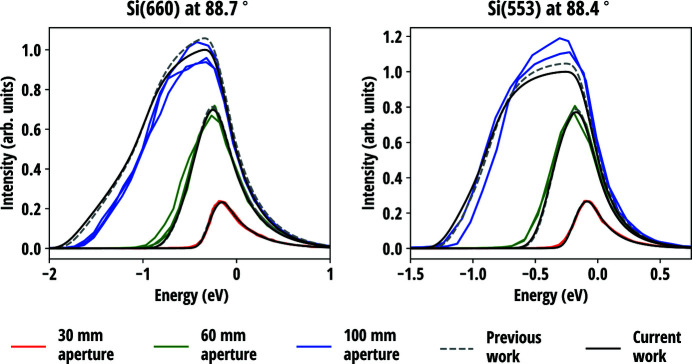
Measured reflectivity curves of 3 Si(660) and 2 Si(553) SBCAs compared with the predictions of the current and previous work (Honkanen *et al.*, 2014*b* ▸). The bending radii were 1 m and the wafer thicknesses were 300 µm. The theoretical curves are convolved with contributions from the incident bandwidth and Johann error. The centroid energy and the vertical scale of the curves were adjusted as a group to optimize the fit between the theoretical and experimental curves with a 30 mm aperture.

**Figure 6:**
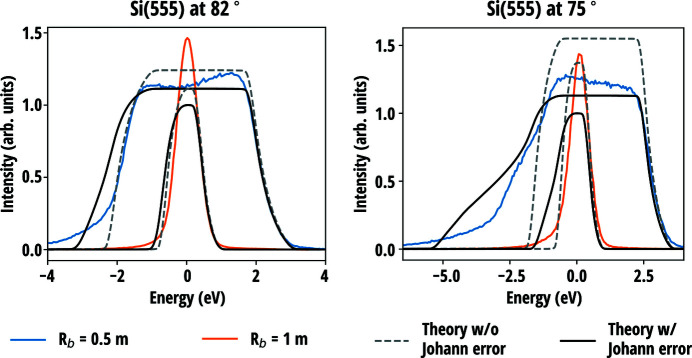
Calculated reflectivity curves of two circular Si(555) SBCAs with bending radii of 0.5 m and 1 m at two different Bragg angles in comparison with experimental curves (Rovezzi *et al.*, 2017 ▸). The wafer diameters were 100 mm and the thicknesses 150 µm. The centroid energy of the theoretical curves were adjusted separately for 1 m and 0.5 m analysers. The ratio of theoretical integrated intensities of the two SBCAs were scaled according to their solid angle multiplied with their integrated 1D Takagi–Taupin reflectivities.

The predictions of the anisotropic circular model were calculated for four different types of SBCA and compared with two separate experimental datasets acquired at ESRF and first published by us (Honkanen *et al.*, 2014*b* ▸) and Rovezzi *et al.* (2017 ▸). Fig. 5 ▸ presents the reflectivity curves measured in near-backscattering conditions from three Si(660) and two Si(553) analysers all with the bending radius of 1 m, 100 mm diameter and 300 µm wafer thickness. The curves were acquired using two circular masks with aperture diameters of 30 mm and 60 mm, and without a mask (aperture 100 mm). Fig. 6 ▸ presents the comparison of the current model with and without the contribution of the Johann error (Johann, 1931 ▸) to the reflectivity curves measured at two different Bragg angles of two Si(555) circular analysers with bending radii of 1 m and 0.5 m. The diameter and thickness of the wafers were 100 mm and 150 µm, respectively. Further experimental details are presented in the original sources.

Compared with previous work which was based on the geometrical considerations and did not account for the minimization of the elastic energy, slight differences between two models are observed but they are found to be less than the variation between different SBCA units, as seen in Fig. 5 ▸. This excludes one explanation put forward in previous work for the discrepancy between the data and the model at the low-energy tail of the diffraction curve for the full analyser, according to which the observed difference could be attributed to to non-vanishing σ_*rr*_ at the wafer edge in the previous model. One possible explanation to the discrepancy is the imperfections in the manufacturing process, as it is found that the figure error in anodically bonded analysers is largest at the edge (Verbeni *et al.*, 2005 ▸). Another explanation could be a slight deviation from the Rowland circle geometry that is not included in the calculations. The latter hypothesis is supported by the data in Fig. 6 ▸ where the deviations are more prominent. According to the theory, the stresses and strains caused by stretching are a factor of four larger in a wafer that has half the bending radius than in a wafer otherwise identical. Even for considerably higher in-plane stress, the theory predicts correctly the observed boxcar shape and its width for the measured 0.5 m Si(555) analyser. The general shape and the width of the predicted 1 m Si(555) curve are in line with the measurements but are not as precise as for the set of Si(660) and Si(553) analysers in Fig. 5 ▸. The most probable reason for this is the contribution of the aforementioned deviation from the Rowland circle geometry, the effect of which is amplified at lower Bragg angles. In the experimental description, it is mentioned that the radius of the Rowland circle was adjusted by optimizing the product of total counts and peak intensity divided by the FWHM for each analyser (Rovezzi *et al.*, 2017 ▸). Since the different contributions to the energy resolution of an SBCA are not truly independent of each other, such an optimization can lead to partial cancellation of some contribution by another and thus lead to a better resolution than expected in the exact Rowland circle configuration. Therefore, to accurately characterize the elastic contribution to resolution functions of SBCAs, the near-backscattering condition is recommended to minimize the geometrical effects.

### 3.3. Isotropic rectangular wafer   

**Figure 7:**
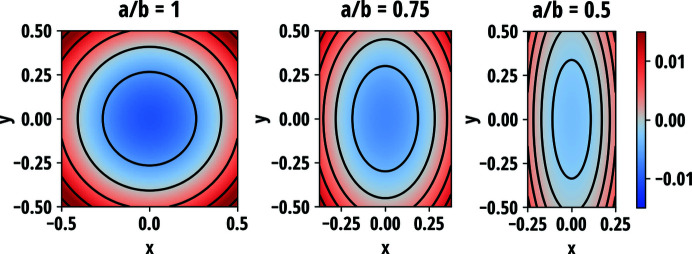
*u_zz_* component of the stretching strain tensor for three different wafer side-length ratios *a*/*b*. Poisson’s ratio ν = 0.25 was used. Positive (red) values indicate expansion and negative (blue) values indicate contraction of the crystal normal to the surface. Black lines indicate the isocurves of *u_zz_*.

We assume that a toroidally bent, rectangular crystal wafer is centred at *x* = *y* = 0 with sides of length *a* and *b* aligned parallel with the *x* and *y* axes, respectively. To simplify the problem, we note that the system is symmetric under transformations 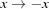 and 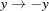 and thus immediately conclude that the polynomial expansion of χ can contain only terms in which the powers of *x* and *y* are both even. As a result of minimization, we find the following stress tensor components
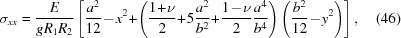


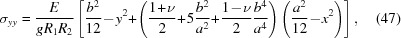


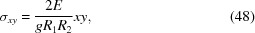
where
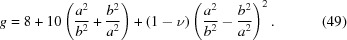
The contact force per unit area is
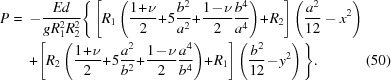
Substituting equations (46)–(48) into Hooke’s law, the relevant strain tensor component for the diffraction calculations is thus found to be
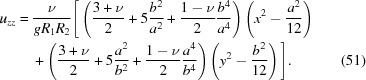
Equation (51) for three different *a*/*b* ratios is visualized in Fig. 7 ▸. In general, the crystal planes normal to the surface are compressed in the centre of the wafer and expanded at the edges, which is reactionary to in-plane extension at the centre and contraction at the edges of the wafer via non-zero Poisson’s ratio. The isocurves of *u_zz_* are found to be elliptical in shape, albeit being cut near the edges of the wafer. The major axis of the isocurves are along the longer dimension of the wafer and the strain grows fastest along the minor axes. For the special case of *a* = *b*, the isocurves become circles following the symmetry of the crystal similar to the isotropic circular wafer. It is interesting to note that, although in the case of circular wafer, non-circular isocurves are a result of the breaking of radial symmetry by anisotropy of elastic properties of the crystal, for the rectangular wafer it is broken by lifting the 90° rotation symmetry.

When *a* < *b*, the distribution of 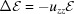 is found to be
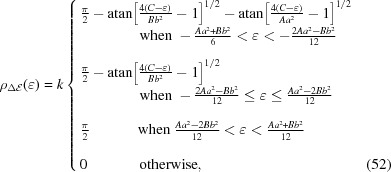
where *k* > 0 is the proportionality constant and
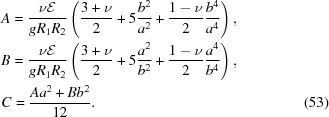
The case of *a* > *b* is identical to equation (52) provided that all *Aa* ^2^ are replaced with *Bb* ^2^ and vice versa. The standard deviation of the distribution in equation (52) is approximately given by
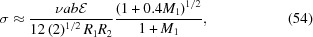
where
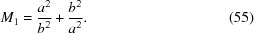
Equation (54) is accurate within a few precent over the range 0 < ν < 1 being near exact for ν = 0.5.

**Figure 8:**
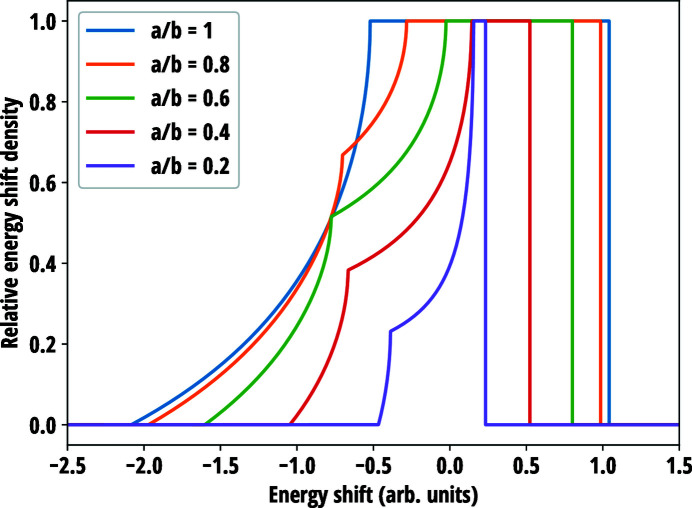
Effect of wafer side-length ratio *a*/*b* on the energy shift distribution due to transverse strain in an isotropic rectangular crystal. The area of the wafers was kept constant but for visual clarity the curves are normalized to the maximum instead of the integrated area. Poisson’s ratio ν = 0.25 was used.

Examples of energy shift distribution given by equation (52) are presented in Fig. 8 ▸ for rectangular wafers with constant area but various side-length ratios. As in the anisotropic circular case, distribution has a flat portion consisting of complete elliptical isocurves and a left-hand-side tail caused by the isocurves cropped by the wafer edges (see Fig. 7 ▸). When *a* ≠ *b*, the tails exhibit a non-differentiable kink due to the isocurves being cropped at different energy shifts along the minor and major axes. Keeping *a*/*b* constant, the width of the curve scales proportional to the surface area of the wafer or, equivalently put, to the second power of its linear dimensions and to good accuracy it is directly proportional to the Poisson ratio.

### 3.4. Anisotropic rectangular wafer   

The solution for the anisotropic rectangular wafer can be found using the forth-order polynomial ansatz for χ. The analytical solution to the minimization exists but is too complicated to be practical. Therefore the expansion coefficients of χ are best found numerically. However, the analytical solution can be used to reduce the number of unknowns in the linear system presented in the Appendix[App appa].

**Figure 9:**
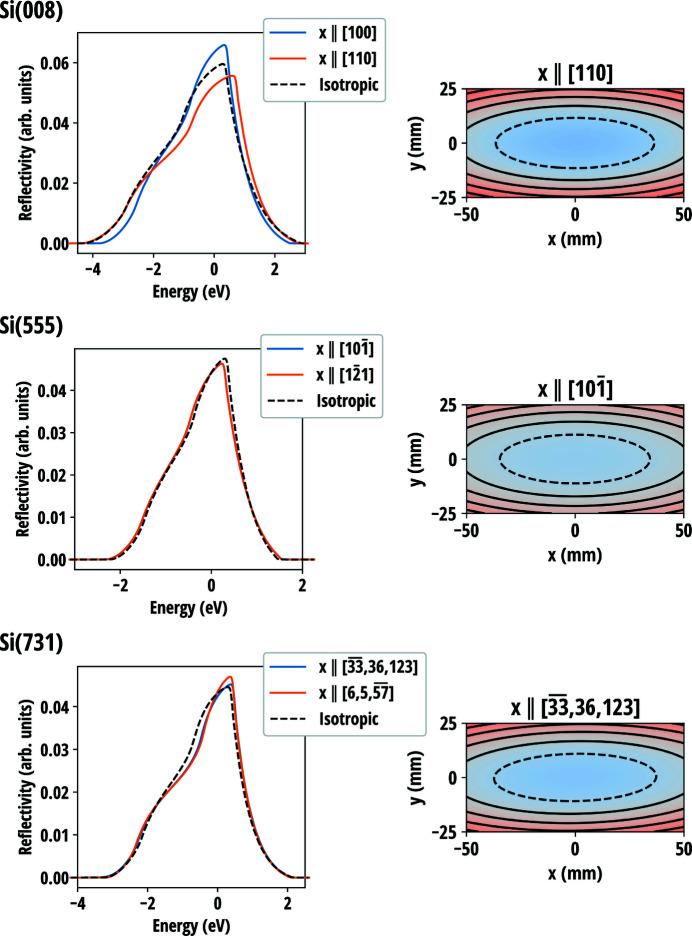
(Left column) Resolution curves of rectangular wafers for three different reflections of Si with selected in-plane crystal orientations aligned with the *x* axis in comparison with the isotropic model. Note that for Si(555) the curves overlap and the integer indices for Si(731) in-plane directions are approximate. The dimensions of the wafers were set to 100 mm × 50 mm × 150 µm with the long edges aligned with the *x* axis. The bending radius was set to 0.5 m and the Bragg angle was 88.5°. The Johann error has been omitted. (Right column) *u_zz_*-component of the strain tensor over the crystal surface. Red indicates expansion and blue indicates contraction. Isocurves are marked with solid and dashed black lines.

The anisotropic model was used to calculate the diffraction curves of symmetric Si(008), Si(555) and Si(731) reflections with selected in-plane crystal directions as presented in Fig. 9 ▸. The anisotropic curves are compared with the isotropic model where Poisson’s ratios 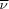 were taken to be the means of 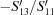 over 2π in-plane rotation for each reflection. The isotropic curves are found to follow their anisotropic counterparts rather well. Unlike for the anisotropic circular wafer, the shape of the resolution curve does not seem to change considerably between different reflections even though their width varies. This indicates that the shape of the resolution curve is largely determined by the aspect ratio of the wafer and its width is scaled by the (effective) Poisson’s ratio in both isotropic and anisotropic cases.

However, the isotropic model fails to capture some details in the reflectivity curves, most notably the effect of the in-plane orientation of the crystal which for some reflections [*e.g.* Si(008)] can cause a significant effect to the resolution curve of the crystal. Nevertherless, as it is evident from equations (57)–(59), the isocurves of the transverse stresses, and thus the strains as well, are elliptical in shape as they are in the isotropic case, although for some crystals and orientations the main axes of the ellipses may be inclined with respect to the sides of the wafer, as seen for Si(731) in Fig. 9 ▸.

For the investigated reflections, the isotropic model with in-plane averaged Poisson’s ratio 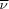 appears to be a reasonable approximation to the anisotropic one at least for cubic systems. Further work is needed to extrapolate the conclusion to other crystal systems.

### 3.5. Strip-bent crystal analyser   

**Figure 10:**
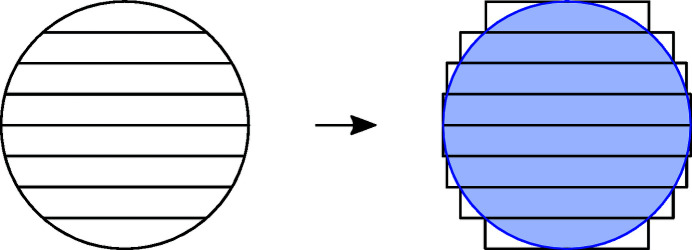
Approximation of the strip-bent analyser using rectangular strips. The wafer is divided into narrow rectangular slices which cover the whole surface area of the circular analyser. The excess parts of the strips are neglected in the approximation.

As seen in Fig. 6 ▸, the transverse stretching can cause a contribution of several electronvolts to the FWHM of the resolution function which is unacceptably large for many spectroscopic purposes. To mitigate the effect of the transverse strain, the surface of the circular wafer can be cut into thin strips before bonding the wafer onto the toroidal substrate. The diffraction properties of such strip-bent analyser can be estimated by approximating the strips by rectangular wafers as presented in Fig. 10 ▸. Such an approximation is expected to be most accurate at the centre of the analyser where the strips are nearly rectangular in shape. The strips at the edge deviate more from the rectangular approximation but also contribute less to the total diffraction curve due to smaller surface area.

Some freedom exists in choosing how to approximate the strips with rectangular wafers. We chose to cover the strips fully and mask out the parts extending over the circular wafer. This ensures that the approximating strips have the surface area equal to the real strips and allows geometrical errors, such as the Johann error, to be modelled accurately.

**Figure 11:**
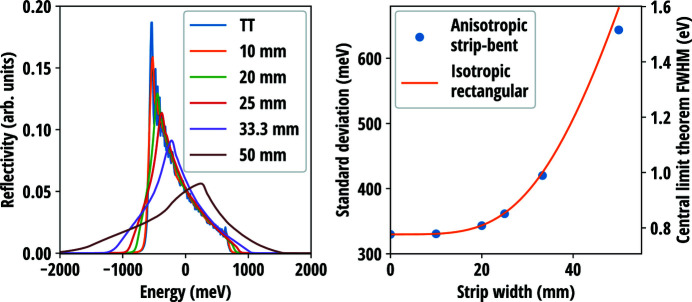
(Left panel) Resolution curves of the Si(555) strip-bent analyser with various strip widths compared with the pure bending Takagi–Taupin (TT) solution. The diameter of the analyser was set to 100 mm, the bending radius to 0.5 m and the wafer thickness to 150 µm. The Bragg angle was chosen to be 88.5° and the Johann error was neglected. (Right panel) Standard deviations/central limit theorem FWHMs of the resolution curves compared with the prediction based on the expression 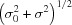, where σ_0_ is the standard deviation of the pure bending TT-solution and σ is calculated from the isotropic rectangular wafer model [equation (54)] with the in-plane averaged Poisson’s ratio 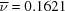.

The left panel of Fig. 11 ▸ presents the calculated resolution curves of strip-bent Si(555) analysers with a bending radius of 0.5 m, diameter of 100 mm and wafer thickness of 150 µm at near-backscattering conditions for various strip widths. The strip widths are chosen so that the surface can be divided into an integer number of strips of equal width. As expected, the width of the resolution curve decreases as the strips become narrower and eventually approach the pure bending TT-solution. The standard deviations of the resolution curves are presented in the right panel of Fig. 11 ▸. Plotted with the standard deviations is the predicted behaviour according to 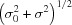, where σ_0_ is the standard deviation of the pure bending solution and σ is given by the analytical expression [equation (54)] for the isotropic rectangular wafer with the side lengths taken to be strip width and the diameter of the analyser, respectively. Poisson’s ratio is taken to be 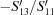 averaged over 2π in-plane rotation. A good correspondence between the fully anisotropic widths and the semianalytical model is found.

The resolution curves of the state-of-art strip-bent Si(555) analysers manufactured using the anodic bonding techinique were reported in the work by Rovezzi *et al.* (2017 ▸). The strip width of the analysers was 15 mm, other physical parameters matched those used in the calculations of Fig. 11 ▸. Based on the simulations, the transverse stretching begins to contribute notably to the resolution only after the strip width is larger than 20 mm, which means that the stress of the reported analysers is optimally mitigated. The experimental data indeed show no significant contribution from the transverse strain. From the viewpoint the rectangular wafer and strip-bent model validation, this unfortunately makes a more detailed comparison between the theoretical predictions and the data uninformative.

## 4. Reference implementation   

Two open source Python packages, pyTTE and tbcalc, are provided for the low-threshold adoption of the methods to predict the resolution functions of the bent isotropic and anisotropic crystal wafers presented in Section 3. pyTTE calculates 1D X-ray diffraction curves of elastically anisotropic crystals with a depth-dependent deformation field in Bragg and Laue geometries by solving the 1D Takagi–Taupin equation using the variable-coeffient ordinary differential equation solver (VODE) with the backward differential formula (BDF) method (Brown *et al.*, 1989 ▸) as implemented in the *SciPy* library (Jones *et al.*, 2001 ▸). The xraylib library (Schoonjans *et al.*, 2011 ▸) is utilized for X-ray diffraction and crystallographic data; tbcalc implements the toroidal bending models to calculate the stretching stress and strain fields and their effect on the diffraction curves of isotropic and anisotropic circular and rectangular wafers and strip-bent analysers. The source codes are freely available online at https://github.com/aripekka/pyTTE and https://github.com/aripekka/tbcalc.

## 5. Discussion   

Compared with previous work (Honkanen *et al.*, 2014*a* ▸,*b* ▸), the constrained elastic energy minimization approach presented in Section 2 offers a straight-forward and general approach to predict the diffraction curves of arbitrarily shaped toroidally bent crystal wafers. Since toroidal bending encompasses spherical, paraboloidal and cylindrical bendings, and it can be used as an approximant to many other types as well, the new theory is applicable to the vast majority of crystal optics based on thin, single-crystal wafers. In this work we have focused solely on X-ray diffraction properties, but since the Takagi–Taupin theory applies also to neutron diffraction, the method can be extended to neutron optics with minor modifications.

Analytical solutions derived in Section 3 provide insight into the properties of most commonly encountered circular and rectangular TBCAs enabling both detailed simulations and quick ball-park estimations of the energy resolution. However, the integration domains in the free-energy minimization can be easily extended to arbitrarily shaped wafers with numerical methods, thus making it possible to simulate even the most unorthodox crystal shapes in search for the optimal instrument performance.

Nevertheless, even though the method rests on a solid theoretical foundation and is internally consistent, more experimental verification is still needed. Ideally, in order to minimize other effects to the resolution curve, the experiment would be performed in near-backscattering conditions with a σ-polarized beam and the diffraction curve would be mapped out as a function of position on the crystal surface either using a tightly focused beam or a mask with small aperture in front of the crystal.

One of the main assumptions in calculating the transverse stretching is that the wafer is (infinitely) thin and of even thickness throughout. However, in practice the wafer is of finite thickness which may vary along the wafer. This variation may be purposeful such as in the case of Johansson-type analysers (Johansson, 1932 ▸; Hosoda *et al.*, 2010 ▸), or inadvertent such as possible imperfections left behind in the manufacturing process. Such variations could be included by replacing the constant thickness *d* with a function of surface coordinates *d* = *d*(*x*,*y*) and including it in the integrals of free energy and contact force. Such an approach should work well without further modification if *d*(*x*,*y*) can be written as a low-order polynomial, like in the case of Johansson-type analysers which are ground after bending so their surfaces follow the Rowland circle exactly, but may require additional higher-order terms in the expansion of χ. Alternatively, if the variation in *d*(*x*,*y*) is small, a perturbative approach could be easier to apply. The latter approach could also be used to include also the figure and slope errors from the perfect toroidal surface due to, for example, imperfections in bonding or shape of the substrate (Blasdell & Macrander, 1995 ▸; Yumoto *et al.*, 2008 ▸; Barrett *et al.*, 2010 ▸; Thiess *et al.*, 2010 ▸). Used in conjunction with advanced wafer machining methods with optical interferometry feedback, similar to what is presented in the work by Onuki *et al.* (2011 ▸), the method could be extended to purposefully design *d*(*x*,*y*) to tune the optical parameters of the analyser. More theoretical and computational work is needed to quantify the magnitude of imperfections to the diffraction properties.

**Figure 12:**
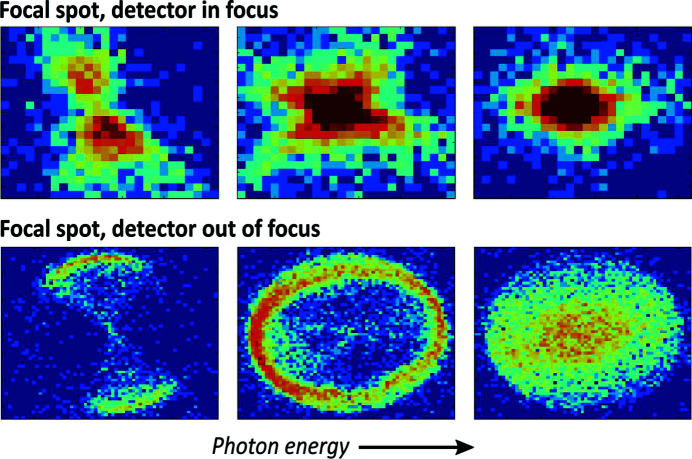
Typical focal spot of a circular Si(660) SBCA with a bending radius of 1 m and diameter of 100 mm measured in near-backscattering conditions with a position sensitive detector as a function of photon energy. The pixel size is 55 µm and the colour represents the recorded photon counts on the logarithmic scale. In the top figure the detector was positioned at the focal spot of the SBCA and in the bottom figure it was moved out of focus, effectively mapping the diffracted intensity as a function of surface. The figure is a previously unpublished image from the experimental dataset used in our previous work (Honkanen *et al.*, 2014*b* ▸) and in Fig. 5 ▸ of the current work.

In addition to its energy or angular resolution, another important figure of merit of an crystal analyser is its focusing properties. As presented in the top panel of Fig. 12 ▸, when the resolution function of a high-quality circular SBCA is measured in the energy domain using a position sensitive detector, one can see the focal spot first appear as a faint hourglass-shaped figure at the low-energy tail of the resolution curve which then converges into a single spot as the energy is increased. In the bottom panel of Fig. 12 ▸ where the detector is moved out of focus, one can effectively map the diffractivity of the analyser as a function of surface position, revealing the elliptical shape of the energy shift isocurves similar to the centre and right panels of Fig. 4 ▸. The orientation of the hourglass pattern and the isocurves corresponds to the predicted crystal direction of steepest gradient of *u_zz_* which is a clear indication that transverse stretching can have an effect on the focusing properties of the analyser as well. However, combining the presented method with optical simulations has not been explored in depth for the time being.

From a general perspective, the energy resolution of TBCAs worsens with increasing photon energy. On the other hand, higher photon energies would be important to utilize in IXS experiments at synchrotrons because the inelastic scattering cross section increases with increasing photon energy while photoelectric absorption decreases. Higher-energy photons would also have higher penetration capability for condensed samples when combining imaging and IXS spectroscopy. These requirements with standard TBCAs are in conflict with each other. Development for higher-resolution TBCAs that operate with higher photon energies would thus be an important goal. Our results are expected to work towards that goal, giving a framework with which the optimization of the crystal analyzers can be accomplished.

## 6. Conclusions   

In this work, we have presented a general approach to model the internal strain and stress fields of arbitrarily shaped, toroidally bent crystal wafers and how they can be utilized to predict the diffraction properties of the wafer. Isotropic and anisotropic analytical solutions were derived for circular and rectangular wafers and their properties were discussed in detail, focusing on the special case of spherical bending. Comparisons to the available experimental data show that the models can make quantitatively accurate predictions. An open source implementation of the method was discussed and provided.

## 7. Related literature   

The following references are cited in the supporting information: Amenzade (1979 ▸); Lide (2001 ▸).

## Supplementary Material

Supporting information, equations and figures. DOI: 10.1107/S2052252520014165/hf5943sup1.pdf

## Footnotes

1 However, it should be noted the lack of photon flux in these CBCA instruments is related to the used Rowland circle geometry and does not necessarely affect CBCA instruments of different geometry (*e.g.* von Hámos geometry) with modern position-sensitive detectors like those presented in the work by Németh *et al.* (2016 ▸).

2 In the Voigt notation, a pair of indices *ij* is replaced with a single index *m* as follows: , , , ,  and . The compliance matrix *S* in the Voigt notation is given in terms of the compliance tensor *s* so that , where *ij* and *kl* are any pairs of indices corresponding to *m* and *n*, respectively, and δ is the Kronecker delta.

3 For higher order expansions, additional linear relations between *C_k_* are added to the system to ensure that *f*_c_ = 0 for all *x* and *y*.

4 The cut SBCAs in the X-ray Raman scattering spectrometer at beamline ID20 at ESRF are optimized in this manner (Huotari *et al.*, 2017 ▸).

## Appendix A Stretching energy minimization for an anisotropic rectangular wafer

The stretching energy  under the toroidal bending constraint is minimized in the anisotropic rectangular case by solving the expansion coefficients *C_k_* of the stress function χ from the linear system given by equation (19). The system is best solved numerically since, even though an analytical solution exists, it is too complicated to be practical. However, the analytical solution simplifies the problem as it turns out that the coefficients *C*_30_ = *C*_03_ = *C*_21_ = *C*_12_ = 0. In addition, the Lagrange multiplier for the integrated contact force λ_2_ = 0 which allows us to omit that constraint from the energy minimization. Thus we can reduce the number of unknowns to be solved from 14 down to 9. We now write the ansatz for χ in the following form:

where the numerical prefactors are chosen to simplify the form of the linear system. Substituting the ansatz into equation (14), we find the transverse stress tensor components to be

The toroidal minimization constraint [equation (16)] becomes

If we rewrite the Lagrange multiplier , we may reformulate the linear system as a matrix equation λ**C** = **b** in terms of **C** where

and

with

## References

[bb1] Ablett, J. M., Prieur, D., Céolin, D., Lassalle-Kaiser, B., Lebert, B., Sauvage, M., Moreno, T., Bac, S., BaléDent, V., Ovono, A., Morand, M., Gélebart, F., Shukla, A. & Rueff, J.-P. (2019). *J. Synchrotron Rad.* **26**, 263–271. 10.1107/S160057751801559X 30655494

[bb2] Aglitskiy, Y., Lehecka, T., Obenschain, S., Bodner, S., Pawley, C., Gerber, K., Sethian, J., Brown, C. M., Seely, J., Feldman, U. & Holland, G. (1998). *Appl. Opt.* **37**, 5253. 10.1364/ao.37.005253 18286004

[bb101] Amenzade, Y. A. (1979). *Theory of elasticity*. Moscow: Mir Publishers. (English translation)

[bb3] Anklamm, L., Schlesiger, C., Malzer, W., Grötzsch, D., Neitzel, M. & Kanngießer, B. (2014). *Rev. Sci. Instrum.* **85**, 053110. 10.1063/1.4875986 24880356

[bb4] Barrett, R., Härtwig, J., Morawe, C., Rommeveaux, A. & Snigirev, A. (2010). *Synchrotron Radiat. News*, **23**, 36–42.

[bb5] Bès, R., Ahopelto, T., Honkanen, A.-P., Huotari, S., Leinders, G., Pakarinen, J. & Kvashnina, K. (2018). *J. Nucl. Mater.* **507**, 50–53.

[bb6] Blasdell, R. C. & Macrander, A. T. (1995). *Rev. Sci. Instrum.* **66**, 2075–2077.

[bb7] Brown, P. N., Byrne, G. D. & Hindmarsh, A. C. (1989). *SIAM J. Sci. Stat. Comput.* **10**, 1038–1051.

[bb8] Cai, Y. Q. (2004). *AIP Conf. Proc.* **705**, 340–343.

[bb9] Cauchois, Y. (1932). *J. Phys. Radium*, **3**, 320–336.

[bb10] Chukhovskii, F. N., Chang, W. Z. & Förster, E. (1994). *J. Appl. Cryst.* **27**, 971–979.

[bb11] Davodi, F., Mühlhausen, E., Settipani, D., Rautama, E.-L., Honkanen, A.-P., Huotari, S., Marzun, G., Taskinen, P. & Kallio, T. (2019). *J. Colloid Interface Sci.* **556**, 180–192. 10.1016/j.jcis.2019.08.056 31445447

[bb12] Duan, P., Gu, S., Cao, H., Li, J. & Huang, Y. (2016). *X-ray Spectrom.* **46**, 12–18.

[bb13] DuMond, J. W. M. & Kirkpatrick, H. A. (1930). *Rev. Sci. Instrum.* **1**, 88–105.

[bb14] Erola, E., Eteläniemi, V., Suortti, P., Pattison, P. & Thomlinson, W. (1990). *J. Appl. Cryst.* **23**, 35–42.

[bb15] Faenov, A. Y., Pikuz, S. A., Erko, A. I., Bryunetkin, B. A., Dyakin, V. M., Ivanenkov, G. V., Mingaleev, A. R., Pikuz, T. A., Romanova, V. M. & Shelkovenko, T. A. (1994). *Phys. Scr.* **50**, 333–338.

[bb16] Fister, T. T., Seidler, G. T., Wharton, L., Battle, A. R., Ellis, T. B., Cross, J. O., Macrander, A. T., Elam, W. T., Tyson, T. A. & Qian, Q. (2006). *Rev. Sci. Instrum.* **77**, 063901.

[bb17] Gronkowski, J. (1991). *Phys. Rep.* **206**, 1–41.

[bb18] Holden, W. M., Hoidn, O. R., Ditter, A. S., Seidler, G. T., Kas, J., Stein, J. L., Cossairt, B. M., Kozimor, S. A., Guo, J., Ye, Y., Marcus, M. A. & Fakra, S. (2017). *Rev. Sci. Instrum.* **88**, 073904. 10.1063/1.4994739 28764488

[bb19] Honkanen, A.-P., Monaco, G. & Huotari, S. (2016). *J. Appl. Cryst.* **49**, 1284–1289.

[bb20] Honkanen, A.-P., Ollikkala, S., Ahopelto, T., Kallio, A.-J., Blomberg, M. & Huotari, S. (2019). *Rev. Sci. Instrum.* **90**, 033107. 10.1063/1.5084049 30927829

[bb21] Honkanen, A.-P., Verbeni, R., Simonelli, L., Moretti Sala, M., Al-Zein, A., Krisch, M., Monaco, G. & Huotari, S. (2014*a*). *J. Synchrotron Rad.* **21**, 762–767. 10.1107/S1600577514011163 PMC4861880 24971972

[bb22] Honkanen, A.-P., Verbeni, R., Simonelli, L., Moretti Sala, M., Monaco, G. & Huotari, S. (2014*b*). *J. Synchrotron Rad.* **21**, 104–110. 10.1107/S160057751302242X 24365923

[bb23] Hosoda, M., Ueda, K., Nagano, M., Zettsu, N., Shimada, S., Taniguchi, K. & Yamamura, K. (2010). *Key Eng. Mater.* **447–448**, 213–217.

[bb24] Huotari, S., Pylkkänen, T., Verbeni, R., Monaco, G. & Hämäläinen, K. (2011). *Nat. Mater.* **10**, 489–493. 10.1038/nmat3031 21623376

[bb25] Huotari, S., Sahle, C. J., Henriquet, C., Al-Zein, A., Martel, K., Simonelli, L., Verbeni, R., Gonzalez, H., Lagier, M.-C., Ponchut, C., Moretti Sala, M., Krisch, M. & Monaco, G. (2017). *J. Synchrotron Rad.* **24**, 521–530. 10.1107/S1600577516020579 28244449

[bb26] Ishii, K., Jarrige, I., Yoshida, M., Ikeuchi, K., Inami, T., Murakami, Y. & Mizuki, J. (2013). *J. Electron Spectrosc. Relat. Phenom.* **188**, 127–132.

[bb27] Jahrman, E. P., Holden, W. M., Ditter, A. S., Kozimor, S. A., Kihara, S. L. & Seidler, G. T. (2019*a*). *Rev. Sci. Instrum.* **90**, 013106. 10.1063/1.5057231 30709184

[bb28] Jahrman, E. P., Holden, W. M., Ditter, A. S., Mortensen, D. R., Seidler, G. T., Fister, T. T., Kozimor, S. A., Piper, L. F. J., Rana, J., Hyatt, N. C. & Stennett, M. C. (2019*b*). *Rev. Sci. Instrum.* **90**, 024106. 10.1063/1.5049383 30831699

[bb29] Jahrman, E. P., Pellerin, L. A., Ditter, A. S., Bradshaw, L. R., Fister, T. T., Polzin, B. J., Trask, S. E., Dunlop, A. R. & Seidler, G. T. (2019*c*). *J. Electrochem. Soc.* **166**, A2549–A2555.

[bb30] Jahrman, E. P., Seidler, G. T. & Sieber, J. R. (2018). *Anal. Chem.* **90**, 6587–6593. 10.1021/acs.analchem.8b00302 PMC6089071 29762013

[bb31] Johann, H. H. (1931). *Z. Phys.* **69**, 185–206.

[bb32] Johansson, T. (1932). *Sci. Nat.* **20**, 758–759.

[bb33] Jones, E., Oliphant, T., Peterson, P. *et al.* (2001). *SciPy: Open source scientific tools for Python*, http://www.scipy.org/.

[bb34] Kleymenov, E., van Bokhoven, J. A., David, C., Glatzel, P., Janousch, M., Alonso-Mori, R., Studer, M., Willimann, M., Bergamaschi, A., Henrich, B. & Nachtegaal, M. (2011). *Rev. Sci. Instrum.* **82**, 065107. 10.1063/1.3600452 21721730

[bb35] Knapp, P. F., Pikuz, S. A., Shelkovenko, T. A., Hammer, D. A. & Hansen, S. B. (2011). *Rev. Sci. Instrum.* **82**, 063501. 10.1063/1.3592582 21721685

[bb36] Kuai, L., Kan, E., Cao, W., Huttula, M., Ollikkala, S., Ahopelto, T., Honkanen, A.-P., Huotari, S., Wang, W. & Geng, B. (2018). *Nano Energy*, **43**, 81–90.

[bb37] Kvashnina, K. O. & Scheinost, A. C. (2016). *J. Synchrotron Rad.* **23**, 836–841. 10.1107/S1600577516004483 27140166

[bb38] Landau, L. D., Pitaevskii, L. P., Kosevich, A. M. & Lifshitz, E. M. (1986). *Theory Of Elasticity*, 3rd ed. Oxford: Butterworth–Heinemann.

[bb102] Lide, D. L. (2001). *CRC Handbook of Chemistry and Physics*, 82nd ed. Boca Raton: CRC Press

[bb39] Lusa, M., Help, H., Honkanen, A.-P., Knuutinen, J., Parkkonen, J., Kalasová, D. & Bomberg, M. (2019). *Environ. Res.* **177**, 108642. 10.1016/j.envres.2019.108642 31430668

[bb40] Lutz, C. & Fittschen, U. E. A. (2020). *Powder Diffr.* 1–5.

[bb41] Michell, J. H. (1899). *Proc. London Math. Soc.* **s1-31**, 100–124.

[bb42] Moretti Sala, M., Martel, K., Henriquet, C., Al Zein, A., Simonelli, L., Sahle, C., Gonzalez, H., Lagier, M.-C., Ponchut, C., Huotari, S., Verbeni, R., Krisch, M. & Monaco, G. (2018). *J. Synchrotron Rad.* **25**, 580–591. 10.1107/S1600577518001200 29488940

[bb43] Mortensen, D. R., Seidler, G. T., Kas, J. J., Govind, N., Schwartz, C. P., Pemmaraju, S. & Prendergast, D. G. (2017). *Phys. Rev. B*, **96**, 125136.

[bb44] Mottram, L., Cafferkey, S., Mason, A., Oulton, T., Kuan Sun, S., Bailey, D., Stennett, M. & Hyatt, N. (2020*a*). *J. Geosci.* **65**, 27–35.

[bb45] Mottram, L., Wilkins, M. D., Blackburn, L., Oulton, T., Stennett, M., Sun, S., Corkhill, C. & Hyatt, N. (2020*b*). *MRS Adv.* **5**, 27–35.

[bb46] Moya-Cancino, J. G., Honkanen, A.-P., van der Eerden, A. M. J., Schaink, H., Folkertsma, L., Ghiasi, M., Longo, A., de Groot, F. M. F., Meirer, F., Huotari, S. & Weckhuysen, B. M. (2019*a*). *ChemCatChem*, **11**, 1039–1044. 10.1002/cctc.201801822 PMC6471006 31007776

[bb47] Moya–Cancino, J. G., Honkanen, A.-P., van der Eerden, A. M. J., Schaink, H., Folkertsma, L., Ghiasi, M., Longo, A., Meirer, F., de Groot, F. M. F., Huotari, S. & Weckhuysen, B. M. (2019*b*). *ChemCatChem*, **11**, 3042–3045. 10.1002/cctc.201801822 PMC6471006 31007776

[bb48] Németh, Z., Szlachetko, J., Bajnóczi, G. & Vankó, G. (2016). *Rev. Sci. Instrum.* **87**, 103105. 10.1063/1.4964098 27802722

[bb49] Onuki, T., Takagi, N., Shimizu, J., Ojima, H. & Zhou, L. B. (2011). *Adv. Mater. Res.* **325**, 672–677.

[bb51] Rio, M. S., Alianelli, L., Faenov, A. Y. & Pikuz, T. (2004). *Phys. Scr.* **69**, 297–302.

[bb50] Rovezzi, M., Lapras, C., Manceau, A., Glatzel, P. & Verbeni, R. (2017). *Rev. Sci. Instrum.* **88**, 013108. 10.1063/1.4974100 28147645

[bb52] Sánchez del Río, M. & Dejus, R. J. (2011). *Proc. SPIE*, **8141**, 814115.

[bb53] Schoonjans, T., Brunetti, A., Golosio, B., Sanchez del Rio, M., Solé, V. A., Ferrero, C. & Vincze, L. (2011). *At. Spectrosc.* **66**, 776–784.

[bb54] Seidler, G. T., Mortensen, D. R., Remesnik, A. J., Pacold, J. I., Ball, N. A., Barry, N., Styczinski, M. & Hoidn, O. R. (2014). *Rev. Sci. Instrum.* **85**, 113906. 10.1063/1.4901599 25430123

[bb55] Shvyd’ko, Y., Hill, J., Burns, C., Coburn, D., Brajuskovic, B., Casa, D., Goetze, K., Gog, T., Khachatryan, R., Kim, J.-H., Kodituwakku, C., Ramanathan, M., Roberts, T., Said, A., Sinn, H., Shu, D., Stoupin, S., Upton, M., Wieczorek, M. & Yavas, H. (2013). *J. Electron Spectrosc. Relat. Phenom.* **188**, 140–149.

[bb56] Sinars, D. B., Cuneo, M. E., Bennett, G. R., Wenger, D. F., Ruggles, L. E., Vargas, M. F., Porter, J. L., Adams, R. G., Johnson, D. W., Keller, K. L., Rambo, P. K., Rovang, D. C., Seamen, H., Simpson, W. W., Smith, I. C. & Speas, S. C. (2003). *Rev. Sci. Instrum.* **74**, 2202–2205.

[bb57] Sokaras, D., Nordlund, D., Weng, T.-C., Mori, R. A., Velikov, P., Wenger, D., Garachtchenko, A., George, M., Borzenets, V., Johnson, B., Qian, Q., Rabedeau, T. & Bergmann, U. (2012). *Rev. Sci. Instrum.* **83**, 043112. 10.1063/1.4704458 PMC4108631 22559520

[bb58] Sun, Y., Xia, Y., Kuai, L., Sun, H., Cao, W., Huttula, M., Honkanen, A.-P., Viljanen, M., Huotari, S. & Geng, B. (2019). *ChemSusChem*, **12**, 2564–2569. 10.1002/cssc.201900831 31017344

[bb59] Suortti, P. & Schulze, C. (1995). *J. Synchrotron Rad.* **2**, 6–12. 10.1107/S0909049594009763 16714780

[bb60] Takagi, S. (1962). *Acta Cryst.* **15**, 1311–1312.

[bb61] Takagi, S. (1969). *J. Phys. Soc. Jpn*, **26**, 1239–1253.

[bb62] Taupin, D. (1964). *Bull. Soc. Fr. Minéral. Crist.* **87**, 469–511.

[bb63] Thiess, H., Lasser, H. & Siewert, F. (2010). *Nucl. Instrum. Methods Phys. Res. A*, **616**, 157–161.

[bb64] Verbeni, R., Kocsis, M., Huotari, S., Krisch, M., Monaco, G., Sette, F. & Vanko, G. (2005). *J. Phys. Chem. Solids*, **66**, 2299–2305.

[bb65] Verbeni, R., Pylkkänen, T., Huotari, S., Simonelli, L., Vankó, G., Martel, K., Henriquet, C. & Monaco, G. (2009). *J. Synchrotron Rad.* **16**, 469–476. 10.1107/S090904950901886X 19535859

[bb66] von Hámos, L. (1932). *Naturwissenschaften*, **20**, 705–706.

[bb67] Wang, W., Kuai, L., Cao, W., Huttula, M., Ollikkala, S., Ahopelto, T., Honkanen, A.-P., Huotari, S., Yu, M. & Geng, B. (2017). *Angew. Chem. Int. Ed.* **56**, 14977–14981. 10.1002/anie.201708765 29024224

[bb68] Welter, E., Machek, P., Dräger, G., Brüggmann, U. & Fröba, M. (2005). *J. Synchrotron Rad.* **12**, 448–454. 10.1107/S0909049505007843 15968121

[bb69] White, J. E. (1950). *J. Appl. Phys.* **21**, 855–859.

[bb70] Yamaoka, H., Mochizuki, T., Sakurai, Y. & Kawata, H. (1998). *J. Synchrotron Rad.* **5**, 699–701. 10.1107/S0909049597018645 15263624

[bb71] Yumoto, H., Mimura, H., Kimura, T., Handa, S., Matsuyama, S., Sano, Y. & Yamauchi, K. (2008). *Surf. Interface Anal.* **40**, 1023–1027.

