# Supplementary material for: General method to calculate the elastic deformation and X-ray diffraction properties of bent crystal wafers
Source: IUCrJ. 2021 Jan 1;8(Pt 1):102–15. doi: 10.1107/S2052252520014165 (PMC7793001; doi:10.1107/S2052252520014165)
Supplement: Supplementary file 1 [file m-08-00102-sup1.pdf]

IUCrJ

Volume 7 (2020)

Supporting information for article:

General method to calculate the elastic deformation and X-ray diffraction properties of bent crystal wafers

Ari-Pekka Honkanen and Simo Huotari

Supplementary material to General method to calculate the elastic deformation and X-ray diffraction properties of bent crystal wafers

Ari-Pekka Honkanen[1]* and Simo Huotari[2]

University of Helsinki, Department of Physics, PO Box 64, FI-00014 Helsinki, Finland. E-mail: ari-pekka.honkanen@helsinki.fi

This supplementary material amends the main text by providing the mathematical details for derived theoretical results.For a quick review of underlying assumptions in the model and its special case applications, see Appendix A.

1. General Theory

1.1. Solving the deformation field of arbitrarily shaped toroidally bent crystal wafer

Consider an arbitrarily shaped thin anisotropic crystal wafer of thickness d presented in Figure 1. We choose a Cartesian coordinate system (x, y, z) so that the origin of the system coincides with the midplane of the wafer with the z-direction parallel to the normal of the crystal surface. The displacement vector field $\boldsymbol{\epsilon}$ due to two orthogonal

[1] https://orcid.org/0000-0002-6822-3062
[2] https://orcid.org/0000-0003-4506-8722

torques acting on the wafer about the x- and y-axes is (Chukhovskii *et al.*, 1994)

$$\epsilon_x = (S_{11}\mu_x + S_{12}\mu_y)xz + (S_{51}\mu_x + S_{52}\mu_y)\frac{z^2}{2} + (S_{61}\mu_x + S_{62}\mu_y)\frac{yz}{2} \quad (1)$$

$$\epsilon_y = (S_{21}\mu_x + S_{22}\mu_y)yz + (S_{41}\mu_x + S_{42}\mu_y)\frac{z^2}{2} + (S_{61}\mu_x + S_{62}\mu_y)\frac{xz}{2} \quad (2)$$

$$\epsilon_z = -(S_{11}\mu_x + S_{12}\mu_y)\frac{x^2}{2} - (S_{21}\mu_x + S_{22}\mu_y)\frac{y^2}{2} - (S_{61}\mu_x + S_{62}\mu_y)\frac{xy}{2} + (S_{31}\mu_x + S_{32}\mu_y)\frac{z^2}{2} \quad (3)$$

where S_{ij} are components of the compliance matrix as used in the Voigt notation[1]. For the subsequent mathematical convenience the torques μ_x and μ_y are scaled so that they are presented in units of torque per unit length per the area moment of inertia. The subscript of the scaled torques refers to direction along which the torque primarily bends the crystal, not their axes (μ_x acts about the y-axis and μ_y about the x-axis). The displacement vector field (1)–(3) applies for the case where the out-of-plane deformation is sufficiently small to not cause significant stretching in the in-plane directions and is thus called a *pure bending solution* (Chukhovskii *et al.*, 1994).

When transverse (x, y) dimensions of the wafer are small compared to the toroidal bending radii R_1 and R_2, the vertical (z) deflection ζ of the wafer can be approximated by a paraboloidal surface

$$\zeta(x,y) = \left(\frac{\cos^2\phi}{R_1} + \frac{\sin^2\phi}{R_2}\right)\frac{x^2}{2} - \sin 2\phi\left(\frac{1}{R_1} - \frac{1}{R_2}\right)\frac{xy}{2} + \left(\frac{\sin^2\phi}{R_1} + \frac{\cos^2\phi}{R_2}\right)\frac{y^2}{2} \quad (4)$$

where ϕ is the in-plane inclination of the bending radii with the Cartesian coordinate axes x and y (clockwise-positive). Comparing Eq. (4) to Eq. (3), we see that under the action of μ_x and μ_y the vertical deflection of the midplane ($z = 0$) wafer adopts a paraboloidal shape approximating the toroidal bending (see Fig. 2).

In principle the problem could be inverted by fixing ζ by choosing R_1, R_2, and ϕ and solving the torques μ_x and μ_y by equating Eqs. (3) and (4). When μ_x and μ_y

[1] In the Voigt notation, a pair of indices ij is replaced with a single index m as follows: $11 \rightarrow 1$; $22 \rightarrow 2$; $33 \rightarrow 3$; $23, 32 \rightarrow 4$; $13, 31 \rightarrow 5$ and $12, 21 \rightarrow 6$. The compliance matrix S in the Voigt notation is given in terms of the compliance tensor s so that $S_{mn} = (2 - \delta_{ij})(2 - \delta_{kl})s_{ijkl}$, where ij and kl are any pairs of indices corresponding to m and n, respectively, and δ is the Kronecker delta.

are known, the pure bending solution is completely known and thus can be used in diffraction calculations. However, since there are two torques and three parameters that define the shape and orientation of the deflection in z, the only two of R_1, R_2, and ϕ can be chosen freely and the third one is determined by S_{ij}. For example, in the case of spherical bending $R_1 = R_2$ which means that the xy-term in Eq. (4) should vanish. However, this requires that $S_{61}\mu_x + S_{62}\mu_y = 0$ in Eq. (3), which is not true in the general case.

The torques acting on the wafer *in natura* are imposed by the contact to the substrate onto which the wafer is forced and can choose their axes of action freely to conform the shape of the wafer to that of the substrate. Therefore we introduce an additional degree of freedom to the pure bending solution by allowing the joint rotation of μ_x and μ_y in plane, which is sufficient to allow R_1, R_2, and ϕ to be chosen freely. The solution (1)–(3) assumes that μ_x and μ_y act about the fixed coordinate axes x and y but mathematically the same effect can be achieved by introducing the additional in-plane rotational degree of freedom α to S_{ij} and ϕ instead. Combining Eqs. (3) and (4) with well-known trigonometric identities, we thus need to find the torques μ_x and μ_y and the in-plane rotation angle α so that the following equations are fulfilled simultaneously:

$$S'_{11}\mu_x + S'_{12}\mu_y = -\frac{1}{2}\left(\frac{1}{R_1} + \frac{1}{R_2}\right) - \frac{1}{2}\left(\frac{1}{R_1} - \frac{1}{R_2}\right)\cos 2\phi' \qquad (5)$$

$$S'_{21}\mu_x + S'_{22}\mu_y = -\frac{1}{2}\left(\frac{1}{R_1} + \frac{1}{R_2}\right) + \frac{1}{2}\left(\frac{1}{R_1} - \frac{1}{R_2}\right)\cos 2\phi' \qquad (6)$$

$$S'_{61}\mu_x + S'_{62}\mu_y = \left(\frac{1}{R_1} - \frac{1}{R_2}\right)\sin 2\phi' \qquad (7)$$

where S'_{ij} are the components of the rotated compliance matrix and $\phi' = \phi + \alpha$.

To simplify the problem, we set $\phi = 0$ *i.e.* we choose the orientation of the toroidal surface so that the bending radii R_1 and R_2 are oriented parallel to the x- and y-axes, respectively. This can be done without a loss of generality, since S_{ij} can always be

rotated in-plane so that relative orientation of the crystal directions to the toroidal surface is kept fixed. Now solving μ_x and μ_y from Eqs. (5) and Eqs. (6) yields

$$\mu_x = \frac{(S'_{12} - S'_{22})(R_1 + R_2) + (S'_{12} + S'_{22})(R_1 - R_2)\cos 2\alpha}{2(S'_{11}S'_{22} - S'_{12}S'_{12})R_1 R_2} \tag{8}$$

$$\mu_y = \frac{(S'_{12} - S'_{11})(R_1 + R_2) - (S'_{12} + S'_{11})(R_1 - R_2)\cos 2\alpha}{2(S'_{11}S'_{22} - S'_{12}S'_{12})R_1 R_2} \tag{9}$$

where $S'_{12} = S'_{21}$ based on the symmetry of S was used. Now, substituting the obtained torques to Equation (7) leads to the condition

$$\begin{aligned}\Big[2(S'_{12}S'_{12} - S'_{11}S'_{22})\sin 2\alpha + \left[S'_{61}(S'_{22} + S'_{12}) - S'_{62}(S'_{11} + S'_{12})\right]\cos 2\alpha\Big](R_1 - R_2)\\ = \left[S'_{61}(S'_{22} - S'_{12}) + S'_{62}(S'_{11} - S'_{12})\right](R_1 + R_2)\end{aligned} \tag{10}$$

The in-plane rotation angle α fulfilling the condition (10) can be solved by performing a rotation to the compliance tensor s according to

$$s'_{ijkl} = \sum_{p,q,r,s} Q_{ip}Q_{jq}Q_{kr}Q_{ls}s_{pqrs} \tag{11}$$

where Q is the rotation matrix corresponding to the counterclockwise rotation by α about z-axis that is given by

$$Q = \begin{bmatrix} \cos\alpha & -\sin\alpha & 0 \\ \sin\alpha & \cos\alpha & 0 \\ 0 & 0 & 1 \end{bmatrix}. \tag{12}$$

Constructing the relevant components of the rotated compliance matrix S' from s'_{ijkl} allows us to write the Eq. (10) in terms of S:

$$(A_\alpha \sin 2\alpha + B_\alpha \cos 2\alpha)(R_1 - R_2) = (C_\alpha \sin 2\alpha + D_\alpha \cos 2\alpha)(R_1 + R_2) \tag{13}$$

where

$$A_\alpha \equiv S_{66}(S_{11} + S_{22} + 2S_{12}) - (S_{61} + S_{62})^2 \tag{14}$$

$$B_\alpha \equiv 2\left[S_{62}(S_{12} + S_{11}) - S_{61}(S_{12} + S_{22})\right] \tag{15}$$

$$C_\alpha \equiv S_{66}(S_{22} - S_{11}) + S_{61}^2 - S_{62}^2 \tag{16}$$

$$D_\alpha \equiv 2\left[S_{62}(S_{12} - S_{11}) + S_{61}(S_{12} - S_{22})\right]. \tag{17}$$

Solving for α, we find

$$\alpha = \frac{1}{2}\,\mathrm{atan}\left[\frac{D_\alpha(R_1+R_2) - B_\alpha(R_1-R_2)}{A_\alpha(R_1-R_2) - C_\alpha(R_1+R_2)}\right] + \frac{\pi n}{2}, \tag{18}$$

where $n \in \mathbb{Z}$. The derivation of the obtained expression is based on the assumption that at least either of S'_{61} or S'_{62} is non-zero. By examining the rotated components in detail, we find that this assumption fails if the following conditions are simultaneously true: $S_{61} = S_{62} = 0$, $S_{11} = S_{22}$, and $S_{11} + S_{22} - 2S_{12} - S_{66} = 0$. These are fulfilled by elastically isotropic materials and specifically oriented crystals *e.g.* cubic systems with [111] or its equivalent directions parallel to z-axis. In such a case, Eq (7) reduces to $\sin 2\alpha = 0$ which leads to $\alpha = \pi n/2$. Since any valid α suits the purpose, we may choose $n = 0$ for simplicity in both cases.

Since the crystal does not rotate physically, we need to compensate the applied in-plane rotation by rotating the coordinate system as well. This means the rotation of the displacement vector $\boldsymbol{\epsilon}' = Q^T\boldsymbol{\epsilon}$ and replacement of the scalar coordinates by $x \to x\cos\alpha + y\sin\alpha$ and $y \to y\cos\alpha - x\sin\alpha$. Note that we do not apply the rotation to the products $S'_{ij}\mu_k$ as these terms behave as constant scalars. Thus the components of the displacement vector field in the pure bending solution [Eqs. (1)–(3)] for spherical bending become

$$\epsilon'_x = -\frac{xz}{R_1} + \left[(S'_{51}\mu_x + S'_{52}\mu_y)\cos\alpha - (S'_{41}\mu_x + S'_{42}\mu_y)\sin\alpha\right]\frac{z^2}{2} \tag{19}$$

$$\epsilon'_y = -\frac{yz}{R_2} + \left[(S'_{51}\mu_x + S'_{52}\mu_y)\sin\alpha + (S'_{41}\mu_x + S'_{42}\mu_y)\cos\alpha\right]\frac{z^2}{2} \tag{20}$$

$$\epsilon'_z = \frac{x^2}{2R_1} + \frac{y^2}{2R_2} + (S'_{31}\mu_x + S'_{32}\mu_y)\frac{z^2}{2} \tag{21}$$

where the S'_{ij}, α, μ_x and μ_y are best calculated numerically using Eqs. (8), (9), (11) and (18). Note that $\epsilon'_z(x, y, 0)$ corresponds to the vertical deflection

$$\zeta(x, y) = \frac{x^2}{2R_1} + \frac{y^2}{2R_2} \tag{22}$$

corresponding to $\phi = 0$. Assuming the diffraction to take place in the xz-plane, the partial derivatives needed for the diffraction calculations are

$$\frac{\partial \epsilon'_x}{\partial x} = -\frac{z}{R_1} \qquad \frac{\partial \epsilon'_z}{\partial x} = \frac{x}{R_1} \qquad \frac{\partial \epsilon'_z}{\partial z} = (S'_{31}\mu_x + S'_{32}\mu_y)z$$
$$\frac{\partial \epsilon'_x}{\partial z} = -\frac{x}{R_1} + \left[(S'_{51}\mu_x + S'_{52}\mu_y)\cos\alpha - (S'_{41}\mu_x + S'_{42}\mu_y)\sin\alpha\right] z \tag{23}$$

For the isotropic case[2], the torques given by Eqs. (8) and (9) reduce to

$$\mu_x = -\frac{E}{1-\nu^2}\left(\frac{1}{R_1} + \frac{\nu}{R_2}\right) \qquad \mu_y = -\frac{E}{1-\nu^2}\left(\frac{\nu}{R_1} + \frac{1}{R_2}\right) \tag{24}$$

and thus the partial derivatives of $\boldsymbol{\epsilon}'$ become

$$\frac{\partial \epsilon'_x}{\partial x} = -\frac{z}{R_1} \qquad \frac{\partial \epsilon'_x}{\partial z} = -\frac{x}{R_1} \qquad \frac{\partial \epsilon'_z}{\partial x} = \frac{x}{R_1} \qquad \frac{\partial \epsilon'_z}{\partial z} = \frac{\nu}{1-\nu}\left(\frac{1}{R_1} + \frac{1}{R_2}\right) z \tag{25}$$

The partial derivatives (23) can be used as a deformation term in the Takagi-Taupin equations (Takagi, 1962; Taupin, 1964; Takagi, 1969) to estimate the intensity of X-rays diffracted by the crystal as a function of incident wavelength or incidence angle *aka* the diffraction curve of a bent crystal due to a particular set of crystalline planes of toroidally bent crystals. However, the pure bending solution Eq. (23) alone is inadequate as it fails to explain the diffraction curves of SBCAs with a large surface area (Verbeni *et al.*, 2009; Honkanen *et al.*, 2014; Rovezzi *et al.*, 2017). This is because, in addition to pure bending strain, the flat crystal wafer is also stretched and compressed in the transverse (in-plane) directions in order to fit on a spherical surface. These deformations affect the d-spacing *i.e.* the separation of the diffracting Bragg planes due to non-zero Poisson ratio and thus the resolution function of the SBCA. In the scope of linear elasticity, the total strain tensor is $\tilde{\epsilon}_{ij} = \epsilon'_{ij} + u_{ij}$, where in addition to the pure bending strain ϵ'_{ij} we include the *stretching component* u_{ij}. In what follows, a theoretical foundation for solving u_{ij} is presented.

[2] The non-zero components are $S'_{11} = S'_{22} = S'_{33} = 1/E$, $S'_{12} = S'_{21} = S'_{13} = S'_{31} = S'_{23} = S'_{32} = -\nu/E$, and $S'_{44} = S'_{55} = S'_{66} = 2(1+\nu)/E$

According to Hooke's law, the components of the strain tensor due to stretching u_{ij} are connected to the stretching stress tensor σ_{ij} *via*

$$u_{ij} = \sum_{k,l} s_{ijkl}\sigma_{kl} \tag{26}$$

where s_{ijkl} is the compliance tensor. Using the Voigt notation to convert the fourth-order compliance tensor to a matrix, Equation (26) gives the following relations

$$u_{xx} = S_{11}\sigma_{xx} + S_{12}\sigma_{yy} + S_{16}\sigma_{xy} \tag{27}$$

$$u_{yy} = S_{21}\sigma_{xx} + S_{22}\sigma_{yy} + S_{26}\sigma_{xy} \tag{28}$$

$$u_{xy} = \frac{1}{2}\left(S_{61}\sigma_{xx} + S_{62}\sigma_{yy} + S_{66}\sigma_{xy}\right). \tag{29}$$

In Eqs.(27)–(29) we have assumed $\sigma_{xz} = \sigma_{yz} = \sigma_{zz} = 0$, since the external forces required to bend a thin plate are small compared to the internal stresses and can thus be omitted at this stage. For an isotropic crystal, the relations simplify to

$$u_{xx} = \frac{\sigma_{xx} - \nu\sigma_{yy}}{E} \qquad u_{yy} = \frac{\sigma_{yy} - \nu\sigma_{xx}}{E} \qquad u_{xy} = \frac{1+\nu}{E}\sigma_{xy}, \tag{30}$$

where E is Young's modulus and ν is Poisson's ratio.

The transverse components of u_{ij} are given by Eq. (14.1) in (Landau *et al.*, 1986)[p. 51] as follows

$$u_{ij} = \frac{1}{2}\left(\frac{\partial u_i}{\partial x_j} + \frac{\partial u_j}{\partial x_i}\right) + \frac{1}{2}\frac{\partial \zeta}{\partial x_i}\frac{\partial \zeta}{\partial x_j}, \tag{31}$$

where u_i are the components of the displacement vector due to stretching and ζ is the vertical displacement of the wafer. The possible values of i and j are now restricted to the in-plane directions x and y. The strain tensor must fulfil the equilibrium condition $\sum_k \partial\sigma_{ik}/\partial x_k = 0$ which is ascertained if we write the σ_{ij} as a function of $\chi = \chi(x, y)$, also known as the Airy stress function, so that

$$\sigma_{xx} = \frac{\partial^2\chi}{\partial y^2}, \qquad \sigma_{xy} = -\frac{\partial^2\chi}{\partial x\partial y}, \qquad \sigma_{yy} = \frac{\partial^2\chi}{\partial x^2}. \tag{32}$$

We are now set to find u_{ij} which we will achieve by minimising the relevant thermodynamic potential, that is, the Helmholtz energy (Amenzade, 1979)[pp. 72]. The Helmholtz energy density f for the mechanical deformation is given by

$$f = \frac{1}{2}\sum_{k,l} \tilde{\epsilon}_{kl}\tilde{\sigma}_{kl} \tag{33}$$

where the total stress tensor $\tilde{\sigma}_{kl}$ is related to the total strain tensor $\tilde{\epsilon}_{kl}$ *via* Hooke's law. Generally we can divide $\tilde{\epsilon}_{kl}$ and $\tilde{\sigma}_{kl}$ to sums of two components $\tilde{\epsilon}_{kl} = (\epsilon_{kl})_1 + (\epsilon_{kl})_2$ and $\tilde{\sigma}_{kl} = (\sigma_{kl})_1 + (\sigma_{kl})_2$ where the respective components are connected by Hooke's law. Integrating over the volume of the wafer thus gives the Helmholtz energy

$$\mathcal{F} = \mathcal{F}_1 + \mathcal{F}_2 + \frac{1}{2}\int dV \sum_{k,l}\Big[(\epsilon_{kl})_1(\sigma_{kl})_2 + (\epsilon_{kl})_2(\sigma_{kl})_1\Big] \tag{34}$$

where

$$\mathcal{F}_i = \frac{1}{2}\int dV \sum_{k,l}(\epsilon_{kl})_i(\sigma_{kl})_i. \tag{35}$$

In the general case, the cross-term in Eq. (34) is non-zero. However, if we assume that

- $(\sigma_{kl})_i = 0$ when k or $l = z$
- $(\epsilon_{kl})_1$ (and thus $(\sigma_{kl})_1$) is proportional to z for all k, l
- $(\epsilon_{kl})_2$ (and thus $(\sigma_{kl})_2$) is independent of z for all k, l

then the cross-term vanishes and the total energy $\mathcal{F} = \mathcal{F}_1 + \mathcal{F}_2$. If the wafer is thin *i.e* its thickness is much smaller than its in-plane dimensions, then the external forces needed to deflect the wafer in z-direction are much smaller than the internal stresses and thus $(\sigma_{zl})_i = (\sigma_{kz})_i = 0$ for all k, l. We can also assume that strains and stresses due to in-plane stretching have very little z-dependence in a thin wafer. Finally, since in the absence of stretching component the deformation of the crystal is described by the pure bending solution, we know that the non-stretching component has to be at least approximately linear in z; any higher order terms can be considered negligible. We thus conclude that for a thin crystal the Helmholtz energy $\mathcal{F}$ can be written as sum of two separate components $\mathcal{F}_1$ and $\mathcal{F}_2$.

Since we already know that the pure bending solution is the answer in absence of stretching, we know that it minimizes $\mathcal{F}_1$ and thus we can focus solely on the other component $\mathcal{F}_2$. Dropping out the subscript for simplicity, the stretching energy can be written as

$$\mathcal{F} = \frac{d}{2}\int_\Omega d\Omega \sum_{k,l} u_{kl}\sigma_{kl} = \frac{d}{2}\int_\Omega d\Omega \,\Big(u_{xx}\sigma_{xx} + 2u_{xy}\sigma_{xy} + u_{yy}\sigma_{yy}\Big), \tag{36}$$

where the integration goes over the crystal surface Ω. Substituting Eqs.(27)–(29), we obtain

$$\mathcal{F} = \frac{d}{2}\int_\Omega d\Omega \,\Big(S_{11}\sigma_{xx}^2 + S_{22}\sigma_{yy}^2 + S_{66}\sigma_{xy}^2 + 2S_{12}\sigma_{xx}\sigma_{yy} + 2S_{16}\sigma_{xx}\sigma_{xy} + 2S_{26}\sigma_{yy}\sigma_{xy}\Big), \tag{37}$$

which in the isotropic case simplifies to

$$\mathcal{F} = \frac{d}{2E}\int_\Omega d\Omega \,\Big[\sigma_{xx}^2 + 2(1+\nu)\sigma_{xy}^2 + \sigma_{yy}^2 - 2\nu\sigma_{xx}\sigma_{yy}\Big]. \tag{38}$$

The deformation field can be now found by minimizing $\mathcal{F}$ in terms of χ, *i.e.,* we need to find χ so that the functional derivative $\delta\mathcal{F}/\delta\chi = 0$. While we could try to solve the problem using the Euler-Lagrange equations, we may utilize the fact that the dimensions of the crystals are small compared to the bending radii $R_{1,2}$. Therefore we may write the ansatz in powers of x/R_1 and y/R_2 and truncate the series after a few lowest-order terms. The $\mathcal{F}$ is then minimized in terms of the expansion coefficients C_k. Since $\mathcal{F}$ is quadratic in terms of χ and thus in terms of C_k, the problem of solving the Euler-Lagrange equations is thus reduced to a finite linear system $\partial\mathcal{F}/\partial C_k = 0$. Taking the partial derivatives of Eq. (37), we find

$$\begin{aligned}\partial_k\mathcal{F} = d\int_\Omega d\Omega \,\Big[&\left(S_{11}\partial_k\sigma_{xx} + S_{12}\partial_k\sigma_{yy} + S_{16}\partial_k\sigma_{xy}\right)\sigma_{xx}\\ &+ \left(S_{12}\partial_k\sigma_{xx} + S_{22}\partial_k\sigma_{yy} + S_{26}\partial_k\sigma_{xy}\right)\sigma_{yy}\\ &+ \left(S_{16}\partial_k\sigma_{xx} + S_{26}\partial_k\sigma_{yy} + S_{66}\partial_k\sigma_{xy}\right)\sigma_{xy}\Big]\end{aligned} \tag{39}$$

where a shorthand $\partial_k \equiv \partial/\partial C_k$ has been used. For the isotropic crystal the equations simplify to

$$\partial_k \mathcal{F} = \frac{d}{E}\int_\Omega d\Omega \, \Big[\left(\partial_k \sigma_{xx} - \nu \partial_k \sigma_{yy}\right) \sigma_{xx} + \left(\partial_k \sigma_{yy} - \nu \partial_k \sigma_{xx}\right) \sigma_{yy} + 2(1+\nu)(\partial_k \sigma_{xy})\sigma_{xy}\Big]. \tag{40}$$

In addition, we need to impose two constraints to the energy minimization to include the toroidal bending and the requirement that the integrated contact force at the wafer–substrate interface acting on the wafer vanishes. First, for the toroidal bending we need to find the relationship between χ and the vertical displacement ζ. As presented in Appendix B, by combining Eqs. (27)–(29), (31), and (32), we obtain the following partial differential equation

$$\mathcal{D}^4 \chi = \left(\frac{\partial^2 \zeta}{\partial x \partial y}\right)^2 - \frac{\partial^2 \zeta}{\partial x^2}\frac{\partial^2 \zeta}{\partial y^2}, \tag{41}$$

where

$$\mathcal{D}^4 \equiv S_{11}\frac{\partial^4}{\partial y^4} + (2S_{12} + S_{66})\frac{\partial^4}{\partial x^2 \partial y^2} + S_{22}\frac{\partial^4}{\partial x^4} - 2S_{16}\frac{\partial^4}{\partial x \partial y^3} - 2S_{26}\frac{\partial^4}{\partial x^3 \partial y}. \tag{42}$$

Substituting the toroidal displacement $\zeta(x,y) = x^2/2R_1 + y^2/2R_2$ into Eq. (41), we thus obtain

$$\mathcal{D}^4 \chi = -\frac{1}{R_1 R_2}, \tag{43}$$

which in the isotropic case simplifies to

$$\nabla^4 \chi = -\frac{E}{R_1 R_2}. \tag{44}$$

In addition, since contact force P per unit area, as given in Appendix C, between the wafer and substrate is the only external force acting on the wafer, we require that its integral over the surface has to vanish in order the wafer to stay stationary *i.e.*

$$P = -d\left(\frac{\sigma_{xx}}{R_1} + \frac{\sigma_{yy}}{R_2}\right). \tag{45}$$

Thus the integrated contact force F_c required to vanish over the wafer–substrate interface is

$$F_c = -d \int_\Omega d\Omega \, \left(\frac{\sigma_{xx}}{R_1} + \frac{\sigma_{yy}}{R_2} \right) = 0. \tag{46}$$

Limiting the expansion of χ up to the fourth-order, Equations (43) and (46) can be imposed to the energy minimization by defining a new functional $\mathcal{L} = \mathcal{F} + \lambda_1 f_\mathrm{c} + \lambda_2 F_\mathrm{c}$ where $\lambda_{1,2} \in \mathbb{R}$ are the Lagrange multipliers, F_c is given by Eq. (46) and the constraint

$$f_\mathrm{c} = \mathcal{D}^4 \chi + \frac{1}{R_1 R_2} = 0. \tag{47}$$

The stretching energy thus minimized by finding the set of values $\{C_k, \lambda_1, \lambda_2\}$ that solve the linear system

$$\begin{cases} \dfrac{\partial \mathcal{L}}{\partial C_k} = 0 \\ \dfrac{\partial \mathcal{L}}{\partial \lambda_{1,2}} = 0 \end{cases} \tag{48}$$

thus determining χ which further fully determines the stress and strain fields *via* Eqs. (26) and (32) needed for the X-ray diffraction calculations as detailed in Section 1.2. In the case of expansions of χ higher than fourth order, additional linear relations of C_k need to be added to the system to ensure that the constraint (47) holds for all x and y.

1.2. Calculation of the diffracted X-ray intensities

In conjunction with the pure bending strain field, the transverse stretching part has a significant contribution to the X-ray diffraction properties of the crystal due to the reactive strain perpendicular to the diffractive crystal planes mediated by the off-diagonal elements of the compliance matrix, which is also known as the Poisson effect. According to Hooke's law [Eq. (26)], these components in terms of the transverse

stretching stress are

$$u_{xz} = \frac{1}{2}\left(S_{41}\sigma_{xx} + S_{42}\sigma_{yy} + S_{46}\sigma_{xy}\right) \tag{49}$$

$$u_{yz} = \frac{1}{2}\left(S_{51}\sigma_{xx} + S_{52}\sigma_{yy} + S_{56}\sigma_{xy}\right) \tag{50}$$

$$u_{zz} = S_{31}\sigma_{xx} + S_{32}\sigma_{yy} + S_{36}\sigma_{xy}. \tag{51}$$

For the isotropic case, the components u_{xz} and u_{yz} vanish and the remaining one reduces to

$$u_{zz} = -\frac{\nu(u_{xx} + u_{yy})}{1-\nu} = -\frac{\nu}{E}(\sigma_{xx} + \sigma_{yy}) \tag{52}$$

In principle, the calculated total strain field of the pure bending and stretching components can be directly used as a deformation term in the Takagi-Taupin equations but it is computationally a daunting task for a three-dimensional macroscopic crystal. However, as shown previously in (Honkanen *et al.*, 2016), the problem can be substantially reduced by solving the diffraction curve from the Takagi-Taupin equations using the depth-dependent bending strain ϵ'_{ij} and convolving the resulting curve with the contribution due to the stretching strain u_{ij} which is assumed to be constant in the diffraction domain of any single ray. Assuming that the incidence angle of the X-rays are fixed and the wavelength is varied, the mean wavelength λ of the pure bending diffraction curve is changed due to u_{ij} by an amount $\Delta\lambda$ according to Eq. (11) of (Honkanen *et al.*, 2016):

$$\frac{\Delta\lambda}{\lambda} = \frac{\partial(\mathbf{u}\cdot\hat{\mathbf{h}})}{\partial s_{\parallel}} + \frac{\partial(\mathbf{u}\cdot\hat{\mathbf{h}})}{\partial s_{\perp}}\cot\theta_B \tag{53}$$

where $\mathbf{u}$ is the displacement vector corresponding to the stretching strain tensor u_{ij}, $s_{\parallel}$ and $s_{\perp}$ are directions parallel and perpendicular to the reciprocal lattice vector $\mathbf{h}$ ($\hat{\mathbf{h}} = \mathbf{h}/|\mathbf{h}|$) and θ_B is the Bragg angle, as presented in Figure 2 of the main text. Assuming that the diffraction takes place in the xz-plane, Eq. (53) can be written in

terms of photon energy $\mathcal{E} = hc/\lambda$ as

$$\frac{\Delta\mathcal{E}}{\mathcal{E}} = -\frac{\partial u_z}{\partial z}\cos^2\varphi - \left(\frac{\partial u_x}{\partial z} + \frac{\partial u_z}{\partial x}\right)\sin\varphi\cos\varphi - \frac{\partial u_x}{\partial x}\sin^2\varphi$$
$$- \left[\frac{\partial u_z}{\partial x}\cos^2\varphi + \left(\frac{\partial u_x}{\partial x} - \frac{\partial u_z}{\partial z}\right)\sin\varphi\cos\varphi - \frac{\partial u_x}{\partial z}\sin^2\varphi\right]\cot\theta_B, \quad (54)$$

where the asymmetry angle φ is measured between z-axis and $\mathbf{h}$, clockwise-positive. Since the strain is assumed to be constant in the volume of interest, the components of the displacement vector can be written as

$$u_x = u_x^{(0)} + u_x^{(1)}x + u_x^{(2)}z \qquad u_z = u_z^{(0)} + u_z^{(1)}x + u_z^{(2)}z \quad (55)$$

where $u_x^{(i)}$ and $u_z^{(i)}$ are constants with respect to x and z. Taking the partial derivatives of u_x and u_z and comparing to $u_{ij} = (\partial_i u_j + \partial_j u_i)/2$ (note that the term containing derivatives of ζ can be omitted as it is of the second order), we find that

$$u_x = u_x^{(0)} + u_{xx}x + u_x^{(2)}z \qquad u_z = u_z^{(0)} + (2u_{xz} - u_x^{(2)})x + u_{zz}z. \quad (56)$$

Since the bottom of the wafer is in contact with the substrate, this means that $u_z = 0$ at the wafer-substrate interface for every x. Therefore we find that $u_x^{(2)} = 2u_{xz}$ and thus the partial derivatives of u_x and u_z are

$$\frac{\partial u_x}{\partial x} = u_{xx} \qquad \frac{\partial u_x}{\partial z} = 2u_{xz} \qquad \frac{\partial u_z}{\partial x} = 0 \qquad \frac{\partial u_z}{\partial z} = u_{zz}. \quad (57)$$

Substituting these into Eq. (54) thus allows us to write the energy shift in terms of the strain tensor:

$$\frac{\Delta\mathcal{E}}{\mathcal{E}} = -\, u_{zz}\cos^2\varphi - 2u_{xz}\sin\phi\cos\varphi - u_{xx}\sin^2\varphi$$
$$+ \left[(u_{zz} - u_{xx})\sin\varphi\cos\varphi + 2u_{xz}\sin^2\varphi\right]\cot\theta_B \quad (58)$$

which in the symmetric Bragg case simplifies to

$$\frac{\Delta\mathcal{E}}{\mathcal{E}} = -u_{zz}. \quad (59)$$

The diffraction (or resolution) curve of the whole crystal wafer is then obtained by calculating the distribution $\rho_{\Delta\mathcal{E}}$ of energy shifts $\Delta\mathcal{E}$ over the surface and convolving the resulting distribution with the depth-dependent Takagi-Taupin curve solved for the pure bending solution Eq. (23). How to use the Takagi-Taupin theory for diffraction calculations is explained elsewhere *e.g.* in (Gronkowski, 1991) and will not be pursued further in this work.

Formally $\rho_{\Delta\mathcal{E}}(\varepsilon)$ for a particular energy shift ϵ is obtained by summing all the surface elements $d\Omega$ whose energy shift $\Delta\mathcal{E} = \varepsilon$ *i.e.*

$$\rho_{\Delta\mathcal{E}}(\varepsilon) \propto \int_\Omega d\Omega \; \delta(\Delta\mathcal{E} - \varepsilon) \tag{60}$$

where δ is the Dirac delta function and $\Delta\mathcal{E} = \Delta\mathcal{E}(x, y)$ is understood to be a function of position. Similarly, for rocking curve measurements with a monochromatic beam, the shifts in the diffraction angle are

$$\begin{aligned}\Delta\theta = &- \left(u_{zz}\cos^2\varphi + 2u_{xz}\sin\varphi\cos\varphi + u_{xx}\sin^2\varphi\right)\tan\theta_B \\ &+ (u_{zz} - u_{xx})\sin\varphi\cos\phi + 2u_{xz}\sin^2\varphi\end{aligned} \tag{61}$$

which in the symmetric Bragg case simplifies to

$$\Delta\theta = -u_{zz}\tan\theta_B. \tag{62}$$

Note that Eq. (62) ceases to be valid near $\theta_B = \pi/2$ since it is based on the first order Taylor expansion. The corresponding distribution as a function of shift angle α is

$$\rho_{\Delta\theta}(\alpha) \propto \int_\Omega d\Omega \; \delta(\Delta\theta - \alpha). \tag{63}$$

The contribution of energy or angular shifts to the resolution in the respective scan domains can be estimated by calculating the standard deviation of the appropriate distribution.

Usually changes in both $\mathcal{E}$ and $\tan\theta_B$ are minute during scans which means that they can be considered constants. Thus the distributions of ΔE and $\Delta\theta$ differ only by

a multiplicative factor. Therefore, for the sake of brevity, only the derivation of the ΔE distributions is presented in the following section.

2. Important special cases

In this section we apply the general framework to derive a few important results that are especially relevant considering current trends in the contemporary instrument design. Transverse stretching strain and stress fields due to toroidal bending are derived for circular and rectangular wafers of elastically anisotropic materials, due to their prevalent use in the crystal analyser. In addition, their isotropic counterparts are derived and analysed separately to obtain simplified models for better understanding of anisotropic models and quick analytical estimation of various diffraction properties.

In derivations special attention is put on the spherical bending for three reasons: 1) most of the current state-of-the-art TBCA:s belong to this subclass, 2) availability of the experimental diffraction curves, and 3) it is less complicated to derive the more general toroidal models through examining the spherical bending. The last point becomes evident when we examine the energy minimization constraints. By denoting $R \equiv R_1 = R_2$, the first constraint [Eq. (47)] becomes

$$f_\mathrm{c} = \mathcal{D}^4 \chi + \frac{1}{R^2} = 0, \tag{64}$$

from which the toroidal case can be fully recovered if we replace the spherical bending radius with the geometrical mean of the toroidal bending radii *i.e.* $R \to \sqrt{R_1 R_2}$. Therefore the only real difference between the toroidal and spherical bending may arise from the second, contact force constraint of Eq. (46). However, it turns out that in the cases examined in the following, a solution obtained from minimizing the energy using only the first constraint fulfils automatically also the second one. Therefore, it is sufficient to find a solution using the spherical case and to show that it leads to a vanishing contact force in the toroidal case.

2.1. Isotropic circular wafer

Consider a spherically bent, isotropic circular crystal wafer with the diameter L and bending radius R. As per to the general approach, we could use a truncated series in terms of x/R and y/R as an ansatz for the sought-after χ. However, since the physical system possesses the perfect radial planar symmetry, we can also find the exact solution to the problem with relative ease.

The formal solution to spherical constraint Eq. (44) is the sum of the general solution to the homogeneous biharmonic equation $\nabla^4 \chi_0 = 0$ and any special solution to nonhomogeneous equation. In polar coordinates (r, ϕ) the general solution to the homogeneous biharmonic equation is known as the Michell solution (Michell, 1899). For a radially symmetric problem, the solution is required to be independent of ϕ so the Michell solution simplifies to $\chi_0 = A_0 r^2 + B_0 r^2 \ln r + C_0 \ln r$, where A_0, B_0 and C_0 are coefficients to be determined. A special solution to Eq. (44) is $\chi_1 = -Er^4/64R^2$, which is easy to see by substitution. Thus the complete radially symmetric solution to Eq. (44) is

$$\chi = \chi_0 + \chi_1 = -\frac{E}{64R^2} r^4 + A_0 r^2 + B_0 r^2 \ln r + C_0 \ln r. \tag{65}$$

The coefficients are can now be found by minimizing the stretching energy. However, the task can be further simplified by examining the components of stress. Since σ_{ij} are given by the second derivatives of χ, we can set $B_0 = C_0 = 0$; otherwise we would obtain diverging components of the stress tensor at $r = 0$ owing to the logarithmic terms in χ. Thus from Eq. (32), we obtain

$$\sigma_{xx} = -\frac{E}{16R^2}(x^2+3y^2)+2A_0, \quad \sigma_{xy} = \frac{E}{8R^2}xy, \quad \sigma_{yy} = -\frac{E}{16R^2}(3x^2+y^2)+2A_0. \tag{66}$$

Considering the constraints of minimization, we note that the spherical bending is already enforced by the chosen form of χ, so we do not have to include the constraint (47) into the linear system (48) explicitely. Furthermore, we choose to neglect the

contact force constraint (46) for now, thus reducing the linear system to a single equation:

$$\frac{\partial \mathcal{F}}{\partial A_0} = 0. \tag{67}$$

Substituting $\partial\sigma_{xx}/\partial A_0 = \partial\sigma_{yy}/\partial A_0 = 2$ and $\partial\sigma_{xy}/\partial A_0 = 0$ to Equation (40), the condition becomes

$$\int_\Omega d\Omega\ \sigma_{xx} + \sigma_{yy} = \int_0^{2\pi} d\phi \int_0^{L/2} dr\ r \left(4A_0 - \frac{E}{4R^2} r^2\right) = 0, \tag{68}$$

where the prefactor $2d(1-\nu)$ has been dropped out. Carrying out the integration, the stretching energy is found to be minimized when

$$A_0 = \frac{EL^2}{128R^2}. \tag{69}$$

Substituting (69) back to (66), we thus obtain

$$\sigma_{xx} = \frac{E}{16R^2}\left(\frac{L^2}{4} - x^2 - 3y^2\right), \quad \sigma_{xy} = \frac{E}{8R^2}xy, \quad \sigma_{yy} = \frac{E}{16R^2}\left(\frac{L^2}{4} - 3x^2 - y^2\right). \tag{70}$$

Substituting these into the Equations (30) and (52), we obtain the following non-zero components of the strain tensor:

$$u_{xx} = \frac{1}{16R^2}\left[(1-\nu)\frac{L^2}{4} - (1-3\nu)x^2 - (3-\nu)y^2\right] \tag{71}$$

$$u_{yy} = \frac{1}{16R^2}\left[(1-\nu)\frac{L^2}{4} - (1-3\nu)y^2 - (3-\nu)x^2\right] \tag{72}$$

$$u_{xy} = \frac{1+\nu}{8R^2}xy \tag{73}$$

$$u_{zz} = \frac{\nu}{4R^2}\left(x^2 + y^2 - \frac{L^2}{8}\right) \tag{74}$$

Now, as per the discussion in the beginning of the current section, we now attempt to generalise the solution to the toroidal bending by a trivial substitution $R \to \sqrt{R_1 R_2}$. According to Eq. (45), the contact force between the wafer and the substrate per unit area is thus

$$P = \frac{Ed}{16R_1^2R_2^2}\left[(3R_1 + R_2)\,x^2 + (R_1 + 3R_2)\,y^2 - (R_1 + R_2)\,\frac{L^2}{4}\right]. \tag{75}$$

Integrating P over the surface of the wafer results in zero net force which means that the previously omitted constraint (46) is in fact fulfilled by the solution obtained without its explicit inclusion. We therefore conclude that the solution, even though derived for a spherical bending, is valid also for the toroidal case.[3]

The symmetry considered, it is convenient to give the components of the stress tensor in the cylindrical coordinates as well. Since the stress and strain tensors are second-rank contravariant tensors, they transform as

$$T'_{ij} = \sum_{k,l} \frac{\partial x'_i}{\partial x_k} \frac{\partial x'_j}{\partial x_l} T_{kl} \tag{76}$$

where T'_{ij} are the components in the new coordinate system $\{x'_i\}$ and T_{kl} are the components in the old system $\{x_k\}$. Therefore in cylindrical coordinates[4]

$$T'_{rr} = \cos^2\phi T_{xx} + 2\sin\phi\cos\phi T_{xy} + \sin^2\phi T_{yy} \tag{77}$$

$$T'_{r\phi} = -\sin\phi\cos\phi T_{xx} + (\cos^2\phi - \sin^2\phi) T_{xy} + \sin\phi\cos\phi T_{yy} \tag{78}$$

$$T'_{\phi\phi} = \sin^2\phi T_{xx} - 2\sin\phi\cos\phi T_{xy} + \cos^2\phi T_{yy} \tag{79}$$

$$T'_{rz} = \cos\phi T_{xz} + \sin\phi T_{yz} \tag{80}$$

$$T'_{\phi z} = -\sin\phi T_{xz} + \cos\phi T_{yz} \tag{81}$$

$$T'_{zz} = T_{zz}. \tag{82}$$

Thus we obtain

$$\sigma_{rr} = \frac{E}{16R^2}\left(\frac{L^2}{4} - r^2\right) \qquad \sigma_{r\phi} = 0 \qquad \sigma_{\phi\phi} = \frac{E}{16R^2}\left(\frac{L^2}{4} - 3r^2\right). \tag{83}$$

[3] This is despite the fact that we assumed the ansatz of χ to be circularly symmetric, as the bending radii enter the free energy minimization only through their product.

[4] The angular coordinate ϕ is actually handled here as $r\phi$ in order to keep the physical unit of the coordinates and thus the dimensions of the transformed tensor components consistent with the Cartesian representation.

Similarly for the strain tensor we have

$$u_{rr} = \frac{1}{16R^2}\left[(1-\nu)\frac{L^2}{4} - (1-3\nu)r^2\right] \tag{84}$$

$$u_{\phi\phi} = \frac{1}{16R^2}\left[(1-\nu)\frac{L^2}{4} - (3-\nu)r^2\right] \tag{85}$$

$$u_{r\phi} = 0 \tag{86}$$

$$u_{zz} = \frac{\nu}{4R^2}\left(r^2 - \frac{L^2}{8}\right) \tag{87}$$

We find that the radial normal stress σ_{rr} vanishes at the edge of the wafer which implies that no external forces are acting normal to the edge of the wafer in the minimum energy solution. This is in correspondence with physical crystal wafers whose edges are laterally freestanding *i.e.* not in contact with anything that would apply in-plane compression or extension to them. Also the shear components $\sigma_{r\phi}$ and $u_{r\phi}$ are zero everywhere which can be interpreted that the crystal is not twisted about the z-axis. However, the most interesting behaviour is expressed by the angular normal stress $\sigma_{\phi\phi}$ which is negative near the edge and changes sign at $r = L/\sqrt{12}$. This is a natural consequence from the geometrical fact that the flat wafer cannot fit on a toroidal surface without transverse (in-plane) deformation. The minimization of the deformation energy indicates that the edges of the wafer are compressed angularly but extended in-plane near the center to adopt the toroidal shape. This is in contrast to the previous work where the wafer was assumed to be unstretched at the center and compressed at the edge (Honkanen *et al.*, 2014). The discrepancy arises from the fact that the previous approach was based solely on the geometrical considerations of the spherical bending which does not fix the value of the elastic energy of the wafer. The requirement of the energy minimization does not alter the resolution curve drastically but does lead to *e.g.* non-vanishing integrated contact force on the wafer–substrate interface. The derivation presented in this work is theoretically more sound and thus expected to be physically more accurate.

As a curiosity it is interesting to note that the qualitative behaviour of u_{rr} is different for $\nu < 1/3$ and $\nu > 1/3$. Whereas in the former case the radial strain is largest at the centre of the wafer, in the latter it is largest at the edge. This is because the radial and angular normal stresses σ_{rr} and $\sigma_{\phi\phi}$, respectively, influence u_{rr} in the opposite manner and whichever is the dominant factor is determined by the value of Poisson's ratio.

Using Equation (59), we find that the energy shift $\Delta\mathcal{E}$ as a function of surface position is

$$\frac{\Delta\mathcal{E}}{\mathcal{E}} = -\frac{\nu}{4R^2}\left(r^2 - \frac{L^2}{8}\right). \tag{88}$$

The isocurves of the energy shift are circular as one would expect on the basis of the radial symmetry. Substituting the obtained $\Delta\mathcal{E}$ to Eq. (60) and carrying out the integration, we find the energy shift distribution

$$\rho_{\Delta\mathcal{E}}(\varepsilon) = \begin{cases} \text{constant}, & -\frac{\nu L^2 \mathcal{E}}{32R^2} \leq \varepsilon \leq \frac{\nu L^2 \mathcal{E}}{32R^2} \\ 0 & \text{otherwise} \end{cases} \tag{89}$$

The found uniform distribution can be used to convolve the depth-dependent Takagi-Taupin solution to predict the diffraction curve of an TBCA.

To quickly estimate the effect of transverse strain to the energy resolution, we note that the variance of a uniform distribution with a width of w is $w^2/12$ and thus the standard deviation of the energy shift distribution (89) is

$$\sigma = \frac{\nu L^2 \mathcal{E}}{32\sqrt{3}R^2}. \tag{90}$$

The standard deviation due to transverse strain can be then combined with the standard deviations of other contributions (depth-dependent Takagi-Taupin solution, incident bandwidth, etc.) by quadratic summing in accordance with the central limit theorem. Usually the full-width-at-half-maximum (FWHM) is used instead of the standard deviation, in the case of which σ is to be multiplied by $2\sqrt{2\ln 2}$. This underestimates

the true FWHM of Eq. (89) approximately by a factor of 0.68 but, regarding the central limit theorem, gives more accurate contribution to the total FWHM.

2.2. Anisotropic circular wafer

The solving procedure follows the same steps for elastically anisotropic crystals as for the isotropic case. However, since the anisotropy of the crystal does not generally follow the symmetry of the wafer, we should relax the circular symmetry requirement for the ansatz of χ as well. In general, the candidate solution can be written as a polynomial series of x/R and y/R:

$$\chi(x,y) = \sum_{m,n=0}^{\infty} C_{m,n} \left(\frac{x}{R}\right)^m \left(\frac{y}{R}\right)^n \tag{91}$$

For a typical crystal analyser x/R and y/R are order of 0.1 or less. Thus we may opt to truncate the series representation of χ up to the few lowest orders. Substituting Eq. (91) into the nonhomogeneous biharmonic equation (44), we find that the simplest solution is of the fourth order. Expanded, the ansatz is then

$$\begin{aligned}\chi = C_{11}xy + \frac{1}{2}\left(C_{20}x^2 + C_{02}y^2 + C_{21}x^2y + C_{12}xy^2 + C_{22}x^2y^2\right) \\ + \frac{1}{3}\left(C_{31}x^3y + C_{13}xy^3\right) + \frac{1}{6}\left(C_{30}x^3 + C_{03}y^3\right) + \frac{1}{12}\left(C_{40}x^4 + C_{04}y^4\right)\end{aligned} \tag{92}$$

where the numerical prefactors are added for the subsequent convenience. Coefficients C_{00}, C_{10}, and C_{01} are set to zero since they do not affect the stress tensor components. Using Eq. (32), the transverse stress tensor components are

$$\sigma_{xx} = C_{02} + C_{12}x + C_{22}x^2 + C_{03}y + 2C_{13}xy + C_{04}y^2 \tag{93}$$

$$\sigma_{yy} = C_{20} + C_{21}y + C_{22}y^2 + C_{30}x + 2C_{31}xy + C_{40}x^2 \tag{94}$$

$$\sigma_{xy} = -C_{11} - C_{21}x - C_{12}y - C_{31}x^2 - C_{13}y^2 - 2C_{22}xy \tag{95}$$

The bending constraint (47) now becomes

$$f_c = S_{11}C_{04} + S_{22}C_{40} + (2S_{12} + S_{66})C_{22} - 2(S_{16}C_{13} + S_{26}C_{31}) + \frac{1}{2R^2} = 0. \tag{96}$$

Omitting the contact force constraint (46) at this stage, the coefficients C_{ij} are solved by minimizing the constrained stretching energy which is presented in Appendix D. The resulting stretching stress tensor components are

$$\sigma_{xx} = \frac{E'}{16R^2}\left(\frac{L^2}{4} - x^2 - 3y^2\right) \quad \sigma_{yy} = \frac{E'}{16R^2}\left(\frac{L^2}{4} - 3x^2 - y^2\right) \quad \sigma_{xy} = \frac{E'}{8R^2}xy \quad (97)$$

where

$$E' = \frac{8}{3(S_{11} + S_{22}) + 2S_{12} + S_{66}} \quad (98)$$

which, in comparison to stresses obtained in the isotropic case [Eq. (70)], can be interpreted as effective Young's modulus. For isotropic crystal $E' = E$ but in general $E' \neq 1/S_{11}$.

Since the form of the obtained stresses is identical to that of the isotropic case, the immediate implication is that the contact force is equivalent to Eq. (75) when effective Young's modulus is used. Therefore the obtained anisotropic solution also is generalisable to the toroidal bending by the trivial substitution $R \to \sqrt{R_1 R_2}$.

Substituting the obtained stresses to Eqs. (27)–(29) and (49)–(51) gives the following strain tensor components:

$$u_{xx} = \frac{E'}{16R^2}\left[(S_{11} + S_{12})\frac{L^2}{4} - (S_{11} + 3S_{12})x^2 - (3S_{11} + S_{12})y^2 + 2S_{16}xy\right] \quad (99)$$

$$u_{yy} = \frac{E'}{16R^2}\left[(S_{21} + S_{22})\frac{L^2}{4} - (S_{21} + 3S_{22})x^2 - (3S_{21} + S_{22})y^2 + 2S_{26}xy\right] \quad (100)$$

$$u_{zz} = \frac{E'}{16R^2}\left[(S_{31} + S_{32})\frac{L^2}{4} - (S_{31} + 3S_{32})x^2 - (3S_{31} + S_{32})y^2 + 2S_{36}xy\right] \quad (101)$$

$$u_{xz} = \frac{E'}{32R^2}\left[(S_{41} + S_{42})\frac{L^2}{4} - (S_{41} + 3S_{42})x^2 - (3S_{41} + S_{42})y^2 + 2S_{46}xy\right] \quad (102)$$

$$u_{yz} = \frac{E'}{32R^2}\left[(S_{51} + S_{52})\frac{L^2}{4} - (S_{51} + 3S_{52})x^2 - (3S_{51} + S_{52})y^2 + 2S_{56}xy\right] \quad (103)$$

$$u_{xy} = \frac{E'}{32R^2}\left[(S_{61} + S_{62})\frac{L^2}{4} - (S_{61} + 3S_{62})x^2 - (3S_{61} + S_{62})y^2 + 2S_{66}xy\right]. \quad (104)$$

Expressed in polar coordinates, the components of the stress tensor are

$$\sigma_{rr} = \frac{E'}{16R^2}\left(\frac{L^2}{4} - r^2\right) \qquad \sigma_{r\phi} = 0 \qquad \sigma_{\phi\phi} = \frac{E'}{16R^2}\left(\frac{L^2}{4} - 3r^2\right) \quad (105)$$

and the most important strain tensor component[5] from the viewpoint of diffraction calculations is given by

$$u_{zz} = \frac{E'}{16R^2}\left[(S_{31}+S_{32})\frac{L^2}{4} - \left[2(S_{31}+S_{32}) + \sqrt{(S_{32}-S_{31})^2 + S_{36}^2}\cos(2\phi+\beta)\right] r^2\right] \tag{106}$$

where $\beta = \mathrm{atan}[S_{36}/(S_{32}-S_{31})]$.

The symmetric stress tensor is expected to be radially symmetric since transversally anisotropic stress would even itself out, as argued previously in (Honkanen *et al.*, 2014). However, the symmetry is broken in the strain tensor due to the anisotropic elastic properties of the crystal. Generally the isocurves of u_{zz} are elliptical whereas for the isotropic case they are circular. The derived expression for u_{zz} is otherwise identical to the previously found result in (Honkanen *et al.*, 2014) except for the constant term proportional to L^2. As discussed in the previous subsection, this is due to the fact that in the previous geometrically based method the total elastic energy was not considered. However, it should be noted that the original approach leads to the same solution if the integrated contact force is required to vanish.

As for the isotropic case, the shifts $\Delta\mathcal{E}$ in the diffraction energy are obtained from Eq. (59). By substituting to Eq. (60) and carrying out the radial integration we find that

$$\rho_{\Delta\mathcal{E}}(\varepsilon) \propto \int_0^{2\pi} d\phi\; \Gamma(\phi,\varepsilon) \tag{107}$$

where

$$\Gamma(\phi,\varepsilon) = \begin{cases} \dfrac{1}{2A + B\cos 2\phi} & \text{when} \quad -A - B\cos 2\phi < \varepsilon < A \\ 0 & \text{otherwise} \end{cases} \tag{108}$$

where the constants are

$$A = -\frac{(S_{31}+S_{32})E'L^2\mathcal{E}}{64R^2} \qquad B = \frac{E'L^2\mathcal{E}}{64R^2}\sqrt{(S_{32}-S_{31})^2 + S_{36}^2}. \tag{109}$$

[5] For the sake of brevity, the other components are not presented here as transforming them using Eqs. (77)–(82) is straightforward but the results are lengthy and give little extra value to the discussion of the topic at hand.

Note that β has been dropped from the argument of the cosine for simplicity since the integration goes over 2π. Furthermore from the symmetry of $\cos 2\phi$ it follows that the integrating Eq. (107) over 2π is equal to integration over $[0, \pi/2]$ and multiplying the result by 4. Thus

$$\rho_{\Delta\mathcal{E}}(\varepsilon) \propto \int_0^{\pi/2} d\phi\ \Gamma(\phi, \varepsilon). \tag{110}$$

Now since $\mathrm{acos}(x)/2$ can be uniquely mapped over the shortened integration range, we can find an angle $0 < \phi_0 < \pi/2$ above which the inequality $\varepsilon > -A - B\cos 2\phi$ ceases to be valid. Therefore we may get rid of the piecewise definition of $\Gamma(\phi, \varepsilon)$ by replacing the upper limit in the integral Equation (110) with

$$\phi_0(\varepsilon) = \frac{1}{2}\,\mathrm{acos}\,\frac{-A-\varepsilon}{B} \tag{111}$$

and thus obtain

$$\begin{aligned}\rho_{\Delta\mathcal{E}}(\varepsilon) \propto \int_0^{\phi_0(\varepsilon)} d\phi\ \frac{1}{2A + B\cos 2\phi} &= \frac{1}{\sqrt{4A^2 - B^2}}\,\mathrm{atan}\left[\frac{(2A - B)\tan\phi_0(\varepsilon)}{\sqrt{4A^2 - B^2}}\right] \\ &= \frac{1}{\sqrt{4A^2 - B^2}}\,\mathrm{atan}\sqrt{\frac{(B-2A)(\varepsilon + A + B)}{(B+2A)(\varepsilon + A - B)}}\end{aligned} \tag{112}$$

when $-A - B < \varepsilon < -A + B$. In the interval $-A + B \leq \varepsilon < A$ the integral (110) evaluates to a constant which is found by taking the limit $\phi_0(\varepsilon) \to \pi/2$ of Eq. (112). Thus we find the energy shift distribution

$$\rho_{\Delta\mathcal{E}}(\varepsilon) = k \times \begin{cases} \mathrm{atan}\sqrt{\dfrac{(B-2A)(\varepsilon + A + B)}{(B+2A)(\varepsilon + A - B)}} & -A - B < \varepsilon < -A + B \\ \dfrac{\pi}{2} & -A + B \leq \varepsilon < A \\ 0 & \text{otherwise} \end{cases} \tag{113}$$

where $k > 0$ is a proportionality constant. Plots of Equation (113) with a selected values of B/A are presented in Figure 3 of the main text. When $B = 0$, the situation is equivalent to that of the isotropic circular case as the distribution of energy shifts is found to be constant and the energy shift isocurves traced over the crystal surface are perfect circles. For non-zero B, the isocurves become elliptical which means that they

are intercepted by the circular edge away from the wafer centre, as illustrated in Fig. 4 of the main text. The discontinuous isocurves influence the energy shift distribution by introducing a tail on the low energy side of the curve whose prominence is proportional to B/A ratio.

An important practical implication of elliptical isocurves is that there is a specific direction along the surface in which the energy shift varies fastest. Since S_{31} and S_{32} are negative, the gradient of u_{zz} as per to Eq. (106) is steepest in the radial direction when $\cos(2\phi + \beta) = -1$ *i.e.* $\phi = (-\beta \pm \pi)/2$. This has relevance in regards to the resolution function in cases where the surface area of a TBCA needs to be limited transversally in one direction *e.g.* to minimize the Johann error by masking the surface, or to reduce the space occupied by the analyser by cutting its edges off. To optimize the intrinsic resolution of the analyser, the surface area should be reduced where the gradient is steepest.[6] For example, masking the edges of a spherical Si(660) analyser with 100 mm diameter and 1 m bending radius using a 80 mm wide slit can improve the energy resolution (measured from the standard deviation) by 13% in near-backscattering conditions if the mask is aligned over the direction of the steepest gradient, which is $[1\bar{1}0]$. However, in the worst-case scenario when the mask is oriented perpendicular to the optimal case, the resolution *degrades* by 3% in comparison to the unmasked crystal. In the worst case, the resolution of the SBCA in question can thus be 18% worse than with optimal masking/cutting which is not a negligible detriment. The directions of steepest gradient for selected crystal planes in cubic systems are listed in Table 1.

To estimate the contribution of transverse strain to the energy resolution, the standard deviation of Eq. (113) can be calculated from the first and second moments of

[6] The cut SBCAs in the X-ray Raman scattering spectrometer at the beamline ID20 at ESRF are optimized in this manner (Huotari *et al.*, 2017).

the normalized distribution, and is found to be

$$\sigma = \frac{\nu' L^2 \mathcal{E}}{32\sqrt{3}R^2}\sqrt{1+\frac{K^2}{2}} \tag{114}$$

where we have introduced the effective Poisson's ratio

$$\nu' \equiv -\frac{4(S_{31}+S_{32})}{3(S_{11}+S_{22})+2S_{12}+S_{66}} \tag{115}$$

and the eccentricity factor

$$K \equiv \frac{B}{A} = -\frac{\sqrt{(S_{32}-S_{31})^2+S_{36}^2}}{S_{31}+S_{32}}. \tag{116}$$

The FWHM compliant with the central limit theorem is obtained by multiplying σ by $2\sqrt{2\ln 2}$. In the isotropic case $\nu' = \nu$ and $K = 0$, thus reducing Eq. (114) expectedly to Eq. (90). For convenience, Table 1 tabulates the effective Young's moduli, Poisson ratios, and eccentricity factors for selected crystal plane directions of Si and Ge.

The predictions of the anisotropic circular model were calculated for four different types of SBCA and compared to two separate experimental data sets acquired at ESRF and first published in (Honkanen *et al.*, 2014) and (Rovezzi *et al.*, 2017). In Figure 5 of the main text are presented the reflectivity curves measured in near-backscattering conditions from three Si(660) and two Si(553) analysers all with the bending radius of 1 m, 100 mm diameter and 300 μm wafer thickness. The curves were acquired using two circular masks with aperture diameters of 30 mm and 60 mm, and without mask (aperture 100 mm). Figure 6 of the main text presents the comparison of the current model with and without the contribution of Johann error (Johann, 1931) to the reflectivity curves measured at two different Bragg angles of two Si(555) circular analysers with the bending radii of 1 m and 0.5 m. The diameter and thickness of the wafers were 100 mm and 150 μm, respectively. Further experimental details are presented in the original sources.

Compared with the previous work which was based on the geometrical considerations and did not account for the minimization of the elastic energy, slight differences

between two models are observed but they are found to be less than the variation between different SBCA units, as seen in Fig. 5 of the main text. This excludes one explanation put forth in the previous work for the discrepancy between the data and the model at the low-energy tail of the diffraction curve for the full analyser, according to which the observed difference could be due to non-vanishing σ_{rr} at the wafer edge in the previous model. One possible explanation to the discrepancy is the imperfections in manufacturing process, as it is found that the figure error in anodically bonded analysers is largest at the edge (Verbeni *et al.*, 2005). Another explanation could be a slight deviation from the Rowland circle geometry that is not included in the calculations. The latter hypothesis is supported by the data in Fig. 6 of the main text where the deviations are more prominent. According to the theory, the stresses and strains due to stretching are a factor of 4 larger in a wafer that has half the bending radius than in a wafer otherwise identical. Even for considerably higher transverse stress, the theory predicts correctly the observed boxcar shape and its width for the measured 0.5 m Si(555) analyser. The general shape and the width of the predicted 1 m Si(555) curve are in line with the measurements but is not as precise as for the set of Si(660) and Si(553) analysers in Fig. 5 of the main text. The most probable reason for this is the contribution of the aforementioned deviation from the Rowland circle geometry, the effect of which is amplified at lower Bragg angles. In the experimental description, it is mentioned that the radius of the Rowland circle was adjusted by optimizing the product of total counts and peak intensity divided by the FWHM for each analyser (Rovezzi *et al.*, 2017). Since the different contributions to the energy resolution of an SBCA are not truly independent of each other, such an optimization can lead to partial cancellation of some contribution by another and thus lead to a better resolution than expected in the exact Rowland circle configuration. Therefore to accurately characterise the elastic contribution to resolution functions of SBCAs,

the near-backscattering condition is recommended to minimise the geometrical effects.

2.3. Isotropic rectangular wafer

We assume that a spherically bent, rectangular crystal wafer is centred at $x = y = 0$ with sides of length a and b aligned parallel with x- and y-axes, respectively. Since the wafer is symmetric under transformations $x \to -x$ and $y \to -y$, we immediately conclude that the series Eq. (91) can contain only even terms *i.e.* $C_{m,n} = 0$ if either m or n is odd. Thus we arrive at the fourth-order ansatz

$$\chi(x,y) = \frac{1}{2}\left(C_{20}x^2 + C_{02}y^2 + C_{22}x^2y^2\right) + \frac{1}{12}\left(C_{40}x^4 + C_{04}y^4\right), \tag{117}$$

with the added numerical prefactors. In addition, we set $C_{00} = 0$ since it has no contribution to the sought-after stress tensor. Thus using Equations (32) we obtain from (117)

$$\sigma_{xx} = C_{22}x^2 + C_{04}y^2 + C_{02}, \quad \sigma_{xy} = -2C_{22}xy, \quad \sigma_{yy} = C_{22}y^2 + C_{40}x^2 + C_{20}. \tag{118}$$

The coefficients C_{ij} are found by minimizing the stretching energy $\mathcal{F}$ under the requirement that χ fulfils the non-homogeneous biharmonic equation (44). The details of the minimization are presented in Appendix E. As a result, the following stretching strain tensor components are found:

$$\sigma_{xx} = \frac{E}{gR^2}\left[\frac{a^2}{12} - x^2 + \left(\frac{1+\nu}{2} + 5\frac{a^2}{b^2} + \frac{1-\nu}{2}\frac{a^4}{b^4}\right)\left(\frac{b^2}{12} - y^2\right)\right] \tag{119}$$

$$\sigma_{yy} = \frac{E}{gR^2}\left[\frac{b^2}{12} - y^2 + \left(\frac{1+\nu}{2} + 5\frac{b^2}{a^2} + \frac{1-\nu}{2}\frac{b^4}{a^4}\right)\left(\frac{a^2}{12} - x^2\right)\right] \tag{120}$$

$$\sigma_{xy} = \frac{2E}{gR^2}xy, \tag{121}$$

where

$$g = 8 + 10\left(\frac{a^2}{b^2} + \frac{b^2}{a^2}\right) + (1-\nu)\left(\frac{a^2}{b^2} - \frac{b^2}{a^2}\right)^2. \tag{122}$$

We now assume that the obtained solution for the stresses is valid also for the general toroidal bending. From Eq. (45) we find the contact force per unit area to be

$$P = -\frac{Ed}{gR_1^2R_2^2}\Bigg[\left(R_1\left(\frac{1+\nu}{2}+5\frac{b^2}{a^2}+\frac{1-\nu}{2}\frac{b^4}{a^4}\right)+R_2\right)\left(\frac{a^2}{12}-x^2\right) + \left(R_2\left(\frac{1+\nu}{2}+5\frac{a^2}{b^2}+\frac{1-\nu}{2}\frac{a^4}{b^4}\right)+R_1\right)\left(\frac{b^2}{12}-y^2\right)\Bigg] \quad (123)$$

Integrating P over the analyser surface results in zero net contact force, which indicates that the constrained omitted in the minimization is automatically fulfilled and the obtained solution is indeed generalisable to the toroidal by a trivial substitution $R \to \sqrt{R_1R_2}$.

An interesting observation is that, contrary to the case of circular wafers, at the edges of the wafer the stress tensor elements describing the normal stress perpendicular to the edge do not vanish. One could argue that the order of the ansatz used is not high enough. However, at least up to the eighth-order, it turns out that requiring the solution to simultaneously to fulfil Eq. (44) and lead to vanishing normal stress at the edges is not possible unless the expansion coefficients of χ higher that the fourth-order are zero. Fixing the normal component of the stress at the edges completely determines the solution in the fourth order that is necessarily less relaxed than the one obtained through the minimization of energy in Appendix E. Further, it turns out that the integrated contact force [Eq. (46)] of such a solution is non-zero, which is incompatible with the assumption that the wafer is bent and held onto the spherical substrate by the adhesive force between the wafer and substrate alone. Thus it seems that non-zero normal stress at the edges of the wafer is a real part of the rectangular model arising from the mechanical contact between a rectangular wafer and the spherical surface and not an artefact due to the low-order polynomial ansatz. However, since the physical wafer is of finite thickness, the toroidal surface can not possibly support the lateral edges above the immediate contact between the wafer and the surface.

This implies that the true physical stretching strain field can have a more complicated form than assumed in the method and thus cause deviations between the predicted and measured diffraction curves at the wafer edges. More in-depth analysis of the edge region is, however, beyond the scope of this work.

Substituting Eqs. (119) and (120) to Eq. (52), the most relevant strain tensor component for the diffraction calculations is thus found to be

$$u_{zz} = \frac{\nu}{gR^2}\Bigg[\left(\frac{3+\nu}{2} + 5\frac{b^2}{a^2} + \frac{1-\nu}{2}\frac{b^4}{a^4}\right)\left(x^2 - \frac{a^2}{12}\right) + \left(\frac{3+\nu}{2} + 5\frac{a^2}{b^2} + \frac{1-\nu}{2}\frac{a^4}{b^4}\right)\left(y^2 - \frac{b^2}{12}\right)\Bigg] \tag{124}$$

Equation (124) for three different a/b ratios is visualised in Figure 7 of the main text. In general, the crystal planes normal to the surface are compressed in the center of the wafer and expanded at the edges, which is reactionary to transverse extension at the center and contraction at the edges of the wafer via non-zero Poisson's ratio. The isocurves of u_{zz} are found to be elliptical in shape, albeit being cut near the edges of the wafer. The major axis of the isocurves are along the longer dimension of the wafer and the strain grows fastest along the minor axes. For the special case of $a = b$, the isocurves become circles following the symmetry of the crystal similar to the isotropic circular wafer. It is interesting to note that whereas in the case of circular wafer non-circular isocurves result from the breaking of radial symmetry by the anisotropy of elastic properties of the crystal, for the rectangular wafer it is broken by lifting the 90° rotation symmetry.

As before, the energy shifts according to Eq. (59) are $\Delta\mathcal{E} = -u_{zz}\mathcal{E}$. Substituting this to Eq. (60), utilizing the symmetries and carrying out the integration along x results to

$$\rho_{\Delta\mathcal{E}}(\varepsilon) \propto \int_0^{b/2} dy \begin{cases} \dfrac{1}{\sqrt{C - \varepsilon - By^2}} & \text{when} \quad 0 < C - \varepsilon - By^2 < \frac{Aa^2}{4} \\ 0 & \text{otherwise} \end{cases} \tag{125}$$

where

$$A = \frac{\nu\mathcal{E}}{gR^2}\left(\frac{3+\nu}{2} + 5\frac{b^2}{a^2} + \frac{1-\nu}{2}\frac{b^4}{a^4}\right) \quad B = \frac{\nu\mathcal{E}}{gR^2}\left(\frac{3+\nu}{2} + 5\frac{a^2}{b^2} + \frac{1-\nu}{2}\frac{a^4}{b^4}\right)$$
$$C = \frac{Aa^2 + Bb^2}{12}. \tag{126}$$

By performing a change of the integration variable, Eq. (125) becomes

$$\rho_{\Delta\mathcal{E}}(\varepsilon) \propto \int_0^{Bb^2/4} du \begin{cases} \frac{1}{\sqrt{(C-\varepsilon)u - u^2}} & \text{when} \quad C - \varepsilon - \frac{Aa^2}{4} < u < C - \varepsilon \\ 0 & \text{otherwise} \end{cases} \tag{127}$$

The indefinite solution to the integral is $2\,\mathrm{atan}(\sqrt{u/(C-\varepsilon-u)})$ but the integration range is altered by the limits imposed on u. Depending whether $Aa^2 > Bb^2$ or $Aa^2 < Bb^2$, the integration ranges as a piecewise function of ε can be classified respectively to the Case `I` or `II` as indicated by Figure 3. It can be shown that $A(a/b)^2 - B$ is a monotonically decreasing function of a/b with the root $a/b = 1$ and thus the conditions simplify to $a < b$ for the Case `I` and $a > b$ for the Case `II`. For $a = b$ the cases become identical. As per Fig. 3, the integration ranges are

$$\text{Case I}: \begin{cases} [C - \varepsilon - \frac{Aa^2}{4}, \frac{Bb^2}{4}] & \text{when} \quad C - \frac{Aa^2}{4} - \frac{Bb^2}{4} < \varepsilon < C - \frac{Aa^2}{4} \\ [0, \frac{Bb^2}{4}] & \text{when} \quad C - \frac{Aa^2}{4} \le \varepsilon \le C - \frac{Bb^2}{4} \\ [0, C-\varepsilon] & \text{when} \quad C - \frac{Bb^2}{4} < \varepsilon < C \end{cases} \tag{128}$$

$$\text{Case II}: \begin{cases} [C - \varepsilon - \frac{Aa^2}{4}, \frac{Bb^2}{4}] & \text{when} \quad C - \frac{Aa^2}{4} - \frac{Bb^2}{4} < \varepsilon < C - \frac{Bb^2}{4} \\ [C - \varepsilon - \frac{Aa^2}{4}, C-\varepsilon] & \text{when} \quad C - \frac{Bb^2}{4} \le \varepsilon \le C - \frac{Aa^2}{4} \\ [0, C-\varepsilon] & \text{when} \quad C - \frac{Aa^2}{4} < \varepsilon < C \end{cases} \tag{129}$$

Thus the energy shift distribution in the Case `I` ($a < b$) is found to be

$$\rho_{\Delta\mathcal{E}}(\varepsilon) = k \times \begin{cases} \frac{\pi}{2} - \mathrm{atan}\sqrt{\frac{4(C-\varepsilon)}{Bb^2} - 1} - \mathrm{atan}\sqrt{\frac{4(C-\varepsilon)}{Aa^2} - 1} & \text{when } -\frac{Aa^2+Bb^2}{6} < \varepsilon < -\frac{2Aa^2-Bb^2}{12} \\ \frac{\pi}{2} - \mathrm{atan}\sqrt{\frac{4(C-\varepsilon)}{Bb^2} - 1} & \text{when } -\frac{2Aa^2-Bb^2}{12} \le \varepsilon \le \frac{Aa^2-2Bb^2}{12} \\ \frac{\pi}{2} & \text{when } \frac{Aa^2-2Bb^2}{12} < \varepsilon < \frac{Aa^2+Bb^2}{12} \\ 0 & \text{otherwise} \end{cases} \tag{130}$$

where $k > 0$ is the proportionality constant. The distribution in the Case II ($a > b$) is identical to Eq. (130) provided that all Aa^2 are replaced with Bb^2 and vice versa.

Examples of energy shift distribution given by Eq. (130) are presented in Figure 8 of the main text for rectangular wafers with constant area but various side length ratios. As in the anisotropic circular case, distribution has a flat portion consisting of complete elliptical isocurves and a left-hand side tail caused by the isocurves cropped by the wafer edges in (see Fig. 7 of the main text).When $a \neq b$, the tails exhibit a non-differentiable kink due to the isocurves being cropped at different energy shifts along the minor and major axes. Keeping a/b constant, the width of the curve scales proportional to the surface area of the wafer or, equivalently put, to the second power of its linear dimensions and to good accuracy it is directly proportional to the Poisson ratio.

The energy resolution of due to transverse stretching can be estimated by calculating the standard deviation σ of Eq. (130). By integrating the first and second moments of the normalized distribution, we obtain

$$\begin{aligned}\sigma &= \frac{1}{6\sqrt{5}}\sqrt{A^2a^4 + B^2b^4} \\ &= \frac{\nu ab\mathcal{E}}{6gR^2}\sqrt{6 + 2\nu + \frac{115 + 2\nu - \nu^2}{20}M_1 + (1-\nu)M_2 + \frac{(1-\nu)^2}{20}M_3}\end{aligned} \tag{131}$$

where

$$M_k = \left(\frac{a^2}{b^2}\right)^k + \left(\frac{b^2}{a^2}\right)^k. \tag{132}$$

The FWHM compliant with the central limit theorem is obtained by multiplying σ by $2\sqrt{2\ln 2}$. The standard deviation of the energy shift distribution for various ν is plotted in the left panel of Fig. 4 as a function wafer side length ratio. It can be seen that regardless of ν, the standard deviation is maximised and thus the energy resolution of the wafer is the worst when $a/b = 1$ as already indicated by Fig. 8 of the main text.

Although not obvious from the expression, the square root term divided by g in Eq. (131) is found to depend rather weakly on ν (Fig. 4, right panel). Therefore in practice the exact relation can be approximated to the sufficient extent by the following, considerably simpler expression

$$\sigma \approx \frac{\nu ab\mathcal{E}}{12\sqrt{2}R^2}\frac{\sqrt{1+0.4M_1}}{1+M_1} \tag{133}$$

which is accurate within a few precent over the range $0 < \nu < 1$ being near exact for $\nu = 0.5$.

2.4. Anisotropic rectangular wafer

In principle the solution for the anisotropic rectangular wafer is obtained by following the same steps as for the anisotropic circular wafer, except for the fact that the integration domain is different. However, it turns out that even though an analytical solution exists, it is too complicated to be practical. Therefore the best approach to anisotropic crystal is to find the solution to the linear system numerically. However, the analytical solution simplifies problem slightly as it turns out that the coefficients $C_{30} = C_{03} = C_{21} = C_{12} = 0$. In addition, the Lagrange multiplier for the integrated contact force $\lambda_2 = 0$ which, in line with the derivations so far, allows us to omit that constraint from the energy minimization.[7] Thus we can reduce the number of unknowns to be solved from 14 down to 9. We now write the ansatz in the following form

$$\chi = C_{11}xy + \frac{1}{2}\left(C_{20}x^2 + C_{02}y^2\right) + 6C_{22}x^2y^2 + 4\left(C_{31}x^3y + C_{13}xy^3\right) + C_{40}x^4 + C_{04}y^4 \tag{134}$$

where the numerical prefactors are chosen to simplify the form of the linear system. Substituting the ansatz to Eqs. (32), we find the transverse stress tensor components

[7] An interesting question is whether the integrated contact force vanishes automatically in the minimization of $\mathcal{L} = \mathcal{F} + \lambda_1 f_c$, or does it happen *e.g.* for certain crystal symmetries. Intuitively one could expect the former, as the wafer is easiest to bend by applying a (relatively) weak force normal to the surface but showing this mathematically is out of the scope of this paper.

to be

$$\sigma_{xx} = C_{02} + 12C_{22}x^2 + 24C_{13}xy + 12C_{04}y^2 \tag{135}$$

$$\sigma_{yy} = C_{20} + 12C_{22}y^2 + 24C_{31}xy + 12C_{40}x^2 \tag{136}$$

$$\sigma_{xy} = -C_{11} - 12C_{31}x^2 - 24C_{22}xy - 12C_{13}y^2 \tag{137}$$

The toroidal minimization constraint [Eq. (47)] is now

$$f_c = 24(2S_{12} + S_{66})C_{22} - 48S_{26}C_{31} - 48S_{16}C_{13} + 24S_{22}C_{40} + 24S_{11}C_{04} + \frac{1}{R_1R_2} = 0 \tag{138}$$

The linear system to be minimized is presented in a matrix form Appendix F. After the numerical minimization, the components of the stretching tensor are obtained from Eqs. (135)–(137) and the components of the corresponding strain tensor from Eqs. (27)–(29) and (49)–(51). The contact force can be calculated from Eq. (45).

The predicted reflectivity curves from the anisotropic model are compared to the isotropic one for Si(008), Si(555) and Si(731) reflections in Figure 9 of the main text. In general, the isotropic model seems to follow its more intricate anisotropic counterpart rather well when Poisson's ratio used for the model is calculated by averaging the anisotropic Poisson's ratio over 2π in-plane *i.e.*

$$\overline{\nu} = -\frac{1}{2\pi}\int_0^{2\pi} d\phi \, \frac{S'_{13}}{S'_{11}} \tag{139}$$

where S'_{ij} are now taken to be functions of ϕ. Calculated $\overline{\nu}$ for selected reflections of Si and Ge are presented in Table 1. Unlike for the anisotropic circular crystal, the shape of the resolution curve do not seem to change considerably between different reflections even though their width varies. This is an indication that, as in the isotropic model, the shape of the resolution curve is largely determined by the aspect ratio of the wafer whereas Poisson's ratio scales its width.

However, the isotropic model fails to capture some details in the reflectivity curves, most notably the effect of the in-plane orientation of the crystal which for some reflec-

tions [*e.g.* Si(008)] can cause a significant effect to the resolution curve of the crystal. Nevertherless, as it is evident from Eqs. (135)–(137), the isocurves of the transverse stresses, and thus the strains as well, are elliptical in shape as they are in the isotropic case, although for some crystals and orientations the main axes of the ellipses may be inclined with respect to sides of the wafer, as seen for Si(731) in Fig. 9 of the main text.

For the investigated reflections, the isotropic model with in-plane averaged Poisson's ratio $\overline{\nu}$ appears to be a reasonable approximation to the anisotropic one at least for cubic systems. Further theoretical or computational work is needed to extrapolate the conclusion to other crystal systems.

Appendix A
List of Assumptions

This appendix lists the assumptions made in the derivation of theory and the special cases.

General theory

- Crystal wafer is arbitrarily shaped in the xy-plane, constant thickness in z
- No restrictions are put on the crystal symmetry
- The compliance matrix S is presented in the Voigt notation.
- Deformations of the wafer are in the scope of linear elasticity
- Crystal wafer is assumed to be thin *i.e.* its dimensions are much larger in the xy-plane than in the z-direction
- Bending radii of the toroidal deformation are assumed to be large compared to the xy-dimensions of the wafer

- Toroidal deformation can be approximated with paraboloidal surface (corollary to the previous assumption)
- Meridional bending radius is aligned with the x-axis and the sagittal one with the y-axis
- The pure bending solution with two acting torques can accurately produce the deformation field of an arbitrary paraboloidal displacement when the torques are allowed to rotate jointly in-plane
- External forces or body forces acting on the crystal are assumed to be negligible in comparison to the internal stresses
- Total strain tensor can be divided into two parts: the pure bending and stretching components
- Pure bending strain tensor components are linear in z (corollary to thin wafer assumption)
- Stretching strain tensor components are independent of z (corollary to thin wafer assumption)
- Total elastic deformation energy is the sum of pure bending energy and the stretching energy (corollary to previous two assumptions)
- Total stress and strain tensors can be found by minimizing the two aforementioned energy components separately
- Mechanical deformation energy is minimized under two constraints: the vertical (z) deflection of the wafer follows toroidal surface; and the integrated contact force between the wafer and the surface is zero
- The solution can be found by low-order polynomial expansion of the stress function χ
- Diffraction of X-rays is calculated from the Takagi-Taupin theory
- Diffraction takes place in xz-plane

- Components of the stretching strain tensor are (near) constant in the diffraction domain of a single ray
- Pure bending component of the strain seen by X-ray at any (x, y)-position of the wafer is the same
- Total diffraction curve of the full wafer can be calculated by convolving the pure bending Takagi-Taupin solution with the contribution of the stretching part (corollary to the previous two assumptions)

Important special cases

- Energy minimizations are made without the integrated contact force constraint; the validity of constraint is checked afterwards
- The Mitchell solution gives the full solution to the isotropic circular wafer
- Fourth-order expansion is sufficiently accurate for χ based on the isotropic circular case solution
- No additional boundary conditions for the stress or strain tensor are enforced
- Rectangular wafers are assumed to be aligned with the meridional and sagittal bending radii
- Rectangular symmetry is required of the χ ansatz for the isotropic rectangular wafer
- To approximate the anisotropic rectangular wafer with the isotropic model, Poisson's ratio is obtained by averaging the anisotropic Poisson's ratio in the xy-plane
- Strips of the strip-bent analyser can be approximated with masked rectangular wafers

Appendix B
Connection of vertical displacement and transverse stress

From Hooke's law, the transverse components of the strain relate to the stresses by

$$u_{xx} = S_{11}\sigma_{xx} + S_{12}\sigma_{yy} + S_{16}\sigma_{xy} \tag{140}$$

$$u_{yy} = S_{21}\sigma_{xx} + S_{22}\sigma_{yy} + S_{26}\sigma_{xy} \tag{141}$$

$$u_{xy} = \frac{S_{61}}{2}\sigma_{xx} + \frac{S_{62}}{2}\sigma_{yy} + \frac{S_{66}}{2}\sigma_{xy} \tag{142}$$

For large deflections, the strain tensor components are

$$u_{xx} = \frac{\partial u_x}{\partial x} + \frac{1}{2}\left(\frac{\partial \zeta}{\partial x}\right)^2 \tag{143}$$

$$u_{yy} = \frac{\partial u_y}{\partial y} + \frac{1}{2}\left(\frac{\partial \zeta}{\partial y}\right)^2 \tag{144}$$

$$u_{xy} = \frac{1}{2}\left(\frac{\partial u_x}{\partial y} + \frac{\partial u_y}{\partial x} + \frac{\partial \zeta}{\partial x}\frac{\partial \zeta}{\partial y}\right) \tag{145}$$

Substituting the former and stresses from Eq. (32) to Eqs. (140)–(142) we obtain

$$\frac{\partial u_x}{\partial x} + \frac{1}{2}\left(\frac{\partial \zeta}{\partial x}\right)^2 = S_{11}\frac{\partial^2 \chi}{\partial y^2} + S_{12}\frac{\partial^2 \chi}{\partial x^2} - S_{16}\frac{\partial^2 \chi}{\partial x \partial y} \tag{146}$$

$$\frac{\partial u_y}{\partial y} + \frac{1}{2}\left(\frac{\partial \zeta}{\partial y}\right)^2 = S_{21}\frac{\partial^2 \chi}{\partial y^2} + S_{22}\frac{\partial^2 \chi}{\partial x^2} - S_{26}\frac{\partial^2 \chi}{\partial x \partial y} \tag{147}$$

$$\frac{\partial u_x}{\partial y} + \frac{\partial u_y}{\partial x} + \frac{\partial \zeta}{\partial x}\frac{\partial \zeta}{\partial y} = S_{61}\frac{\partial^2 \chi}{\partial y^2} + S_{62}\frac{\partial^2 \chi}{\partial x^2} - S_{66}\frac{\partial^2 \chi}{\partial x \partial y} \tag{148}$$

By taking the partial derivatives $\partial^2/\partial y^2$, $\partial^2/\partial x^2$, and $-\partial^2/\partial x \partial y$ of Eqs. (140), (141), and (142), respectively, we find

$$\frac{\partial^3 u_x}{\partial x \partial y^2} + \frac{\partial \zeta}{\partial x}\frac{\partial^3 \zeta}{\partial x \partial y^2} + \left(\frac{\partial^2 \zeta}{\partial x \partial y}\right)^2 = S_{11}\frac{\partial^4 \chi}{\partial y^4} + S_{12}\frac{\partial^4 \chi}{\partial x^2 \partial y^2} - S_{16}\frac{\partial^4 \chi}{\partial x \partial y^3} \tag{149}$$

$$\frac{\partial^3 u_y}{\partial x^2 \partial y} + \frac{\partial \zeta}{\partial y}\frac{\partial^3 \zeta}{\partial x^2 \partial y} + \left(\frac{\partial^2 \zeta}{\partial x \partial y}\right)^2 = S_{21}\frac{\partial^4 \chi}{\partial x^2 \partial y^2} + S_{22}\frac{\partial^4 \chi}{\partial x^4} - S_{26}\frac{\partial^4 \chi}{\partial x^3 \partial y} \tag{150}$$

$$\begin{aligned} -\frac{\partial^3 u_x}{\partial x \partial y^2} - \frac{\partial^3 u_y}{\partial x^2 \partial y} - \frac{\partial^3 \zeta}{\partial x^2 \partial y}\frac{\partial \zeta}{\partial y} - \frac{\partial^3 \zeta}{\partial x \partial y^2}\frac{\partial \zeta}{\partial x} - \frac{\partial^2 \zeta}{\partial x^2}\frac{\partial^2 \zeta}{\partial y^2} - \left(\frac{\partial^2 \zeta}{\partial x \partial y}\right)^2 = \\ -S_{61}\frac{\partial^4 \chi}{\partial x \partial y^3} - S_{62}\frac{\partial^4 \chi}{\partial x^3 \partial y} + S_{66}\frac{\partial^4 \chi}{\partial x^2 \partial y^2} \end{aligned} \tag{151}$$

Summing up the equations above sidewise, we thus obtain

$$\mathcal{D}^4 \chi = \left(\frac{\partial^2 \zeta}{\partial x \partial y}\right)^2 - \frac{\partial^2 \zeta}{\partial x^2}\frac{\partial^2 \zeta}{\partial y^2}, \tag{152}$$

where the linear operator $\mathcal{D}^4$ is defined by

$$\mathcal{D}^4 \equiv S_{11}\frac{\partial^4}{\partial y^4} + (2S_{12} + S_{66})\frac{\partial^4}{\partial x^2 \partial y^2} + S_{22}\frac{\partial^4}{\partial x^4} - 2S_{16}\frac{\partial^4}{\partial x \partial y^3} - 2S_{26}\frac{\partial^4}{\partial x^3 \partial y} \qquad (153)$$

and simplified using the symmetry property $S_{ij} = S_{ji}$. Eq. (152) is an anisotropic generalization of Equation (14.7) in (Landau *et al.*, 1986)[p. 53], to which it reduces in the isotropic case.

Appendix C
Contact forces at the wafer–substrate interface

Consider a rectangular volume covering the wafer over its whole thickness d in z-direction but small in the transverse directions x and y. Due to the curved substrate, the surface of the wafer is only approximately aligned with the xy-plane and thus the total force acting on the volume element has a small component in z which has to be cancelled by the surface force P.

Let an edge of the volume parallel to z be located at (x, y). Now the normal force acting on the face defined by edges at (x, y) and $(x, y + \Delta y)$, where Δy is the side length of the volume in y-direction, is

$$F_{x,n} = -d \sin\phi_x \sigma_{xx} \Delta y \qquad (154)$$

where ϕ_x is the inclination of the wafer with respect to the xy-plane along x. The sign is a result of the outward normal of the face pointing in the negative x-direction. Since $\sin\phi_x \approx \partial\zeta/\partial x$, the normal force on the opposite face defined by the edges at $(x + \Delta x, y)$ and $(x + \Delta x, y + \Delta y)$, where Δx is the side length of the volume in x-direction, can be written up to the first order as

$$F'_{x,n} \approx -F_{x,n} + d\frac{\partial^2\zeta}{\partial x^2}\sigma_{xx}\Delta y \Delta x + d\frac{\partial\zeta}{\partial x}\frac{\partial\sigma_{xx}}{\partial x}\Delta y \Delta x. \qquad (155)$$

Performing the same steps for the shear force in the x-direction and summing all the

forces together, we find the total force due to the stress acting in x is

$$F_x = d\left(\frac{\partial^2\zeta}{\partial x^2}\sigma_{xx} + \frac{\partial\zeta}{\partial x}\frac{\partial\sigma_{xx}}{\partial x} + \frac{\partial^2\zeta}{\partial x\partial y}\sigma_{xy} + \frac{\partial\zeta}{\partial x}\frac{\partial\sigma_{xy}}{\partial y}\right)\Delta x\Delta y. \tag{156}$$

Analogously for the stress acting in the y-direction

$$F_y = d\left(\frac{\partial^2\zeta}{\partial y^2}\sigma_{yy} + \frac{\partial\zeta}{\partial y}\frac{\partial\sigma_{yy}}{\partial y} + \frac{\partial^2\zeta}{\partial x\partial y}\sigma_{xy} + \frac{\partial\zeta}{\partial y}\frac{\partial\sigma_{xy}}{\partial x}\right)\Delta x\Delta y. \tag{157}$$

Substituting the Airy stress function χ from Eq. (32), we find the total force in the z-direction per unit area to be

$$\frac{F_x + F_y}{\Delta x\Delta y} \approx d\left(\frac{\partial^2\zeta}{\partial x^2}\frac{\partial^2\chi}{\partial y^2} + \frac{\partial^2\zeta}{\partial y^2}\frac{\partial^2\chi}{\partial x^2} - 2\frac{\partial^2\zeta}{\partial x\partial y}\frac{\partial^2\chi}{\partial x\partial y}\right) \tag{158}$$

which becomes exact at the limit $\Delta x, \Delta y \to 0$. Substituting the toroidal displacement $\zeta(x,y) = x^2/2R_1 + y^2/2R_2$, we find that the compensating surface force per unit area at the wafer–substrate interface is

$$P = -d\left(\frac{1}{R_1}\frac{\partial^2\chi}{\partial y^2} + \frac{1}{R_2}\frac{\partial^2\chi}{\partial x^2}\right) = -d\left(\frac{\sigma_{xx}}{R_1} + \frac{\sigma_{yy}}{R_2}\right). \tag{159}$$

Since thicknesses of the crystal wafers are typically a few hundred micrometers and the bending radii are range from tens to hundreds of centimeters, we may conclude on the basis of the derived expression that the surface forces are indeed negligible compared to the internal stresses.

Appendix D
Minimization of $\mathcal{F}$ for an anisotropic circular wafer

The stretching energy $\mathcal{F}$ is minimized with the toroidal bending constraint $f_c = 0$ by finding the minimum of $\mathcal{L} = \mathcal{F} + \lambda_1 f_c + \lambda_2 F_c$ by solving the linear system given by Eq. (48). It turns out that the contact force constraint F_c can be omitted in the minimization as it is implicitly fulfilled by the solution obtained without it. With the

toroidal bending constraint f_c given by Eq. (96), the linear system becomes

$$\partial_{11}\mathcal{F} = 0, \quad \partial_{20}\mathcal{F} = \partial_{02}\mathcal{F} = 0, \quad \partial_{21}\mathcal{F} = \partial_{12}\mathcal{F} = 0, \quad \partial_{30}\mathcal{F} = \partial_{03}\mathcal{F} = 0$$
$$\partial_{31}\mathcal{F} - 2S_{26}\lambda = 0, \quad \partial_{13}\mathcal{F} - 2S_{16}\lambda = 0, \quad \partial_{40}\mathcal{F} + S_{22}\lambda = 0, \quad \partial_{04}\mathcal{F} + S_{11}\lambda = 0$$
$$\partial_{22}\mathcal{F} + (2S_{22} + S_{66})\lambda = 0 \quad f_c = 0 \tag{160}$$

where the shorthand $\partial_k\mathcal{F} \equiv \partial\mathcal{F}/\partial C_k$ has been used. By expressing σ_{ij} in Eqs. (93)–(95) in polar coordinates, substituting them to Eq. (39), and carrying out the integration over a circular domain with the diameter L, we obtain

$$\partial_{11}\mathcal{F} = \frac{\pi d L^4}{64}\left[-(S_{16} + S_{26})\, C_{22} - S_{16}C_{04} - S_{26}C_{40} + S_{66}\,(C_{31} + C_{13}) - \frac{16}{L^2}\,(S_{16}C_{02} + S_{26}C_{20} - S_{66}C_{11}) \right] \tag{161}$$

$$\partial_{20}\mathcal{F} = \frac{\pi d L^4}{64}\left[(S_{12} + S_{22})\, C_{22} + S_{12}C_{04} + S_{22}C_{40} - S_{26}\,(C_{31} + C_{13}) + \frac{16}{L^2}\,[S_{12}C_{02} + S_{22}C_{20} - S_{26}C_{11}] \right] \tag{162}$$

$$\partial_{02}\mathcal{F} = \frac{\pi d L^4}{64}\left[(S_{11} + S_{12})\, C_{22} + S_{11}C_{04} + S_{12}C_{40} - S_{16}\,(C_{31} + C_{13}) + \frac{16}{L^2}\,(S_{11}C_{02} + S_{12}C_{20} - S_{16}C_{11}) \right] \tag{163}$$

$$\partial_{21}\mathcal{F} = \frac{\pi d L^4}{64}\left[(S_{22} + S_{66})\, C_{21} - (S_{16} + S_{26})\, C_{12} + S_{12}C_{03} - S_{26}C_{30} \right] \tag{164}$$

$$\partial_{12}\mathcal{F} = \frac{\pi d L^4}{64}\left[(S_{11} + S_{66})\, C_{12} - (S_{16} + S_{26})\, C_{21} + S_{12}C_{30} - S_{16}C_{03} \right] \tag{165}$$

$$\partial_{22}\mathcal{F} = \frac{\pi d L^4}{64}\left[(S_{11} + S_{12})\, C_{02} + (S_{12} + S_{22})\, C_{20} - (S_{16} + S_{26})\, C_{11} + \frac{L^2}{24}\left[(3S_{11} + 2S_{12} + 3S_{22} + 4S_{66})\, C_{22} - (3S_{16} + 5S_{26})\, C_{31} - (5S_{16} + 3S_{26})\, C_{13} + (3S_{12} + S_{22})\, C_{40} + (S_{11} + 3S_{12})\, C_{04} \right] \right] \tag{166}$$

$$\partial_{31}\mathcal{F} = \frac{\pi d L^4}{64}\Bigg[S_{66}C_{11} - S_{16}C_{02} - S_{26}C_{20} - \frac{L^2}{24}\Big[(3S_{16}+5S_{26})\,C_{22} - (4S_{12}+S_{66})\,C_{13} - (4S_{22}+3S_{66})\,C_{31} - S_{16}C_{04} - 3S_{26}C_{40}\Big]\Bigg] \quad (167)$$

$$\partial_{13}\mathcal{F} = \frac{\pi d L^4}{64}\Bigg[S_{66}C_{11} - S_{16}C_{02} - S_{26}C_{20} - \frac{L^2}{24}\Big[(3S_{26}+5S_{16})\,C_{22} - (4S_{12}+S_{66})\,C_{31} - (4S_{11}+3S_{66})\,C_{13} - S_{26}C_{04} - 3S_{16}C_{40}\Big]\Bigg] \quad (168)$$

$$\partial_{30}\mathcal{F} = \frac{\pi d L^4}{64}\Big[S_{12}C_{12} - S_{26}C_{21} + S_{22}C_{30}\Big] \quad (169)$$

$$\partial_{03}\mathcal{F} = \frac{\pi d L^4}{64}\Big[S_{12}C_{21} - S_{16}C_{12} + S_{11}C_{03}\Big] \quad (170)$$

$$\partial_{40}\mathcal{F} = \frac{\pi d L^4}{64}\Bigg[S_{12}C_{02} + S_{22}C_{20} - S_{26}C_{11} + \frac{L^2}{24}\Big[(3S_{12}+S_{22})\,C_{22} - S_{26}\,(3C_{31}+C_{13}) + S_{12}C_{04} + 3S_{22}C_{40}\Big]\Bigg] \quad (171)$$

$$\partial_{04}\mathcal{F} = \frac{\pi d L^4}{64}\Bigg[S_{11}C_{02} + S_{12}C_{20} - S_{16}C_{11} + \frac{L^2}{24}\Big[(S_{11}+3S_{12})\,C_{22} - S_{16}\,(3C_{13}+C_{31}) + S_{12}C_{40} + 3S_{11}C_{04}\Big]\Bigg] \quad (172)$$

Substituting the found derivatives to Eq. (160), the solution to the system is

$$\begin{array}{llll} C_{11} = 0 & C_{20} = C_{02} = \dfrac{E'L^2}{64R^2} & C_{40} = C_{04} = -\dfrac{3E'}{16R^2} & C_{22} = -\dfrac{E'}{16R^2} \\ C_{30} = C_{03} = 0 & C_{21} = C_{12} = 0 & C_{31} = C_{13} = 0 & \lambda = \dfrac{\pi d E' L^6}{6144R^2} \end{array} \quad (173)$$

where $R^2 = R_1R_2$ is the product of bending radii and

$$E' = \frac{8}{3(S_{11}+S_{22}) + 2S_{12} + S_{66}}. \quad (174)$$

Appendix E
Minimization of $\mathcal{F}$ for an isotropic rectangular wafer

The stretching energy $\mathcal{F}$ is minimized by finding the coefficients $\{C_{ij}, \lambda_1, \lambda_2\}$ which

minimize $\mathcal{L} = \mathcal{F} + \lambda_1 f_c + \lambda_2 F_c$ by solving the linear system given by Eq. (48). The constraint f_c is obtained by the requirement that χ solves Eq.(44) *i.e.*

$$f_c = \nabla^4\chi + \frac{E}{R^2} = 2C_{40} + 4C_{22} + 2C_{04} + \frac{E}{R^2} = 0, \tag{175}$$

where $R^2 = R_1R_2$ is the product of bending radii. Therefore the equations composing the linear system to be solved are

$$\partial_{20}\mathcal{F} = 0, \qquad \partial_{02}\mathcal{F} = 0, \qquad \partial_{40}\mathcal{F} + 2\lambda = 0,$$

$$\partial_{04}\mathcal{F} + 2\lambda = 0, \qquad \partial_{22}\mathcal{F} + 4\lambda = 0, \qquad f_c = 0. \tag{176}$$

Substituting the stretching stress tensor components given by Eq. (118) into the expression of partial derivatives Eq. (40) and carrying out the integration over rectangular domain with linear dimensions a and b in x- and y-directions, respectively, we thus obtain

$$\partial_{20}\mathcal{F} = \frac{abd}{E}\left[C_{20} - \nu C_{02} + (C_{40} - \nu C_{22})\frac{a^2}{12} + (C_{22} - \nu C_{04})\frac{b^2}{12}\right] \tag{177}$$

$$\partial_{02}\mathcal{F} = \frac{abd}{E}\left[C_{02} - \nu C_{20} + (C_{22} - \nu C_{40})\frac{a^2}{12} + (C_{04} - \nu C_{22})\frac{b^2}{12}\right] \tag{178}$$

$$\partial_{04}F = \frac{ab^3d}{12E}\left[C_{02} - \nu C_{20} + (C_{22} - \nu C_{40})\frac{a^2}{12} + 3(C_{04} - \nu C_{22})\frac{b^2}{20}\right] \tag{179}$$

$$\partial_{40}F = \frac{a^3bd}{12E}\left[C_{20} - \nu C_{02} + 3(C_{40} - \nu C_{22})\frac{a^2}{20} + (C_{22} - \nu C_{04})\frac{b^2}{12}\right] \tag{180}$$

$$\begin{aligned}\partial_{22}\mathcal{F} = \frac{abd}{12E}\Bigg[&(C_{02} - \nu C_{20})a^2 + (C_{20} - \nu C_{02})b^2 + 3(C_{22} - \nu C_{40})\frac{a^4}{20} \\ &+ \Big[C_{04} + C_{40} + (8 + 6\nu)C_{22}\Big]\frac{a^2b^2}{12} + 3(C_{22} - \nu C_{04})\frac{b^4}{20}\Bigg]\end{aligned} \tag{181}$$

Substituting the calculated derivatives to Eq. (176), the solution to the system is

$$C_{20} = \frac{E}{24gR^2}\left[(1+\nu)a^2 + 12b^2 + (1-\nu)\frac{b^4}{a^2}\right], \; C_{40} = -\frac{E}{2gR^2}\left[1 + \nu + 10\frac{b^2}{a^2} + (1-\nu)\frac{b^4}{a^4}\right],$$

$$C_{02} = \frac{E}{24gR^2}\left[(1+\nu)b^2 + 12a^2 + (1-\nu)\frac{a^4}{b^2}\right], \; C_{04} = -\frac{E}{2gR^2}\left[1 + \nu + 10\frac{a^2}{b^2} + (1-\nu)\frac{a^4}{b^4}\right],$$

$$C_{22} = -\frac{E}{gR^2}, \qquad \lambda = \frac{d}{720gR^2}\left[(1-\nu)(a^5b + ab^5) + 10a^3b^3\right] \tag{182}$$

where

$$g = 8 + 10\left(\frac{a^2}{b^2} + \frac{b^2}{a^2}\right) + (1-\nu)\left(\frac{a^2}{b^2} - \frac{b^2}{a^2}\right)^2 \tag{183}$$

Appendix F
Minimization of $\mathcal{F}$ for an anisotropic rectangular wafer

The stretching energy $\mathcal{F}$ is minimized with the toroidal bending constraint $f_c = 0$ by finding the minimum of $\mathcal{L} = \mathcal{F} + \lambda_1 f_c$ by solving the linear system given by Eq. (48). Using the ansatz from Eq. (134) for χ, the constraint from Eq. (138), and rewriting the Lagrange multiplier $\lambda_1 \to \lambda_1 abd/120$, we may reformulate the problem as solving the matrix equation $\Lambda\mathbf{C} = \mathbf{b}$ in terms of $\mathbf{C}$ where

$$\mathbf{C} = \begin{bmatrix} C_{11} & C_{20} & C_{02} & C_{22} & C_{31} & C_{13} & C_{40} & C_{04} & \lambda_1 \end{bmatrix}^{\mathrm{T}}, \tag{184}$$

$$\mathbf{b} = \begin{bmatrix} 0 & 0 & 0 & 0 & 0 & 0 & 0 & 0 & -(24R_1R_2)^{-1} \end{bmatrix}^{\mathrm{T}}, \tag{185}$$

and

$$\Lambda = \begin{bmatrix} S_{66} & -S_{26} & -S_{16} & \Lambda_{14} & S_{66}a^2 & S_{66}b^2 & -S_{26}a^2 & -S_{16}b^2 & 0 \\ -S_{26} & S_{22} & S_{12} & \Lambda_{24} & -S_{26}a^2 & -S_{26}b^2 & S_{22}a^2 & S_{12}b^2 & 0 \\ -S_{16} & S_{12} & S_{11} & \Lambda_{34} & -S_{16}a^2 & -S_{16}b^2 & S_{12}a^2 & S_{11}b^2 & 0 \\ \Lambda_{41} & \Lambda_{42} & \Lambda_{43} & \Lambda_{44} & \Lambda_{45} & \Lambda_{46} & \Lambda_{47} & \Lambda_{48} & \Lambda_{49} \\ 5S_{66}a^2 & -5S_{26}a^2 & -5S_{16}a^2 & \Lambda_{54} & \Lambda_{55} & \Lambda_{56} & -9S_{26}a^4 & -5S_{16}a^2b^2 & -2S_{26} \\ 5S_{66}b^2 & -5S_{26}b^2 & -5S_{16}b^2 & \Lambda_{64} & \Lambda_{65} & \Lambda_{66} & -5S_{26}a^2b^2 & -9S_{16}b^4 & -2S_{16} \\ -5S_{26}a^2 & 5S_{22}a^2 & 5S_{12}a^2 & \Lambda_{74} & -9S_{26}a^4 & -5S_{26}a^2b^2 & 9S_{22}a^4 & 5S_{12}a^2b^2 & S_{22} \\ -5S_{16}b^2 & 5S_{12}b^2 & 5S_{11}b^2 & \Lambda_{84} & -5S_{16}a^2b^2 & -9S_{16}b^4 & 5S_{12}a^2b^2 & 9S_{11}b^4 & S_{11} \\ 0 & 0 & 0 & \Lambda_{94} & -2S_{26} & -2S_{16} & S_{22} & S_{11} & 0 \end{bmatrix} \tag{186}$$

with

$$\Lambda_{14} = -S_{16}a^2 - S_{26}b^2 \qquad \Lambda_{24} = S_{12}a^2 + S_{22}b^2$$

$$\Lambda_{34} = S_{11}a^2 + S_{12}b^2 \qquad \Lambda_{41} = -5S_{16}a^2 - 5S_{26}b^2$$

$$\Lambda_{42} = 5S_{12}a^2 + 5S_{22}b^2 \qquad \Lambda_{43} = 5S_{11}a^2 + 5S_{12}b^2$$

$$\Lambda_{44} = 9S_{11}a^4 + 9S_{22}b^4 + 10(S_{12} + 2S_{66})a^2b^2 \qquad \Lambda_{45} = -9S_{16}a^4 - 25S_{26}a^2b^2$$

$$\Lambda_{46} = -25S_{16}a^2b^2 - 9S_{26}b^4 \qquad \Lambda_{47} = 9S_{12}a^4 + 5S_{22}a^2b^2$$

$$\Lambda_{48} = 5S_{11}a^2b^2 + 9S_{12}b^4 \qquad \Lambda_{49} = 2S_{12} + S_{66}$$

$$\Lambda_{54} = -9S_{16}a^4 - 25S_{26}a^2b^2 \qquad \Lambda_{55} = 9S_{66}a^4 + 20S_{22}a^2b^2$$

$$\Lambda_{56} = 5(4S_{12} + S_{66})a^2b^2 \qquad \Lambda_{64} = -25S_{16}a^2b^2 - 9S_{26}b^4$$

$$\Lambda_{65} = 5(4S_{12} + S_{66})a^2b^2 \qquad \Lambda_{66} = 20S_{11}a^2b^2 + 9S_{66}b^4$$

$$\Lambda_{74} = 9S_{12}a^4 + 5S_{22}a^2b^2 \qquad \Lambda_{84} = 5S_{11}a^2b^2 + 9S_{12}b^4$$

$$\Lambda_{94} = 2S_{12} + S_{66}$$

Appendix G
Johann error

Consider a spherically bent crystal wafer with the meridional and sagittal bending radii R_1 and R_2, respectively. The surface of the spherical Johann-type analyser is approximately given by the constraint

$$f(x, y, z) = \frac{x^2}{2R_1} + \frac{y^2}{2R_2} - z = 0, \tag{187}$$

where R is the bending radius. Let

$$\mathbf{n} = -\nabla f = -\frac{x}{R_1}\hat{\mathbf{x}} - \frac{y}{R_2}\hat{\mathbf{y}} + \hat{\mathbf{z}}. \tag{188}$$

The surface normal vector field is thus $\hat{\mathbf{n}} = \mathbf{n}/n$, where

$$n = \sqrt{1 + \frac{x^2}{R_1^2} + \frac{y^2}{R_2^2}} \tag{189}$$

Let us denote the distance from the source to the point (x, y, z) on the crystal surface by the vector $\mathbf{R}$. According to Figure 5, we find that $\mathbf{R} = \mathbf{r} - \mathbf{r}'$, where $\mathbf{r}'$ is the position vector of the source and $\mathbf{r}$ is the position vector of the surface point in question. From Figure 5 we also see that

$$\mathbf{r}' = \rho \cos\delta \hat{\mathbf{x}} + \rho(1 + \sin\delta)\hat{\mathbf{z}}. \tag{190}$$

Since $\pi = \delta + \pi/2 + 2\gamma$ and $\gamma = \pi/2 - \theta$, we find that $\delta = 2\theta - \pi/2$. Thus

$$\mathbf{r}' = \rho \sin 2\theta \hat{\mathbf{x}} + \rho(1 - \cos 2\theta)\hat{\mathbf{z}}. \tag{191}$$

Therefore

$$\mathbf{R} = (x - \rho\sin 2\theta)\hat{\mathbf{x}} + y\hat{\mathbf{y}} - \left(\rho(1-\cos 2\theta) - \frac{x^2}{2R_1} - \frac{y^2}{2R_2}\right)\hat{\mathbf{z}} \tag{192}$$

$$\begin{aligned}
\Rightarrow |\mathbf{R}|^2 &= (x - \rho\sin 2\theta)^2 + y^2 + \left(\rho(1-\cos 2\theta) - \frac{x^2}{2R_1} - \frac{y^2}{2R_2}\right)^2 \\
&= \frac{1}{2}\left(x^2 + \frac{R_1}{R_2}y^2 - R_1^2\right)\cos 2\theta - xR_1\sin 2\theta \\
&\quad + \frac{1}{2}\left[x^2 + \left(2 - \frac{R_1}{R_2}\right)y^2 + R_1^2\right] + \left(\frac{x^2}{2R_1} + \frac{y^2}{2R_2}\right)^2
\end{aligned} \tag{193}$$

where the fact that the Rowland circle radius ρ is half the meridional bending radius R_1. Since $\cos 2\theta = 1 - 2\sin^2\theta$ and $\sin 2\theta = 2\sin\theta\cos\theta$, we get

$$\begin{aligned}
|\mathbf{R}|^2 &= R_1^2\sin^2\theta\Bigg[1 + \frac{(R_2 - R_1)y^2}{R_2R_1^2\sin^2\theta} - \frac{2x\cot\theta}{R_1} \\
&\qquad + \left(\frac{x^2}{R_1^2} + \frac{y^2}{R_1R_2}\right)\cot^2\theta + \frac{1}{4\sin^2\theta}\left(\frac{x^2}{R_1^2} + \frac{y^2}{R_1R_2}\right)^2\Bigg] \\
\Rightarrow \frac{1}{|\mathbf{R}|} &= \frac{1}{R_1\sin\theta}\Bigg[1 + \frac{(R_2 - R_1)y^2}{R_2R_1^2\sin^2\theta} - \frac{2x\cot\theta}{R_1} \\
&\qquad + \left(\frac{x^2}{R_1^2} + \frac{y^2}{R_1R_2}\right)\cot^2\theta + \frac{1}{4\sin^2\theta}\left(\frac{x^2}{R_1^2} + \frac{y^2}{R_1R_2}\right)^2\Bigg]^{-1/2}
\end{aligned} \tag{194}$$

The cosine of angle α is now given by

$$\cos\alpha = \frac{\hat{\mathbf{n}}\cdot\mathbf{R}}{|\mathbf{R}|} = \frac{\mathbf{n}\cdot\mathbf{R}}{n|\mathbf{R}|}. \tag{195}$$

Since

$$\mathbf{n} \cdot \mathbf{R} = -\frac{x^2}{2R_1} - \frac{y^2}{2R_2} + x \sin\theta \cos\theta - R_1 \sin^2\theta \tag{196}$$

we find that

$$\cos\alpha = -\sin\theta \left[1 - \frac{x}{R_1}\cot\theta + \frac{1}{2\sin^2\theta}\left(\frac{x^2}{R_1^2} + \frac{y^2}{R_1 R_2}\right)\right]\left(1 + \frac{x^2}{R_1^2} + \frac{y^2}{R_2^2}\right)^{-1/2}$$
$$\times \left[1 - \frac{2x\cot\theta}{R_1} - \frac{(R_1 - R_2)y^2}{R_2 R_1^2 \sin^2\theta} + \left(\frac{x^2}{R_1^2} + \frac{y^2}{R_1 R_2}\right)\cot^2\theta + \frac{1}{4\sin^2\theta}\left(\frac{x^2}{R_1^2} + \frac{y^2}{R_1 R_2}\right)^2\right]^{-1/2}. \tag{197}$$

Since x/R and y/R are small, we may expand $\cos\alpha$ as their series and retain only the terms up to the second order. Doing so we find

$$\cos\alpha \approx -\sin\theta - \frac{x^2}{2R_1^2}\frac{\cos^2\theta}{\sin\theta} + \frac{(R_1 - R_2)(R_1 \sin^2\theta - R_2)}{2R_1 R_2 \sin\theta} y^2. \tag{198}$$

From Figure 5 we see that $\alpha + \beta = \pi$ and $\theta' + \beta = \pi/2$. Thus $\alpha = \pi/2 + \theta' \Rightarrow \cos\alpha = -\sin\theta'$ and

$$\sin\theta' = \sin\theta + \frac{x^2}{2R_1^2}\frac{\cos^2\theta}{\sin\theta} - \frac{(R_1 - R_2)(R_1 \sin^2\theta - R_2)}{2R_1 R_2 \sin\theta} y^2. \tag{199}$$

By writing $\theta' = \theta + \Delta\theta$ and taking the first-order approximation $\sin\theta' \approx \sin\theta + \cos\theta\Delta\theta$, we find by comparing to Eq. (199) that

$$\Delta\theta = \frac{x^2}{2R_1^2}\cot\theta - \frac{(R_1 - R_2)(R_1 \sin^2\theta - R_2)}{2R_1 R_2 \sin\theta\cos\theta} y^2 \tag{200}$$

Note that since Eq. (200) is based on the first-order approximation of $\sin x$, it ceases to be valid near $\theta = \pi/2$ if $R_1 \neq R_2$.

Alternatively, given in terms of energy the Johann error is

$$\Delta\mathcal{E} = \frac{hc}{2d\sin\theta'} - \frac{hc}{2d\sin\theta} \approx -\frac{x^2}{2R_1^2}\mathcal{E}\cot^2\theta + \frac{(R_1 - R_2)(R_1 \sin^2\theta - R_2)}{2R_1 R_2 \sin^2\theta}\mathcal{E}y^2, \tag{201}$$

where $\mathcal{E} = hc/2d\sin\theta$. Unlike Eq. (200), Eq. (201) is also valid at $\theta = \pi/2$ since we do not expand $\sin x$ with respect to its argument.

References

Amenzade, Y. A. (1979). *Theory of elasticity*. Mir Publishers Moscow. English translation.

Chukhovskii, F. N., Chang, W. Z. & Förster, E. (1994). *Journal of Applied Crystallography*, **27**(6), 971–979. Doi:10.1107/s002188989400676x.

Gronkowski, J. (1991). *Physics Reports*, **206**(1), 1–41. Doi:10.1016/0370-1573(91)90086-2.

Honkanen, A.-P., Monaco, G. & Huotari, S. (2016). *Journal of Applied Crystallography*, **49**(4), 1284–1289. Doi:10.1107/s1600576716010402.

Honkanen, A.-P., Verbeni, R., Simonelli, L., Sala, M. M., Monaco, G. & Huotari, S. (2014). *Journal of Synchrotron Radiation*, **21**(1), 104–110. Doi:10.1107/S160057751302242X.

Huotari, S., Sahle, C. J., Henriquet, C., Al-Zein, A., Martel, K., Simonelli, L., Verbeni, R., Gonzalez, H., Lagier, M.-C., Ponchut, C., Sala, M. M., Krisch, M. & Monaco, G. (2017). *Journal of Synchrotron Radiation*, **24**(2), 521–530. Doi:10.1107/s1600577516020579.

Johann, H. H. (1931). *Zeitschrift für Physik*, **69**(3-4), 185–206. Doi:10.1007/bf01798121.

Landau, L. D., Pitaevskii, L. P., Kosevich, A. M. & Lifshitz, E. M. (1986). *Theory Of Elasticity*, vol. 7 of *Course of Theoretical Physics*. Butterworth-Heinemann, 3rd ed.

Lide, D. L. (ed.) (2001). *CRC Handbook of Chemistry and Physics*. CRC Press, 82nd ed.

Michell, J. H. (1899). *Proceedings of the London Mathematical Society*, **31**(1), 100–124. Doi:10.1112/plms/s1-31.1.100.

Rovezzi, M., Lapras, C., Manceau, A., Glatzel, P. & Verbeni, R. (2017). *Review of Scientific Instruments*, **88**(1), 013108. Doi:10.1063/1.4974100.

Takagi, S. (1962). *Acta Crystallographica*, **15**(12), 1311–1312. Doi:10.1107/s0365110x62003473.

Takagi, S. (1969). *Journal of the Physical Society of Japan*, **26**(5), 1239–1253. Doi:10.1143/jpsj.26.1239.

Taupin, D. (1964). *Bulletin de la Société française de Minéralogie et de Cristallographie*, **87**(4), 469–511. Doi:10.3406/bulmi.1964.5769.

Verbeni, R., Kocsis, M., Huotari, S., Krisch, M., Monaco, G., Sette, F. & Vanko, G. (2005). *Journal of Physics and Chemistry of Solids*, **66**(12), 2299–2305. Doi:10.1016/j.jpcs.2005.09.079.

Verbeni, R., Pylkkänen, T., Huotari, S., Simonelli, L., Vankó, G., Martel, K., Henriquet, C. & Monaco, G. (2009). *Journal of Synchrotron Radiation*, **16**(4), 469–476. Doi:10.1107/s090904950901886x.

		Si				Ge			
(hkl)	$\phi_{\max}$	E' (GPa)	ν'	$\overline{\nu}$	K	E' (GPa)	ν'	$\overline{\nu}$	K
(100)	–	147.14	0.3146	0.3173	0	116.84	0.3129	0.3162	0
(110)	$[1\bar{1}0]$	163.06	0.2043	0.1978	0.7061	131.15	0.1879	0.1793	0.8692
(111)	–	169.16	0.1621	0.1621	0	136.74	0.1391	0.1391	0
(210)	$[1\bar{2}0]$	156.94	0.2467	0.2456	0.3603	125.62	0.2366	0.2348	0.4237
(211)	$[\bar{1}11]$	163.06	0.2043	0.2041	0.2354	131.15	0.1879	0.1874	0.2897
(221)	$[1\bar{1}0]$	166.39	0.1812	0.1788	0.4814	134.20	0.1613	0.1580	0.6139
(311)	$[\bar{2}33]$	156.75	0.2480	0.2490	0.1479	125.44	0.2378	0.2389	0.1737
(321)	$[\bar{8},11,2]$*	163.06	0.2043	0.2023	0.4297	131.15	0.1879	0.1851	0.5289
(331)	$[1\bar{1}0]$	164.79	0.1924	0.1880	0.6047	132.73	0.1741	0.1684	0.7572
(511)	$[\bar{2}55]$	151.27	0.2860	0.2880	0.0616	120.52	0.2807	0.2833	0.0704
(531)	$[\overline{32},51,6]$*	160.38	0.2229	0.2207	0.4334	128.72	0.2091	0.2063	0.5217
(533)	$[\bar{6}55]$	165.73	0.1859	0.1854	0.2458	133.59	0.1666	0.1659	0.3110
(551)	$[1\bar{1}0]$	163.73	0.1997	0.1940	0.6696	131.77	0.1825	0.1751	0.8294
(553)	$[1\bar{1}0]$	167.32	0.1748	0.1733	0.3892	135.05	0.1539	0.1518	0.5022
(731)	$[\bar{9},20,3]$*	155.86	0.2542	0.2544	0.2578	124.64	0.2448	0.2450	0.3012
(953)	$[\overline{20},31,9]$*	161.33	0.2163	0.2155	0.3211	129.58	0.2016	0.2004	0.3893

Table 1. *Derived elastic quantities for selected* (hkl) *normal to the wafer surface of silicon and germanium.* $\phi_{\max}$ *is the in-plane direction of steepest gradient of* u_{zz} *which are valid for all cubic systems (directions marked with an asterisk are approximate integer Miller indices).* E', ν' *and* K *are the effective Young modulus [Eq.* (98)*], effective Poisson ratio [Eq.* (115)*] and eccentricity factor [Eq.* (116)*], respectively, for the anisotropic circular wafer.* $\overline{\nu}$ *is Poisson ratio averaged over* 2π *angle in-plane. The values of elastic matrix elements for Si and Ge are according to (Lide, 2001).*

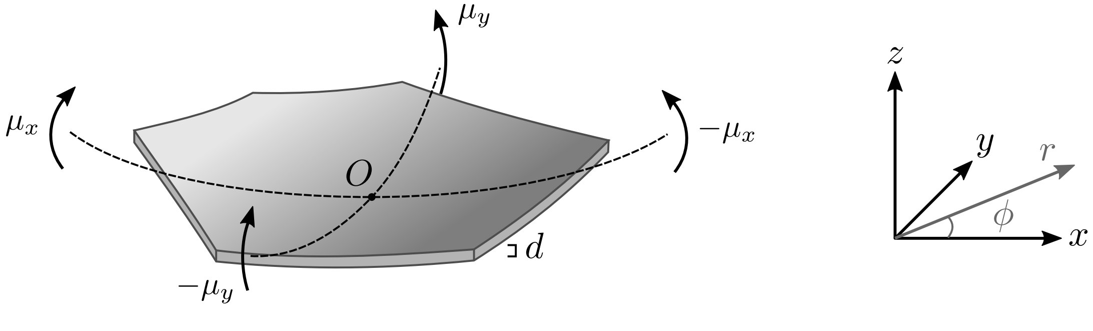

Fig. 1. Nomenclature for a toroidally bent wafer. The thickness of the wafer is d. The origin O of the Cartesian coordinate system (x, y, z) and the polar coordinates (r, ϕ) is located at the midplane of the crystal in the z-direction. Two orthogonal torques μ_x and μ_y cause the bending of the wafer.

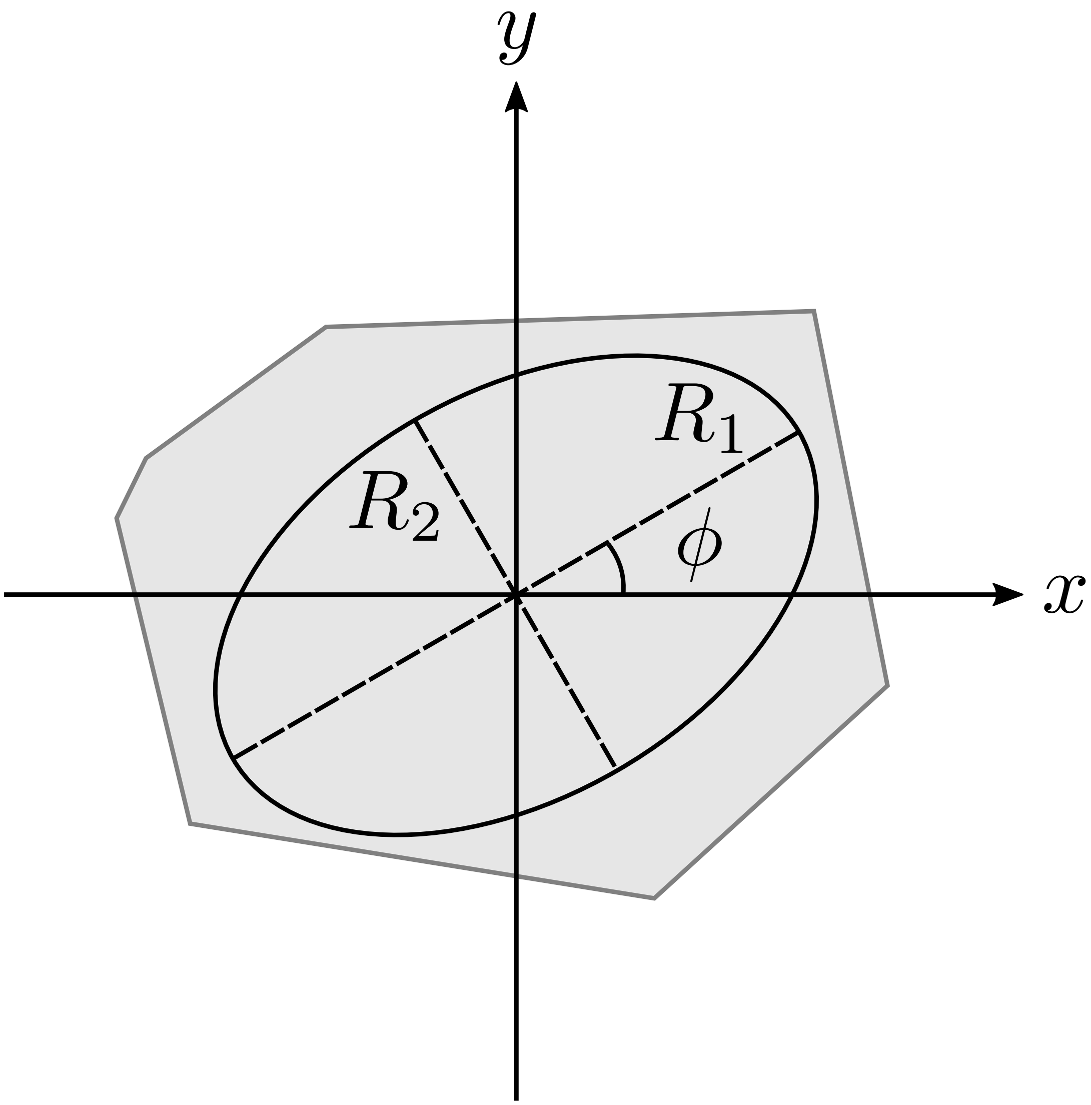

Fig. 2. Nomenclature for a toroidally bent wafer. The thickness of the wafer is d. The origin O of the Cartesian coordinate system (x, y, z) and the polar coordinates (r, ϕ) is located at the midplane of the crystal in the z-direction. Two orthogonal torques μ_x and μ_y cause the bending of the wafer.

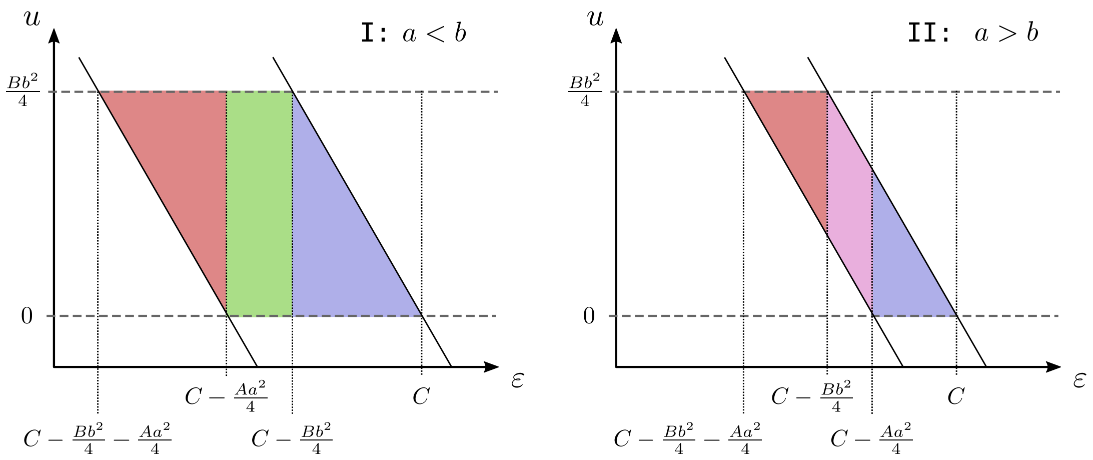

Fig. 3. Restrictions to the integration range in terms of u imposed by the condition $C - \varepsilon - Aa^2/4 < u < C - \varepsilon$. The valid integration range presented as colored areas depends linearly on ϵ in a piecewise manner and is divided into two cases based on whether $Aa^2 > Bb^2$ or $Aa^2 < Bb^2$. Equivalently, these conditions can be restated in a respective manner as $a < b$ and $a > b$.

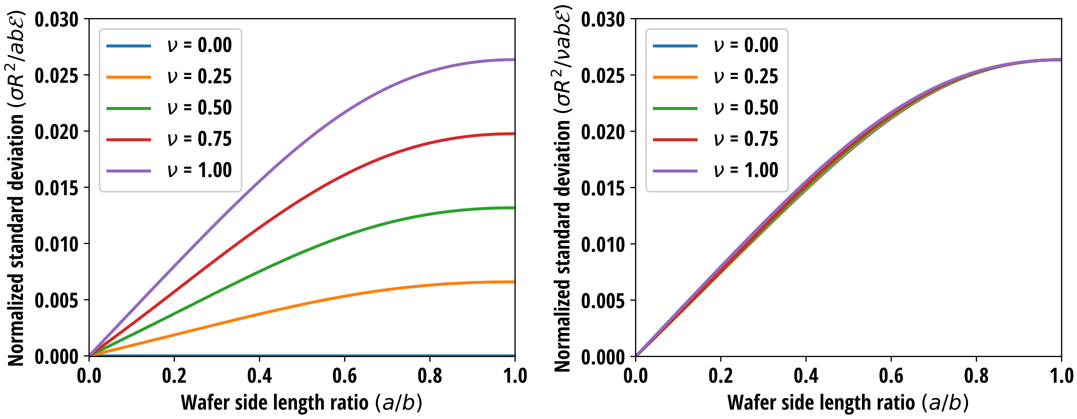

Fig. 4. *Left:* Normalized standard deviation of the energy shift distribution of isotropic rectangular crystal wafer according to Eq. (131) for various Poisson's ratios ν. The standard deviation is normalized to the wafer surface area, bending radius and the energy of the incident photons. *Right:* The curves presented on the left panel divided by ν demonstrating the relative insensitivity of the standard deviation σ to the value of Poisson's ratio ν apart from scaling.

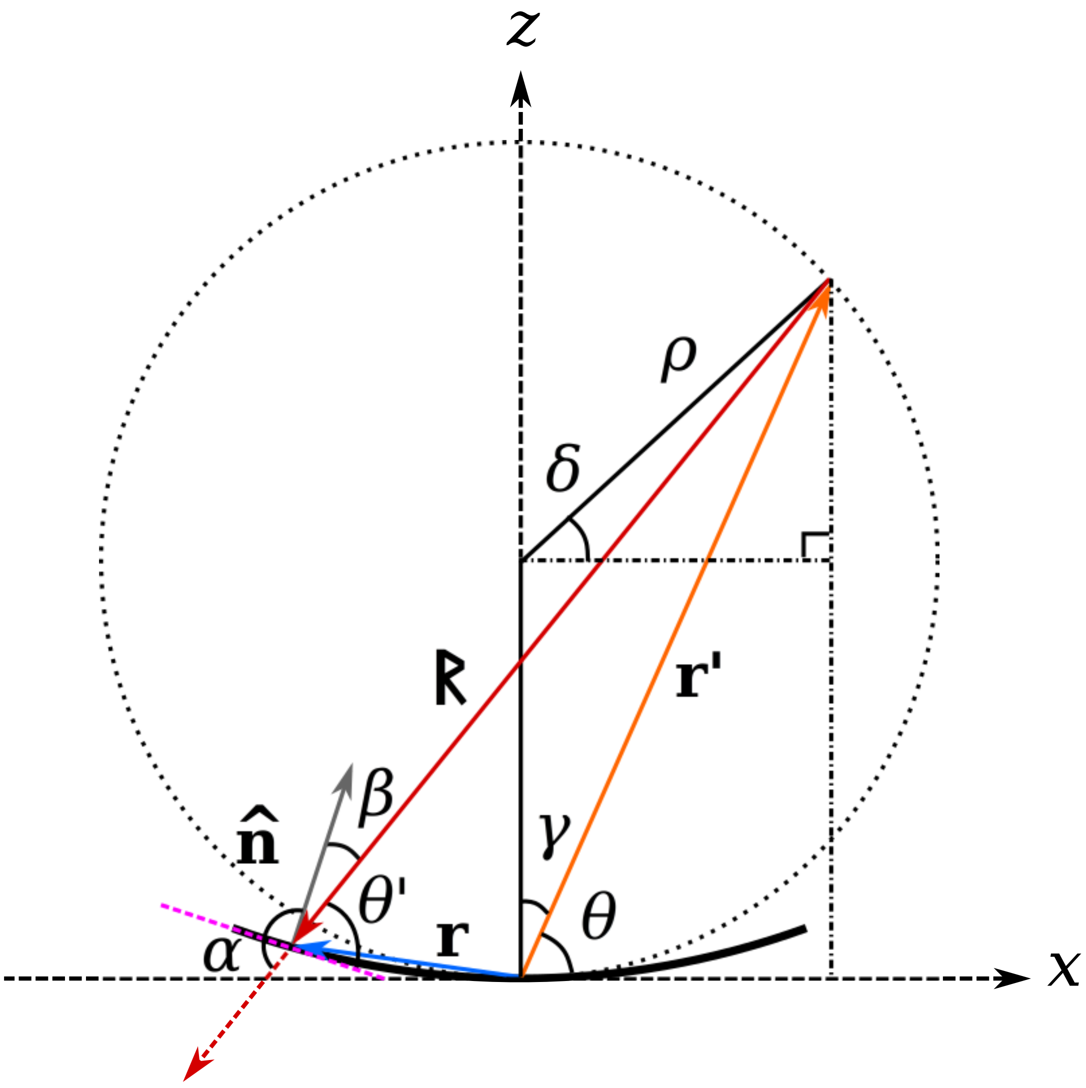

Fig. 5. Nomenclature used in the derivation of the Johann error.
